# Epidemiology of autism spectrum disorders: Global burden of disease 2019 and bibliometric analysis of risk factors

**DOI:** 10.3389/fped.2022.972809

**Published:** 2022-12-05

**Authors:** Yang-An Li, Ze-Jian Chen, Xiao-Dan Li, Ming-Hui Gu, Nan Xia, Chen Gong, Zhao-Wen Zhou, Gvzalnur Yasin, Hao-Yu Xie, Xiu-Pan Wei, Ya-Li Liu, Xiao-Hua Han, Min Lu, Jiang Xu, Xiao-Lin Huang

**Affiliations:** ^1^ Department of Rehabilitation Medicine, Tongji Hospital, Tongji Medical College, Huazhong University of Science and Technology, Wuhan China; ^2^ World Health Organization Cooperative Training and Research Center in Rehabilitation, Wuhan China; ^3^ Nursing Department, Tongji Hospital, Tongji Medical College, Huazhong University of Science and Technology, Wuhan China; ^4^ Mrs. T. H. Chan Division of Occupational Science and Occupational Therapy, University of Southern California, Los Angeles, CA United States; ^5^ Faculty of Rehabilitation Medicine, College of Xinjiang Uyghur Medicine, Xinjiang China; ^6^ Division of Physical Therapy Education, College of Allied Health Professions, University of Nebraska Medical Center, Omaha, NE, United States

**Keywords:** autism spectrum disorders, global burden disease, prevalence, incidence, disability-adjusted life-years (DALYs), epidemiology, biblimetric analysis

## Abstract

**Background:**

To explore the geographical pattern and temporal trend of autism spectrum disorders (ASD) epidemiology from 1990 to 2019, and perform a bibliometric analysis of risk factors for ASD.

**Methods:**

In this study, ASD epidemiology was estimated with prevalence, incidence, and disability-adjusted life-years (DALYs) of 204 countries and territories by sex, location, and sociodemographic index (SDI). Age-standardized rate (ASR) and estimated annual percentage change (EAPC) were used to quantify ASD temporal trends. Besides, the study performed a bibliometric analysis of ASD risk factors since 1990. Publications published were downloaded from the Web of Science Core Collection database, and were analyzed using CiteSpace.

**Results:**

Globally, there were estimated 28.3 million ASD prevalent cases (ASR, 369.4 per 100,000 populations), 603,790 incident cases (ASR, 9.3 per 100,000 populations) and 4.3 million DALYs (ASR, 56.3 per 100,000 populations) in 2019. Increases of autism spectrum disorders were noted in prevalent cases (39.3%), incidence (0.1%), and DALYs (38.7%) from 1990 to 2019. Age-standardized rates and EAPC showed stable trend worldwide over time. A total of 3,991 articles were retrieved from Web of Science, of which 3,590 were obtained for analysis after removing duplicate literatures. “Rehabilitation”, “Genetics & Heredity”, “Nanoscience & Nanotechnology”, “Biochemistry & Molecular biology”, “Psychology”, “Neurosciences”, and “Environmental Sciences” were the hotspots and frontier disciplines of ASD risk factors.

**Conclusions:**

Disease burden and risk factors of autism spectrum disorders remain global public health challenge since 1990 according to the GBD epidemiological estimates and bibliometric analysis. The findings help policy makers formulate public health policies concerning prevention targeted for risk factors, early diagnosis and life-long healthcare service of ASD. Increasing knowledge concerning the public awareness of risk factors is also warranted to address global ASD problem.

## Introduction

Autism spectrum disorder (ASD) is a series of neurodevelopmental conditions characterized by deficits in social communication and interaction, and stereotyped, repetitive patterns of sensory–motor behaviors (1). ASD is associated with heterogeneous symptomatology regarding physical, mental, neurodevelopmental, and functional disorders, which can extend to adulthood and result in a substantial burden on individuals, families, and society (2, 3). Although prevalence of autism spectrum disorders in children have been reported in countries and regions such as the USA (4, 5), China (6), India (7), Europe (8), and Asia (9), there is a lack of epidemiological estimates on prevalence, incidence and ASD-related health loss over time with risk factors (10). In 2016, the Global Research on Developmental Disabilities Collaborators reported the developmental disabilities among children younger than 5 years but not specifically focused on the all-age autism spectrum disorders (11, 12). Previous reviews provided global and regional overview of ASD prevalence and commodities, but national-level of ASD burden was not provided (13–15). Moreover, the risk factors leading to ASD were not presented in previous global epidemiological studies for ASD (16).

The Global Burden of Disease (GBD) database covers epidemiological information on the global, regional and national burden of diseases, injuries, causes of death and risk factors, providing a comprehensive way to investigate the geographical distribution and changes in ASD patterns over time (17). Analyses of current epidemiologic situations and temporal trends may help policy makers assess the global ASD burden, allocate resources, and formulate relevant policies. This information can further guide diagnosis, prevention, intervention and rehabilitation efforts for collaboration in regions with various degree of socioeconomic development (18–20).

In addition, understanding the status and frontier of the risk factors will facilitate researchers to investigate corresponding prevention approaches. Although many risk factors for ASD have been proposed, the complex causes of autism have made it difficult to link the complicated issue with a definite risk (21), which is not included in the GBD database yet. Hence, this study aimed to present the epidemiological estimates of autism spectrum disorders in terms of prevalence, incidence, and disability-adjusted life-years (DALYs) from 1990 to 2019 with GBD database. Besides, a bibliometric analysis was conducted to comprehensively analyze the research hotspots of ASD risk factors.

## Methods

### Overview

GBD Collaborator Group is a scientific council producing cutting-edge database of the global burden of diseases, injuries, and risk factors (17). GBD 2019 estimated global epidemiology covering incidence, prevalence, mortality, years lived with disability (YLDs), years of life lost (YLLs), and disability-adjusted life-years (DALYs) in 204 countries and territories grouped into 21 regions. Data utilized in this study were retrieved and publicly available from the Global Health Data Exchange (GHDx) website. The study followed the Guidelines for Accurate and Transparent Health Estimates Reporting (GATHER) recommendations (22).

Autism spectrum disorders (ASD) are characterized by persistent impairments in social communication, reciprocal interaction, and the presence of restricted, stereotypical behaviors. ASD is a non-fatal but life-long disease commencing in early childhood with overlapping neurodevelopmental causes. In the fifth edition of Diagnostic and Statistical Manual of Mental Disorders, ASD diagnostic criteria eliminated diagnostic subtypes (autistic disorder, Asperger's syndrome, and Pervasive Developmental Disabilities-Not Otherwise Specified), and designated as a single category (23).

### Statistical analysis

Statistical analysis was performed using R software (version 4.1.2). ASD burden was measured using prevalence, incidence, DALYs, and corresponding age-standardized rates in 204 countries and territories, from 1990 to 2019. A team of librarians identified multiple relevant data sources from published literature and websites. Standard methods, such as Bayesian meta-regression tool and regression methods, have been used to estimate prevalence, incidence and DALYs with uncertainty intervals (UIs) of autism spectrum disorders by location, year and sex by the GBD Collaborators using multiple modeling software (17). Based on the ordered draw of the modeling process, 95% UI, similar with confidence intervals (CI) mathematically, were identified from the epidemiological estimation methodology. The raw data were publicly available from the GBD Results Tool (https://vizhub.healthdata.org/gbd-results/). Age-standardized rate (ASR) and estimated annual percentage change (EAPC) were used to quantify temporal trends of ASD (24). Specifically, ASR was the sum of the product of the ratio of each age group (most are five years per group) with redistribution and the weight of the selected population group, divided by the total weight of the standard population. As a result, ASRs, including age-standardized prevalence rate (ASPR), age-standardized incidence rate (ASIR) and age-standardized DALY rate, could exclude the interference from variations in age distribution and population quantity. ASR was reported per 100,000 populations annually. Meanwhile, the EAPC point estimation based on ASR was employed to reflect the shifting trends of ASD burden over time. EAPC was calculated with the formula EAPC = 100 × [exp(β) − 1], where β demonstrated the secular trend of ASR in the 30 years. If the EAPC estimation and its 95% confidence interval were both above/below zero, the corresponding ASR was in an increasing/decreasing trend. Otherwise, the changing trend of ASR was deemed to be stable.

Socio-demographic index (SDI) was a comprehensive measurement of educational attainment, lagged distributed income, and total fertility rate to describe socioeconomic development status (25, 26). The countries and territories were categorized into five SDI quintiles (low, low-middle, middle, high-middle, and high). Finally, we calculated Pearson's correlation coefficient between EAPCs and ASRs and between EAPCs and SDI values in 2019, to investigate influential factors of ASR change trends (27).

Risk factor for autism spectrum disorder is multifactorial, such as genetic predispositions or environmental factors, which has not been included in the GBD database. Hence, the current study obtained literature published from January 1, 1990 to November 7, 2022 to make a preliminary bibliometric analysis. The retrieved results were analyzed using CiteSpace (version 6.1 R3), a literature visualization and analysis software developed to identify scientific progress and research frontiers of a certain field (28). The search strategy included the terms “autism” and “risk factor” from Web of Science. The literature was limited to English language, and finally 3,590 references were obtained after removing duplicates. For visualization, we selected “Top 50 levels of most occurred items from each slice” and “Pruning/pathfinder” to generate keyword clustering.

## Results

### ASD epidemiology in 2019

**Figure 1:**
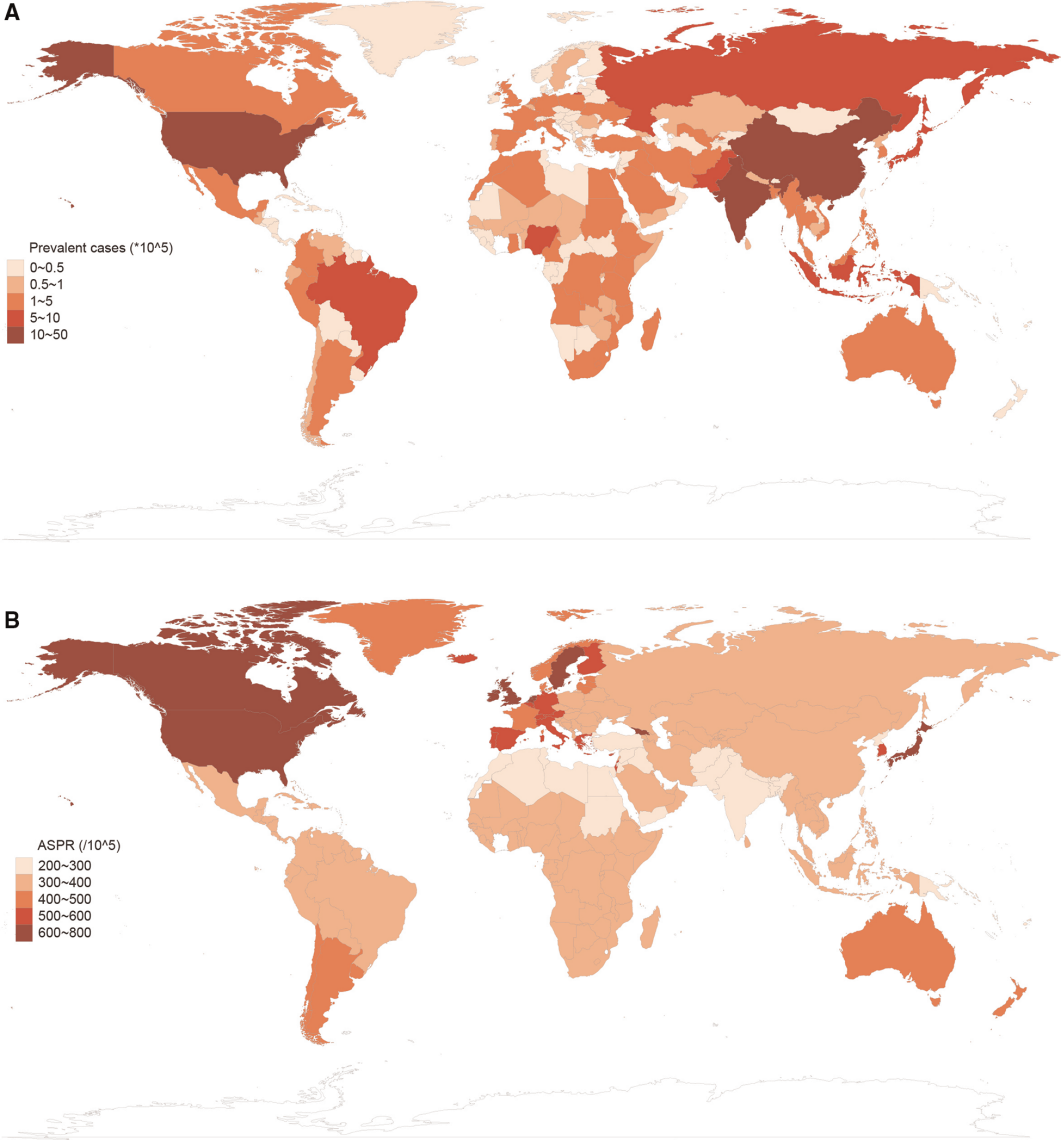
Global prevalence of autism spectrum disorders in 2019. (**A**) Prevalent cases of autism spectrum disorders by location for both sexes in 2019. (**B**) Age-standardized prevalence rate (ASPR) of autism spectrum disorders by location for both sexes in 2019.

**Figure 2:**
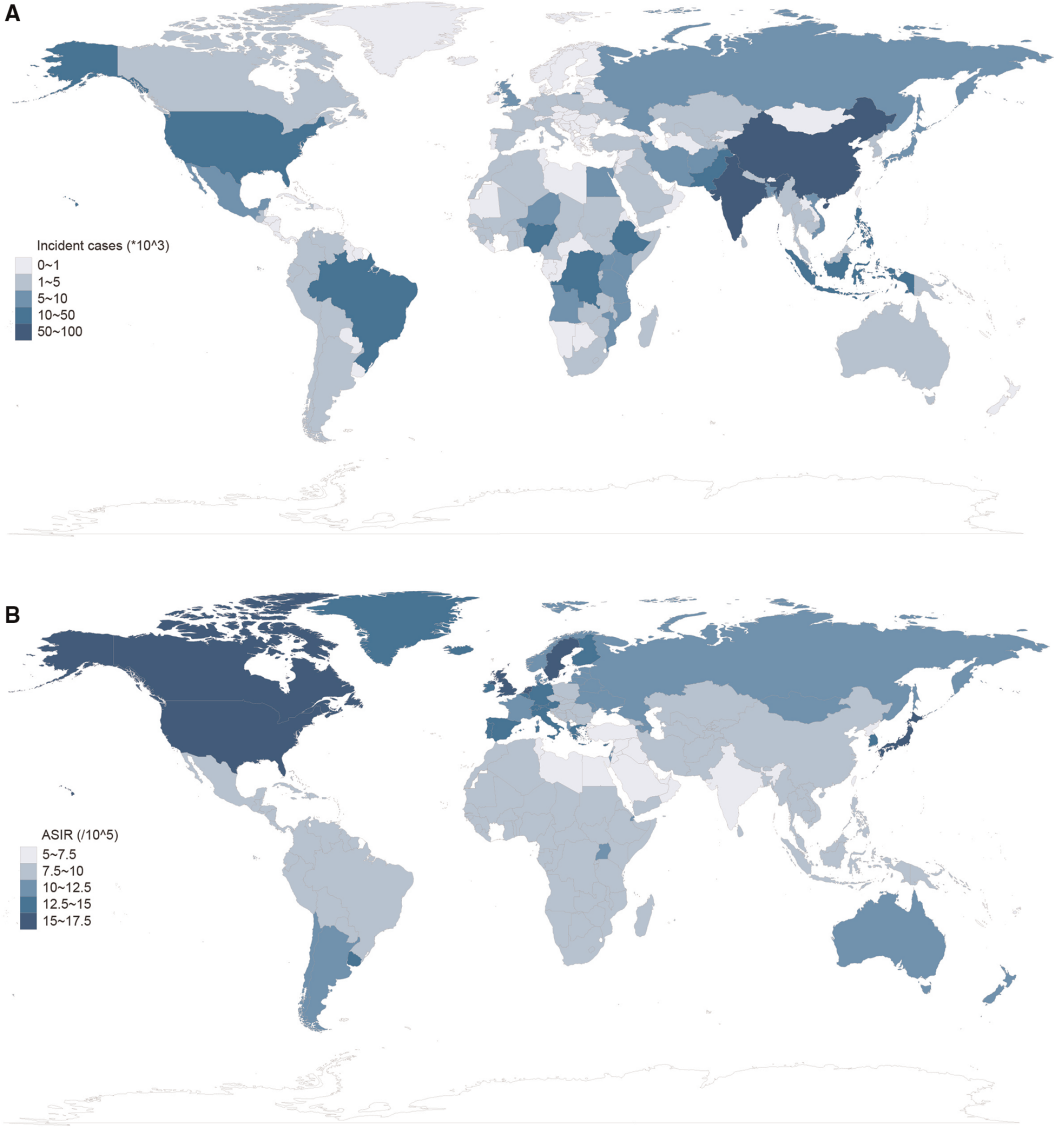
Global incidence of autism spectrum disorder in 2019. (**A**) Incident cases of autism spectrum disorders by location for both sexes in 2019. (**B**) Age-standardized incidence rate (ASIR) of autism spectrum disorders by location for both sexes in 2019.

Globally, there were estimated 28.3 million (95% UI, 23.5–33.8 million) prevalent cases of autism spectrum disorders, with an age-standardized prevalence rate of 369.4 (95%UI, 305.9 to 441.2 in 2019 (Table 1). ASD was responsible for 603,790 (95% UI, 501,680 to 720,097) incident cases globally, with an age-standardized incidence rate of 9.3 (95% UI, 7.7 to 11.1) in 2019. In addition, ASD accounted for 4.3 million (95% UI, 2.8 to 6.2) DALYs, with an age-standardized rate of 56.3 (95% UI, 36.8 to 81.5) DALYs in 2019 (Table 2). For SDI regions, the Middle SDI region had the highest burden of prevalent cases and DALYs in 2019, while the Low-middle SDI region had the most incident cases. Geographical distribution of the prevalence, incidence and DALYs estimates for autism spectrum disorders in 2019 were presented in Figures 1, 2, and Supplementary Figure S1.

**Table 1:** Number and age-standardized prevalence rate for ASD by global burden of disease region in 1990 and 2019.

	Prevalent cases No. (95% UI)		ASPR/100,000 No. (95% UI)	
	1990	2019	1990	2019
Overall	20,336,256 (16,857,367–24,222,582)	28,324,939 (23,500,644–33,811,271)	372.8 (309.1–444.9)	369.4 (305.9–441.2)
Male	15,647,724 (12,986,732–18,607,125)	21,633,776 (17,985,166–25,761,348)	571.2 (473.8–679.6)	560.1 (465.2–6667.3)
Female	4,688,533 (379,052–57,118,664)	6,691,162 (5,436,262–8,153,529)	173.4 (140.9–211.5)	176.3 (143.0–214.5)
High SDI	4,319,311 (3,613,076–5,102,134)	5,484,192 (4,587,041–6,520,432)	539.6 (451.7–638.4)	579.3 (485.3–684.5)
High-middle SDI	4,665,416 (3,863,289–5,575,606)	5,520,271 (4,566,936–6,619,212)	404.6 (334.9–483.3)	405.4 (335.9–485)
Middle SDI	5,698,916 (4,667,746–6,901,112)	7,566,752 (6,220,224–9,144,158)	319.7 (262.7–387.2)	321.4 (264.5–388)
Low-middle SDI	3,710,774 (3,057,599–4,450,285)	5,612,896 (4,621,359–6,739,732)	311.5 (257.2–373.9)	312.8 (258.2–375.7)
Low SDI	1,931,430 (1,592,707–2,329,440)	4,125,396 (3,401,890–4,965,501)	340.5 (280.2–408.3)	342.2 (281.4–410.2)
Andean Latin America	136,708 (112,528–164,539)	219,156 (180,811–263,070)	340.4 (280.9–408.5)	342.1 (282.4–410.5)
Australasia	86,986 (72,543–103,962)	119,973 (100,247–143,664)	436.8 (364.3–521.8)	436.1 (363.8–521.2)
Caribbean	159,035 (137,865–183,541)	206,648 (178,807–238,417)	344.2 (284–414.1)	343.8 (283.7–413.6)
Central Asia	10,875 (8886–13,126)	20,847 (17,026–25,167)	371.7 (305.9–446.9)	374.8 (308–450.9)
Central Europe	268,361 (220,526–321,320)	354,787 (291,432–426,875)	370.5 (305.8–443.3)	373.6 (308.4–446.9)
Central Latin America	445,113 (367,189–532,928)	393,541 (325,032–472,445)	351.7 (289.6–420.6)	350.9 (288.8–419.5)
Central sub-Saharan Africa	608,677 (502,158–726,427)	875,348 (720,122–1,047,034)	370.4 (303–446.3)	370.8 (303.3–446.9)
East Asia	4,412,839 (3,624,038–5,309,093)	5,098,806 (4,206,229–6,150,048)	353.5 (290.3–425.4)	367.8 (304.4–441.9)
Eastern Europe	866,526 (716,655–1,038,602)	773,320 (638,888–927,709)	394.8 (326.1–472.8)	397.3 (328.3–476)
Eastern sub-Saharan Africa	776,444 (638,587–926,809)	1,667,223 (1,372,086–1,991,501)	378.2 (311.7–454.2)	378.4 (311.7–454.4)
High-income Asia Pacific	1,047,349 (872,107–1,244,347)	1,078,242 (895,833–1,288,544)	617.8 (515–734.7)	634.3 (528.8–756.7)
High-income North America	1,435,440 (1,210,088–1,685,566)	2,185,918 (1,833,599–2,587,355)	526.2 (443.4–617.9)	640 (537.7–756.4)
North Africa and Middle East	1,105,582 (911,505–1,326,857)	1,879,528 (1,550,850–2,261,649)	303.5 (250.4–365.5)	304.4 (251.2–366.1)
Oceania	19,903 (16,274–23,989)	40,114 (32,728–48,523)	290 (236.8–350.4)	289 (235.5–349)
South Asia	3,370,030 (2,767,916–4,062,245)	5,310,541 (4,355,785–6,406,808)	291.9 (240.1–351.9)	290 (238.4–349.2)
Southeast Asia	1,519,341 (1,253,044–1,824,857)	2,097,143 (1,729,612–2,515,054)	310.5 (256.1–373.2)	312.5 (257.7–374.4)
Southern Latin America	240,321 (199,640–288,082)	313,298 (260,284–376,359)	480.8 (399.3–576.6)	482.5 (400.8–579)
Southern sub-Saharan Africa	204,885 (167,918–245,329)	298,595 (244,999–358,553)	369.4 (302.9–444.8)	371.6 (304.9–447.7)
Tropical Latin America	563,007 (465,390–674,324)	774,446 (638,700–930,842)	353.9 (292–425.4)	353.9 (292–425.3)
Western Europe	2,113,126 (1,773,298–2,507,729)	2,354,684 (1,967,645–2,791,090)	573.7 (482.7–679.9)	581.3 (488.2–686.4)
Western sub-Saharan Africa	769,073 (634,591–917,695)	1,807,743 (1,490,123–2,160,089)	375.3 (309.6–449)	370.6 (305.5–443.3)

ASD, autism spectrum disorders; ASPR, age-standardized prevalence rate; SDI, sociodemographic index.

**Table 2:** Number and age-standardized rates of incidence and of DALYs for ASD by global burden of disease region in 1990 and 2019.

	Incident cases No. (95% UI)		ASIR/100,000 No. (95% UI)		DALYs No. (95% UI)		Age-standardized DALY rate/100,000 No. (95% UI)	
	1990	2019	1990	2019	1990	2019	1990	2019
Overall	602,887 (501,382–718,288)	603,790 (501,680–720,097)	9.2 (7.6–10.9)	9.3 (7.7–11.1)	3,105,909 (2,025,303–4,514,467)	4,306,615 (2,821,512–6,232,361)	56.7 (37–82.2)	56.3 (36.8–81.5)
**Sex**								
Male	461,633 (386,362–548,162)	459,493 (384,472–544,406)	13.6 (11.4–16.1)	13.7 (11.5–16.3)	2,393,774 (1,560,490–3,443,926)	3,294,468 (2,152,733–4,769,103)	86.9 (56.7–125.1)	85.3 (55.8–123.6)
Female	141,254 (113,407–170,938)	144,297 (115,510–174,371)	4.4 (3.6–5.4)	4.6 (3.7–5.6)	712,135 (467,655–1,041,370)	1,012,148 (663,237–1,477,450)	26.2 (17.2–38.2)	26.8 (17.5–39.2)
**Sociodemographic index**								
High SDI	75,696 (63,921–89,058)	72,189 (60,836–84,979)	13.3 (11.3–15.7)	14.5 (12.3–17.1)	654,789 (429,489–932,431)	822,967 (541,875–1,175,034)	82.2 (53.8–117.4)	88.2 (57.9–126.3)
High-middle SDI	100,983 (83,515–120,117)	77,479 (64,215–92,332)	10.2 (8.4–12.1)	10.3 (8.6–12.3)	713,059 (465,214–1,032,597)	838,198 (546,345–1,211,677)	61.8 (40.3–89.5)	62.1 (40.4–89.8)
Middle SDI	173,883 (143,431–208,737)	139,286 (114,750–167,032)	8.4 (6.9–10.1)	8.1 (6.7–9.7)	875,927 (569,681–1,284,765)	1,155,444 (754,951–1,679,972)	48.8 (31.8–71.5)	49.2 (32.1–71.6)
Low-middle SDI	148,418 (122,088–177,306)	137,109 (1,126,789–163,455)	8.2 (6.7–9.8)	8.1 (6.6–9.6)	566,661 (372,963–827,871)	856,771 (561,754–1,245,320)	47.2 (31–68.5)	47.6 (31.2–69.1)
Low SDI	103,577 (85,506–123,644)	151,149 (124,811–180,571)	9.2 (7.6–10.9)	8.37 (6.9–10.0)	293,883 (191,832–427,362)	630,885 (414,559–917,940)	51.3 (33.6–74.5)	51.9 (34.1–75.2)
**Region**								
Andean Latin America	5,235 (4322–6299)	5,659 (4672–6810)	9.1 (7.5–10.9)	9 (7.4–10.8)	20,989 (13,726–30,731)	33,571 (22,051–48,663)	51.9 (33.9–75.6)	52.3 (34.4–75.9)
Australasia	1,669 (1396–1988)	1,957 (1636–2333)	11 (9.2–13.1)	11.1 (9.3–13.2)	13,166 (8,637 –18,709)	18,038 (11,831–26,144)	66.4 (43.6–94.3)	66.3 (43.5–96.1)
Caribbean	2,918 (2537–3357)	2,745 (2369–3134)	15 (13–17.2)	15.2 (13.1–17.3)	24,224 (15,958–33,906)	31,128 (21,014–44,022)	91.6 (60.3–129)	92.9 (62.4–131.9)
Central Asia	596 (491–717)	923 (759–1109)	9.9 (8.1–11.9)	9.8 (8.1–11.8)	1,646 (1092–2399)	3,173 (2084–4577)	55.1 (36.5–80.7)	55.3 (36.5–80)
Central Europe	9,227 (7599–11,021)	8,923 (7349–10,654)	9.9 (8.1–11.8)	9.9 (8.1–11.8)	41,257 (26,948–60,088)	54,402 (35,733–78,754)	56.9 (37.3–82.7)	57.4 (37.7–83.2)
Central Latin America	7,550 (6234–8948)	4,860 (4014–5764)	9.4 (7.8–11.1)	9.4 (7.8–11.2)	67,585 (43,770–97,177)	59,276 (38,737–85,387)	56.5 (36.6–81.2)	57.1 (37.2–82)
Central sub-Saharan Africa	21,860 (18,043–26,050)	19,340 (15,980–23,070)	9.2 (7.6–11)	9.2 (7.6–10.9)	93,735 (61,359–134,650)	133,793 (87,313–194,854)	53.7 (35.1–77.3)	53.6 (35–78.2)
East Asia	111,604 (91,661–133,843)	71,074 (58,298–84,934)	9.3 (7.6–11.1)	9.6 (7.9–11.5)	680,072 (444,396–996,214)	778,339 (509,697–1,129,871)	54.3 (35.4–79.6)	56.6 (37–82.7)
Eastern Europe	14,259 (11,834–17,033)	11,166 (9244–13,324)	10.2 (8.4–12.1)	10.3 (8.5–12.3)	131,359 (85,365–190,703)	116,804 (76,389–169,067)	60.2 (39.2–87.3)	60.8 (39.6–87.8)
Eastern sub-Saharan Africa	42,923 (35,394–51,307)	67,380 (55,602–80,554)	10.1 (8.3–12.1)	10 (8.2–11.9)	118,378 (77,519–171,365)	255,582 (168,416–369,498)	57.1 (37.3–82.6)	57.4 (37.7–83.1)
High-income Asia Pacific	14,603 (12,198–17,337)	10,412 (8699–12,345)	15.4 (12.9–18.3)	15.7 (13.1–18.6)	159,572 (104,216–229,855)	162,304 (106,518–233,835)	94.5 (61.6–136.4)	97.3 (63.6–141)
High-income North America	29,858 (25,081–35,106)	33,093 (27,800–38,830)	13.6 (11.4–16)	16.4 (13.8–19.3)	216,781 (142,092–307,646)	326,234 (215,047–462,932)	79.9 (52.3–113.3)	96.9 (63.7–137.7)
North Africa and Middle East	44,221 (36,225–53,111)	45,002 (36,857–53,912)	7.9 (6.5–9.5)	7.7 (6.3–9.3)	170,028 (110,895–247,697)	287,617 (188,299–418,662)	46.3 (30.2–67.6)	46.4 (30.4–67.5)
Oceania	808 (661–977)	1,506 (1228–1822)	7.6 (6.2–9.2)	7.6 (6.2–9.3)	3,050 (1985–4418)	6,136 (3980–8860)	44 (28.7–63.9)	43.9 (28.6–63.2)
South Asia	129,806 (106,720–155,432)	121,886 (100,113–145,614)	7.6 (6.2–9)	7.6 (6.2–9.1)	512,702 (337,803–748,333)	808,171 (530,435–1,176,149)	44 (29–64.4)	44 (28.9–63.9)
Southeast Asia	49,221 (40,292–58,767)	42,761 (34,987–51,276)	8.2 (6.7–9.7)	8.2 (6.7–9.8)	233,298 (151,876 –343,401)	321,010 (208,179–470,066)	47.3 (30.9–69.5)	47.8 (31–70.1)
Southern Latin America	6,094 (5094–7271)	5,784 (4838–6900)	12.1 (10.2–14.5)	12.5 (10.4–14.9)	36,786 (23,775–52,798)	47,678 (30,967–68,855)	73.5 (47.5–105.4)	73.7 (47.9–106.2)
Southern sub-Saharan Africa	7,211 (5956–8603)	7,815 (6468–9334)	9.8 (8.1–11.7)	9.8 (8.1–11.7)	31,425 (20,608–45,813)	45,505 (29,825–65,836)	56.2 (36.7–81.3)	56.4 (37–81.4)
Tropical Latin America	15,756 (13,009–18,760)	14,382 (11,894–17,114)	9.2 (7.6–11)	9.3 (7.7–11.1)	86,132 (56,209–124,848)	117,552 (76,819–170,312)	53.8 (35.2–78.2)	53.9 (35.2–78)
Western Europe	31,691 (26,879–37,291)	29,331 (24,871–34,482)	14.2 (12.1–16.7)	14.2 (12–16.7)	319,676 (208,840–454,860)	353,845 (233,430 –504,526)	87.5 (57.1–125)	88.7 (58.1–127.1)
Western sub-Saharan Africa	42,800 (35,511–51,287)	77,149 (64,001–92,454)	10 (8.3–12)	9.8 (8.2–11.8)	117,111 (76,701–169,033)	276,573 (181,323–400,818)	56.7 (37.2–81.6)	56.2 (36.9–81.3)

ASD, autism spectrum disorders; ASIR, age-standardized incidence rate; DALY, disability-adjusted life year; SDI, sociodemographic index.

### Temporal trends of ASD prevalence from 1990 to 2019

**Figure 3:**
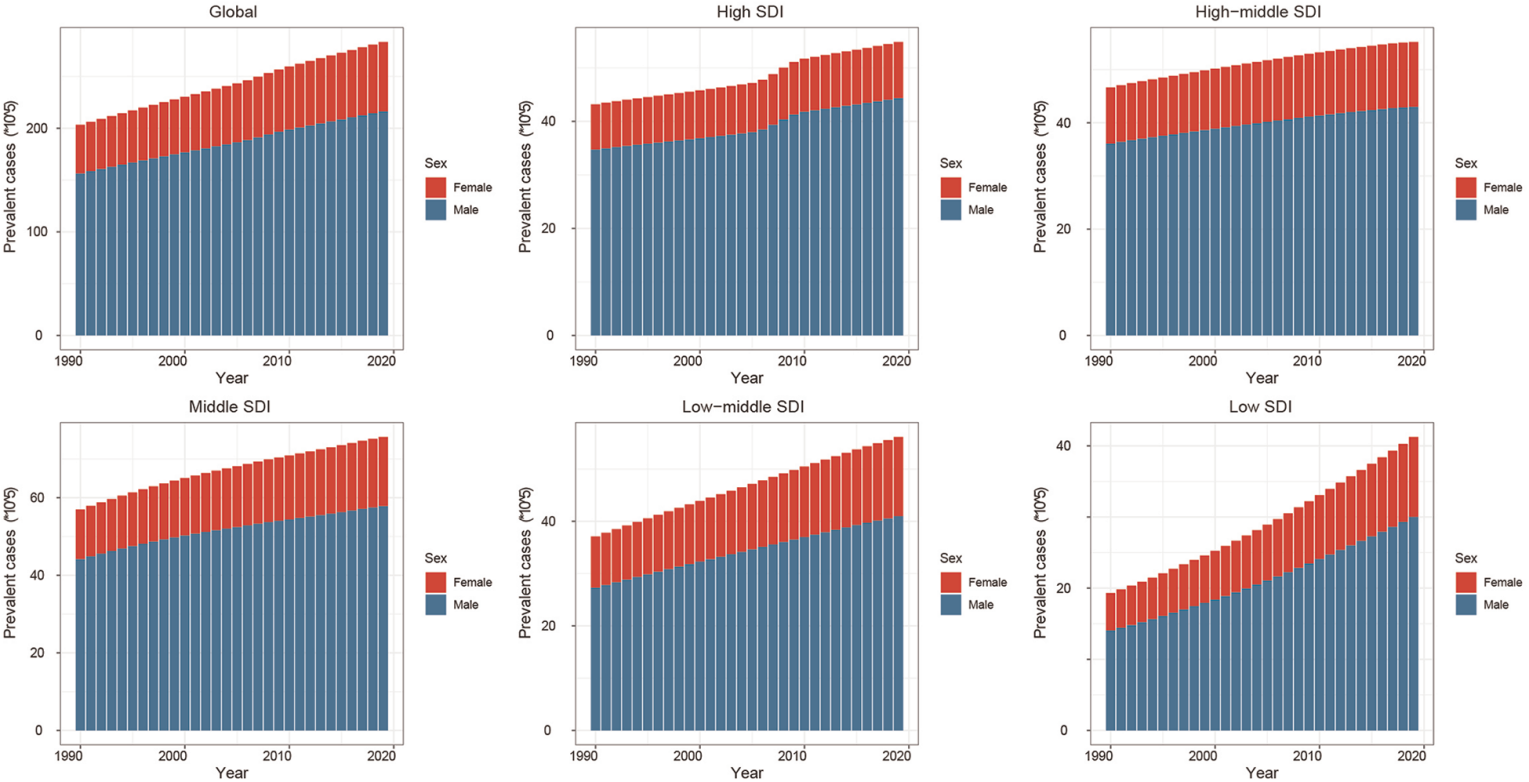
Global prevalence of autism spectrum disorders (ASD) by sex and socio-demographic index (SDI) quintiles from 1990 to 2019.

From 1990 to 2019, the global ASD prevalent cases increased by 39.3% and the age-standardized prevalence rate has not almost improvement [EAPC = −0.02, 95% CI (−0.03 to −0.01), Supplementary Table S1]. Males were more likely to have ASD than females (male to female ratio in ASIR = 3.34:1 in 1990, and 3.23:1 in 2019). At the regional level, the age-standardized prevalence rate of ASD was found to be highest in high-income North America [640.0 (95% UI, 537.7 to 756.4)], high-income Asia Pacific [634.3 (95% UI, 528.8 to 756.7)] and Western Europe [581.3 (95% UI, 488.2 to 686.4)] (Table 1). Subgroup analysis by SDI regions demonstrated that although high SDI region had the most rapid increase in prevalence (ASPR: 539.6 in 1990 and 579.3 in 2019, EAPC = 0.30, 95% CI 0.25 to 0.35) (Figure 3, Supplementary Table S1). At the level of country or territory, there was wide geographic variation in the ASPR of ASD (range, 215.8 to 739.6). United Kingdom [739.6 (95% UI, 617.2 to 876.3)], Sweden [706.8 (95% UI, 589.1 to 838.5)] and Japan [676.5 (95% UI, 562.8 to 805)] had the three highest ASPR in 2019 (Figure 1, Supplementary Tables S1, S5).

### Temporal trends of ASD incidence from 1990 to 2019

From 1990 to 2019, the global ASD incidence has not been improved since incident cases increased marginally by 0.1% and the age-standardized prevalence rate increased by 1.1% [EAPC = 0.06, 95% CI (0.04 to 0.07), Supplementary Table S2]. Females had fewer ASD incidence than males (1990: 461,633 in males and 141,254 in females; 2019: 459,493 in males and 144,297 in females). At the regional level, the age-standardized incidence rate of ASD was highest in high-income North America 16.4 (95% UI, 13.8 to 19.3), high-income Asia Pacific 15.7 (95% UI, 13.1 to 18.6) and Caribbean 15.2 (95% UI, 13.1 to 17.3) (Table 2). For SDI regions, the high SDI region had the most increase in age-standardized incidence rate from 1990 to 2019 (change = 9%; EAPC = 0.36, 95% CI 0.31 to 0.41, Supplementary Table S2). At the level of country or territory, ASIR varied from 5.4 to 18.6. Andorra [18.6 (95% UI, 15.3 to 22.3)], United Kingdom [18.0 (95% UI, 15 to 21.2)] and Sweden [17.1 (95% UI 14.4 to 20.2)] had the three highest age-standardized prevalence rates in 2019 (Figure 2, Supplementary Tables S2 and S5).

### Temporal trends of ASD DALYs from 1990 to 2019

From 1990 to 2019, the global ASD DALYs increased by 38.7% and the age-standardized DALY rate has almost no improvement [EAPC = −0.02, 95% CI (−0.03 to −0.01), Supplementary Table S3]. The number of global DALYs in males was higher than in females (1990: 2393774.2 in male and 712134.9 in females; 2019: 3294467.6 in males and 1012147.8 in females). At the regional level, the age-standardized DALYs rate of ASD was found to be highest in high-income Asia Pacific 97.3 (95% UI, 63.6 to 141), high-income North America 96.9 (95% UI, 63.7 to 137.7) and Caribbean 92.9 (95% UI, 62.4 to 131.9) (Table 2). Subgroup analysis by SDI regions demonstrated that although the high SDI region had the most rapid increase in DALYs (age-standardized DALY rate: 82.2 in 1990 and 88.2 in 2019, EAPC = 0.81, 95% CI 0.68 to 0.95, Supplementary Table S3). At the level of country or territory, age-standardized incidence DALYs rates varied from 33.3 to 112.3 among the countries and territories. United Kingdom [112.3 (95% UI, 73.7 to 160.5)], Sweden [108.0 (95% UI, 71.2 to 155.3)] and Japan [103.8 (95% UI, 67.7 to 149.7)] showed the highest age-standardized DALYs rates in 2019 (Supplementary Figure S1, Supplementary Tables S3 and S4).

### Correlation between the socio-demographic Index and ASD epidemiology

Pearson's correlation coefficients between ASRs in 1990 and the corresponding EAPC values were not statistically significant (Supplementary Figure S6). We explored the association between SDI in 2019 and EAPC values of ASPR, ASIR, and age-standardized DALY rate among the countries and territories. The results demonstrated that the associations between SDI, and EAPCs of ASIR and age-standardized DALY rate, were not statistically significant. However, the EAPC value of ASPR was positively associated with SDI in 2019 (Supplementary Figure S7). Further, we investigated the correlation between SDI and ASPR, ASIR, and age-standardized DALY rate from 1990 to 2019. As a result, all ASRs were significantly and positively correlated with corresponding SDI values (correlation coefficient of ASPR = 0.672, of ASIR = 0.638, of age-standardized DALY rate = 0.681, *P* < 0.05) in 21 regions worldwide. For most countries and territories, after a decrease in expected ASRs, these rates increased rapidly when SDI values were higher than 0.4 in 2019 (Supplementary Figures S8–S10).

### Bibliometric analysis

**Figure 4:**
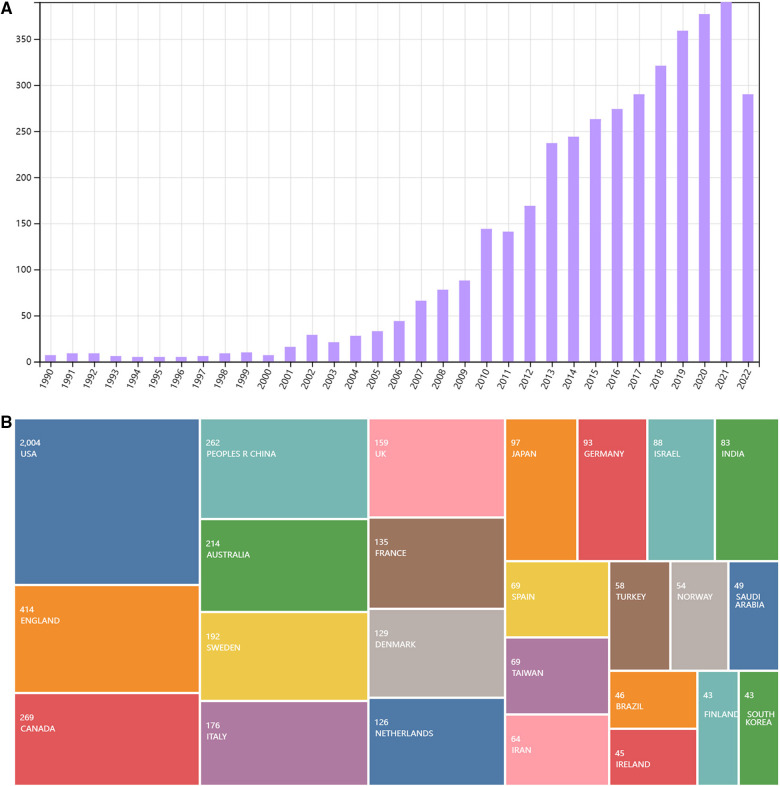
(**A**) The number of publications on risk factors of ASD since 1990. (**B**) Leading countries or territories that contributed to publications on risk factors of ASD since 1990.

A total of 3,991 articles in English were retrieved from Web of Science. After deleting duplicate literatures, 3,590 references were obtained for risk factors of ASD. As presented in Figure 4A, the overall number has been increasing since 1990. Among the countries/territories, USA, England, Canada, China, and Australia make leading contributions to exploring ASD risk factors in the past decades (Figure 4B).

### ASD risk factors-research categories

**Figure 5:**
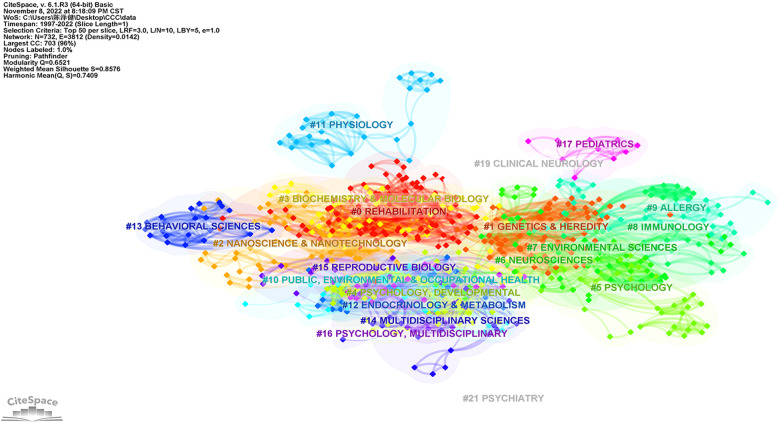
The cluster map of co-occurrence research categories related to risk factors on ASD since 1990.

In the current study, we identified the top research categories with citations analyzed by the CiteSpace software. As demonstrated in Figure 5, research category was represented a cloud of circular nodes, with the areas depicting the number of research literature in each field. Moreover, the node orientations demonstrated the publication's importance. As a result, “#0 Rehabilitation”, “#1 Genetics & Heredity”, “#2 Nanoscience & Nanotechnology”, “#3 Biochemistry & Molecular biology”, “#4/5 Psychology”, “#6 Neurosciences”, and “#7 Environmental Sciences” may have played pivotal roles in the field of ASD risk factors since 1990.

### ASD risk factors-keywords clustering

**Figure 6:**
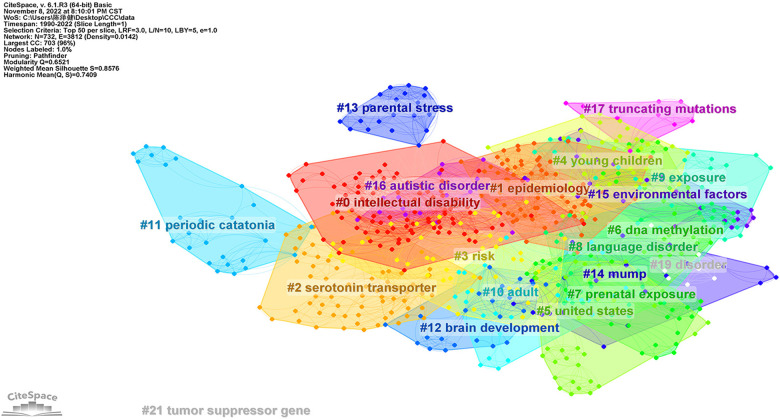
The cluster map of co-occurrence keywords related to risk factors on ASD since 1990.

**Figure 7:**
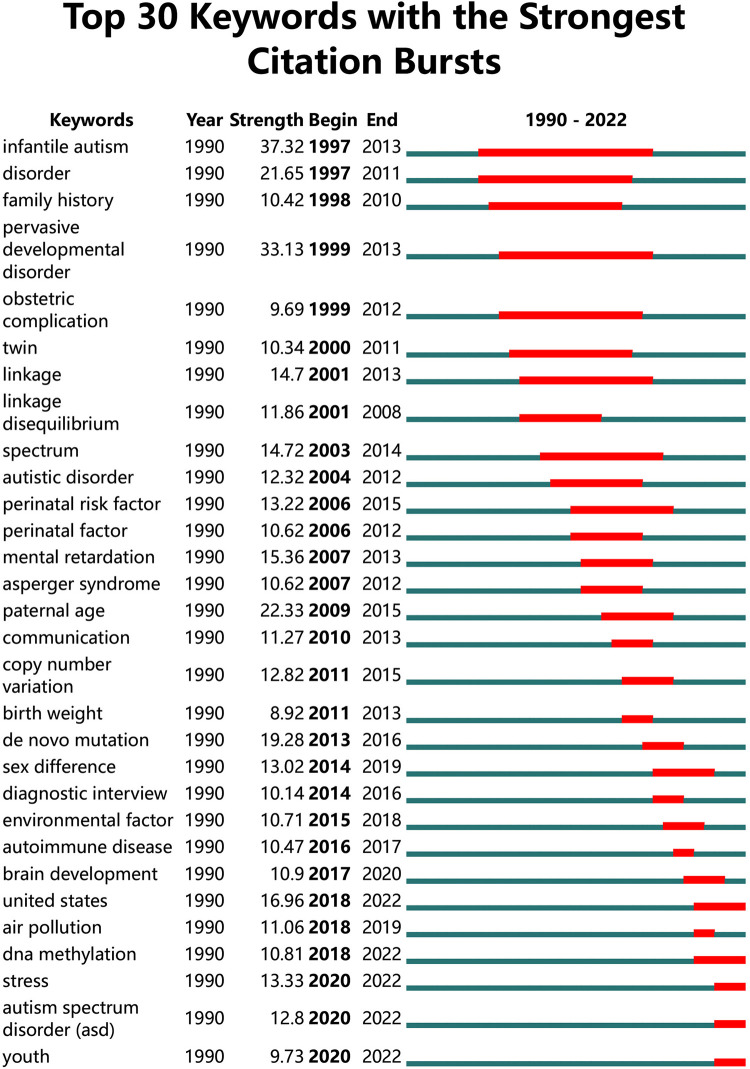
The top 30 keywords with the strongest citation bursts related to risk factors on ASD since 1990.

We use the default setting of CiteSpace (Slice length = “1” year; Select the node type = “Keyword”; Top *N* = “50”) and a pruning algorithm to cluster the keywords. After processing, top keywords with the strongest co-occurrence frequency were found to understand hotspots in this field since 1990. As shown in the Figure 6, the Mean Silhouette score was 0.8576, and the Modularity Q score was 0.7409. The emerging clusters revealed that ASD risk factors could be multifaceted and there is no definite conclusion. Hence, we used Burst Keywords analysis to explore the risk factor receiving high attention in the past decades (Figure 7). For example, “Pervasive developmental disorder”, “obstetric complication”, and “perinatal factor” were of the earliest burst. In recent years, “de novo mutation”, “environmental factor”, and “DNA methylation” have been among the most focused keywords.

## Discussion

In this study, we presented the epidemiological estimates and temporal trends of autism spectrum disorders based on the GBD database at the global, regional, and national levels from 1990 to 2019. Globally, there were estimated 28.3 million prevalent cases (ASR, 369.4 per 100,000 populations), 603,790 incident cases (ASR, 9.3 per 100,000 populations) and 4.3 million DALYs (ASR, 56.3 per 100,000 populations) in 2019. From 1990 to 2019, the prevalent cases and DALYs of ASD increased by almost 40%, while incidence and the ASRs have not been improved. We found the ASRs were significantly higher in the high SDI region, and the ASRs increased as SDI increased except the low SDI region. Moreover, we identified the hotspots concerning ASD risks factors since 1,900 because it is not provided in the GBD database.

Understanding the epidemiology of ASD burden facilitates tailoring health programs to address this great health challenge. Studies have shown that ASD was the leading cause of disability among all mental disorders in children globally but lack of knowledge concerning its geographical distribution in all-age populations (11, 29). A systematic review on ASD prevalence and DALYs found estimated 52 million prevalent cases (ASR, 760) and 7.7 million DALYs (ASR, 58.2) of ASD worldwide in 2010 (13). However, it is impracticable to compare with our report because of substantial differences in methodologies and data sources. The review included much epidemiological data from high-income countries, lack in low-income and middle-income countries (LMICs), especially Africa, Latin America and Central and Eastern Europe (10, 13). At the national level, the age-standardized prevalence rates ranged from 215.8 to 739.6 per 100,000 populations in our study, which were greatly lower than previous reports (30). The reason for the considerable but not contradictory differences mainly lied in the study population: our study highlighted the all-age populations while most epidemiological studies were only concerned with children and young adolescents. As the incident rate has been increasing but tending to be stable (EAPC = 0.06), the overall ASPR could have been attenuated by the underemphasized age groups (Supplementary Table S5). Besides, data sources could have led to heterogeneous results, such as data derived from health records or parents' report, and from nationally representative population or selected sites (5, 31, 32). Our study also showed a male preponderance in ASRs similar to previous studies, which were related to complicated but unknown mechanisms, including environmental, genetic or epigenetic reasons (13, 33, 34).

Monitoring the temporal trend of ASD burden helps find the emerging challenges and adapt health systems over time. Globally, the ASD prevalent cases and DALYs increased by almost 40% in the past 30 years, while the ASRs and incidence tended to be stable. Such increases may be mainly attributable to the global population growth, instead of change in ASD incidence (29). As the ASRs were deemed unchanging over time, etiological research and primary prevention for reducing ASD incidence deserve continuous attention for a long time to come (30). Furthermore, ASD symptoms could extend beyond childhood and persist across lifespan although it was commonly perceived to be childhood disorders (35). Hence, the epidemiological data in all-age populations, including children, adolescents and adults, should draw attention when considering the increasing burden over time. Meanwhile, medical resources on the topic of early diagnosis and therapy are crucial for secondary prevention of permanent disabilities during golden period sensitive to treatment (36). Additionally, regions especially those with high prevalent cases, such as China, India and USA, should formulate policies to support long-term healthcare service, education, skills training and vocational assistance for rehabilitation (tertiary prevention) and reducing stigmatization (37, 38).

We investigated the correlation between social development status and ASD burden, which may shed a light on ASD prevention and international cooperation to address disease burden. The causes of ASD were unrevealed but usually attributed to complex genetics-environment interactions. According to the results, all ASRs were positively correlated with SDI values from 1990 to 2019. For most countries and territories, after a decrease in expected ASRs, these rates increased rapidly when SDI values were higher than 0.4 in 2019. The positive correlation was underpinned by the view that awareness, concepts, and healthcare service availability of ASDs could be improved with social development (39). Moreover, environmental exposure, such as air pollution and endocrine-disrupting chemicals, was accompanied with socioeconomic development in the past decades (30). However, there was a paradox when attributing to socioeconomic status, because ASD burden in low SDI region did not fit the association. This result might be explained by non-etiologic reasons in LMICs, such as public awareness since the diagnosis was based on social and contextual observations (40, 41) or etiologic factors such as genetic and environmental risk factors (30).

ASD is a complex multifactorial disease, and it needs interdisciplinary cooperation to find out the linking causes with the issue, such as Genetic & Heredity, Biochemistry, Public health and Environmental sciences, etc. For example, parental age, environmental exposure, *de novo* mutations and epigenetic alterations were shown associated with ASD risk (1, 21, 42). After bibliometric analysis, we found literature on risk factors for ASD shows a growing trend since 1999. Moreover, some emerging research directions could provide new perspective on ASD etiology. Previous studies found that allergic diseases are over-represented in ASD and hypothesized that immune dysregulation may have contributed to autism pathogenesis (43). Furthermore, gut microbiome can modulate gastrointestinal physiology and immune system relevant to ASD symptoms (44). More specifically, we can inspect the change of research hotspots from keywords with citation burst over time. Earlier bursts indicated that previous studies focused on family history, developmental psychology, and prenatal factors. In recent years, advances in technology have led to further exploration into genetics, immune systems, and environmental factors (30, 45, 46).

This study has several limitations. First, although the GBD provides spatiotemporal estimates of disease burden for geographical location with sparse data, the accuracy and reliability of modeling rely on the quality of data used in the study, thus the epidemiological estimates should be interpreted with cautions (17). Second, the study included literature only from Web of science. Future study could include articles in multiple databases with various languages to reduce bias. Third, due to the restrictions of data type, further investigation stratified by pathophysiology, etiology, and disease severity should be conducted in future studies (47). Considering that different ASD phenotypes can be associated with psychiatric, mental, and physical disorders (14, 15), GBD models should include monitoring comorbidities to address the overall and life-long needs of individuals with autism spectrum disorders.

## Conclusions

Autism spectrum disorders remain a global public health problem. The global prevalent cases and DALYs of ASD increased greatly with population growth, while age-standardized rates and incidence has not been improved from 1990 to 2019. The increased ASRs were associated with higher sociodemographic status except the low SDI region, although which was the main contributor to the rapid increases in prevalent cases and DALYs. The epidemiological findings could help policy makers illustrate the global health challenge to formulate policies and implement measures for prevention from risk factors, early diagnosis, and life-long healthcare service. Increasing knowledge concerning the public awareness, risk factors, diagnostic criteria and interventions are also warranted to reduce ASD burden.

## Data Availability

The original contributions presented in the study are included in the article/**Supplementary Material**, further inquiries can be directed to the corresponding author/s.
