# Supplementary material for: Epidemiology of autism spectrum disorders: Global burden of disease 2019 and bibliometric analysis of risk factors
Source: Front Pediatr. 2022 Dec 5;10:972809. doi: 10.3389/fped.2022.972809 (PMC9760802; doi:10.3389/fped.2022.972809)
Supplement: Supplementary file 2 [file Datasheet2.pdf]

Supplementary Online Content

Prevalence, incidence, and disability-adjusted life-years of Autism Spectrum Disorders 1990–2019: Findings from the Global Burden of Disease study 2019

eFigure 1. Global disability-adjusted life-year (DALY) of autism spectrum disorders (ASD) in 2019. (A) DALYs of ASD by location for both sexes in 2019. (B)Age-standardized DALY rate of ASD by location for both sexes in 2019.

eFigure 2. Global prevalence of autism spectrum disorders (ASD) in 1990. (A) Prevalent cases of ASD by location for both sexes in 1990. (B)Age-standardized prevalence rate (ASPR) of ASD by location for both sexes in 1990.

eFigure 3. Global incidence of autism spectrum disorders (ASD) in 1990. (A) Incident cases of ASD by location for both sexes in 1990. (B)Age-standardized incidence rate (ASIR) of ASD by location for both sexes in 1990.

eFigure 4. Global disability-adjusted life-year (DALY) of autism spectrum disorders (ASD) in 1990. (A) DALYs of ASD by location for both sexes in 1990. (B)Age-standardized DALY rate of ASD by location for both sexes in 1990.

eFigure 5. Global disability-adjusted life-year (DALY) of autism spectrum disorders (ASD) by sex and sociodemographic index (SDI) quintiles from 1990 to 2019.

eFigure 6. The correlation analyses of EAPCs-ASR (1990). (A) The correlation between EAPC of ASPR and ASPR of 1990 by location for both sexes. (B) The correlation between EAPC of ASIR and ASIR of 1990 by location for both sexes. (C) The correlation between EAPC of age-standardized DALY rate and ASDR of 1990 by location for both sexes. Note: ASD, autism spectrum disorders; ASPR, age-standardized prevalence rate; ASIR, age-standardized incidence rate; DALY, disability-adjusted life year; EAPC, estimated annual percentage change; SDI, sociodemographic index.

eFigure 7. The correlation analyses of EAPCs-SDI (2019). (A) The correlation between EAPC of ASPR and SDI of 2019 by location for both sexes. (B) The correlation between EAPC of ASIR and SDI of 2019 by location for both sexes. (C) The correlation between EAPC of age-standardized DALY rate and SDI of 2017 by location for both sexes. The size of circle represents the quantity of ASD patients in one country or territory. Note: ASD, autism spectrum disorders; ASPR, age-standardized prevalence rate; ASIR,

age-standardized incidence rate; DALY, disability-adjusted life year; EAPC, estimated annual percentage change; SDI, sociodemographic index.

eFigure 8. The change trends and correlation analyses of age-standardized prevalence rate (ASPR) and sociodemographic index (SDI) in 21 regions from 1990 to 2019.

eFigure 9. The change trends and correlation analyses of age-standardized incidence rate (ASIR) and sociodemographic index (SDI) in 21 regions from 1990 to 2019.

eFigure 10. The change trends and correlation of age-standardized disability-adjusted life year (DALY) rate and sociodemographic index (SDI) in 21 regions from 1990 to 2019.

eTable 1. Number and age-standardized prevalence rate for ASD globally and by SDI group, GBD region and super-region, country, and territory in 1990 and 2019.

eTable 2. Number and age-standardized rates of incidence for ASD globally and by SDI group, GBD region and super-region, country, and territory in 1990 and 2019.

eTable 3. Number and age-standardized rates of DALYs for ASD globally and by SDI group, GBD region and super-region, country, and territory in 1990 and 2019.

eTable 4. Top 20 countries with autism spectrum disorders (ASD) in 2019.

eTable 5. Prevalence, incidence, and disability-adjusted life year rates by age group in 1990 and 2019.

eFigure 1. Global disability-adjusted life-year (DALY) of autism spectrum disorders (ASD) in 2019.

(A) DALYs of ASD by location for both sexes in 2019.

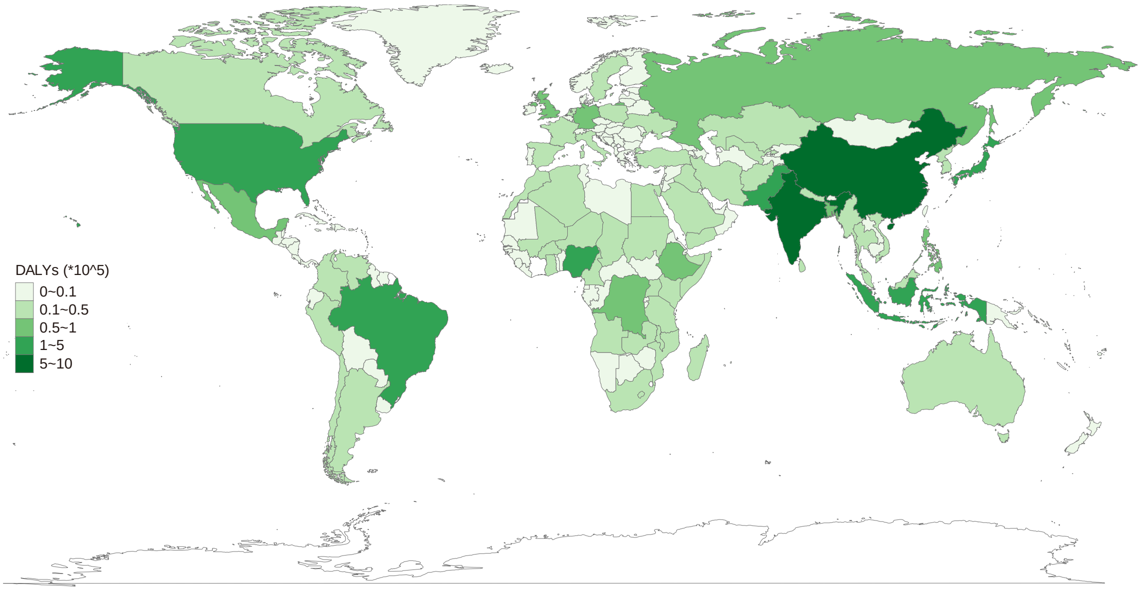

(B) Age-standardized DALY rate of ASD by location for both sexes in 2019.

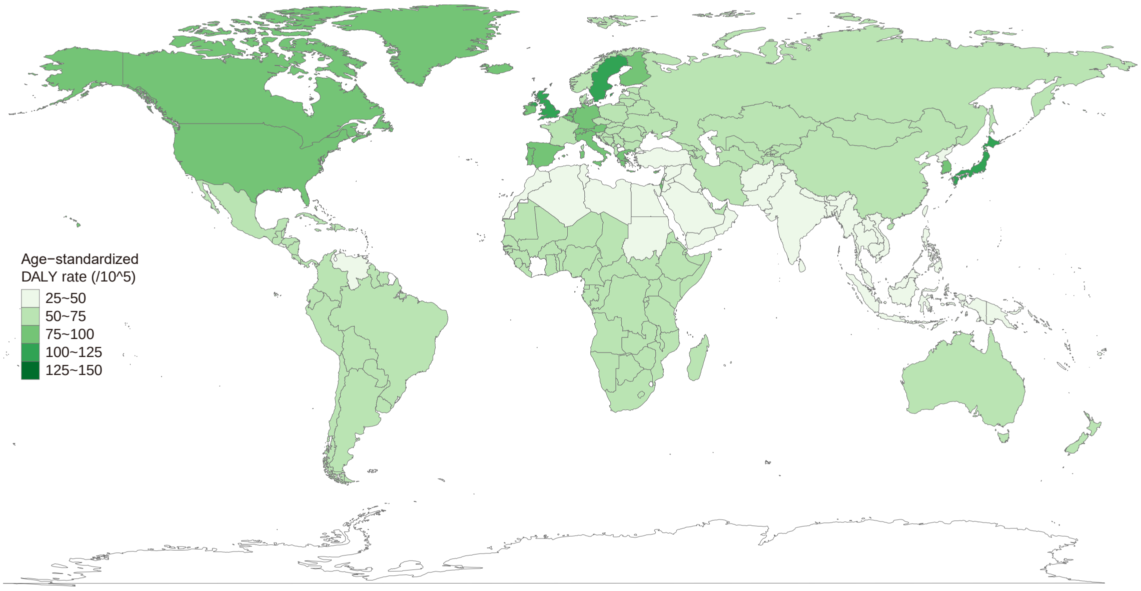

eFigure 2. Global prevalence of autism spectrum disorders (ASD) in 1990.

(A) Prevalent cases of ASD by location for both sexes in 1990.

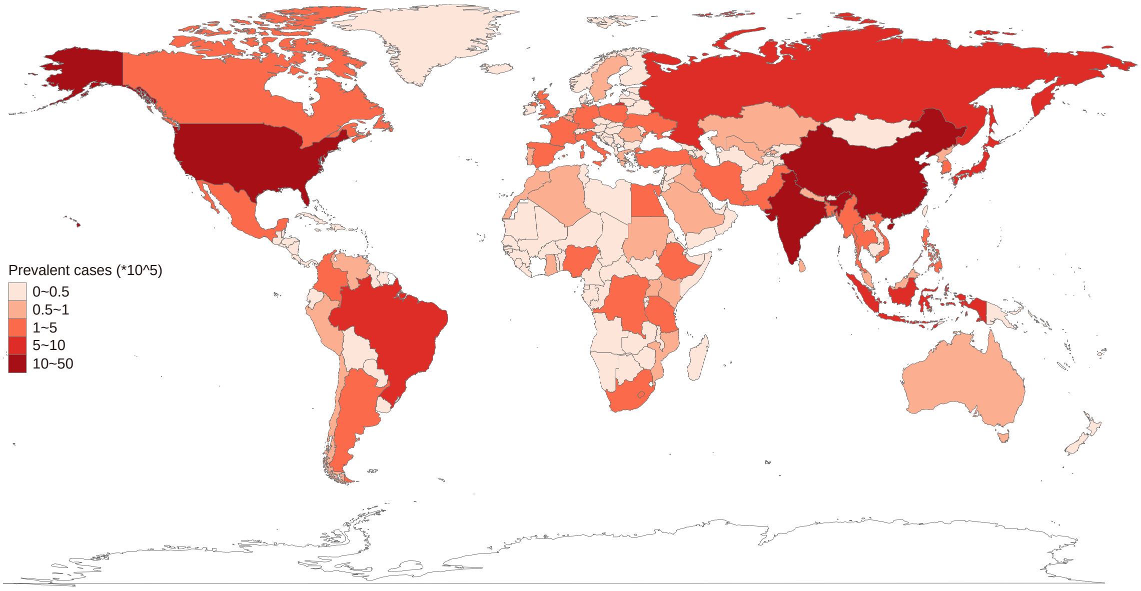

(B) Age-standardized prevalence rate (ASPR) of ASD by location for both sexes in 1990.

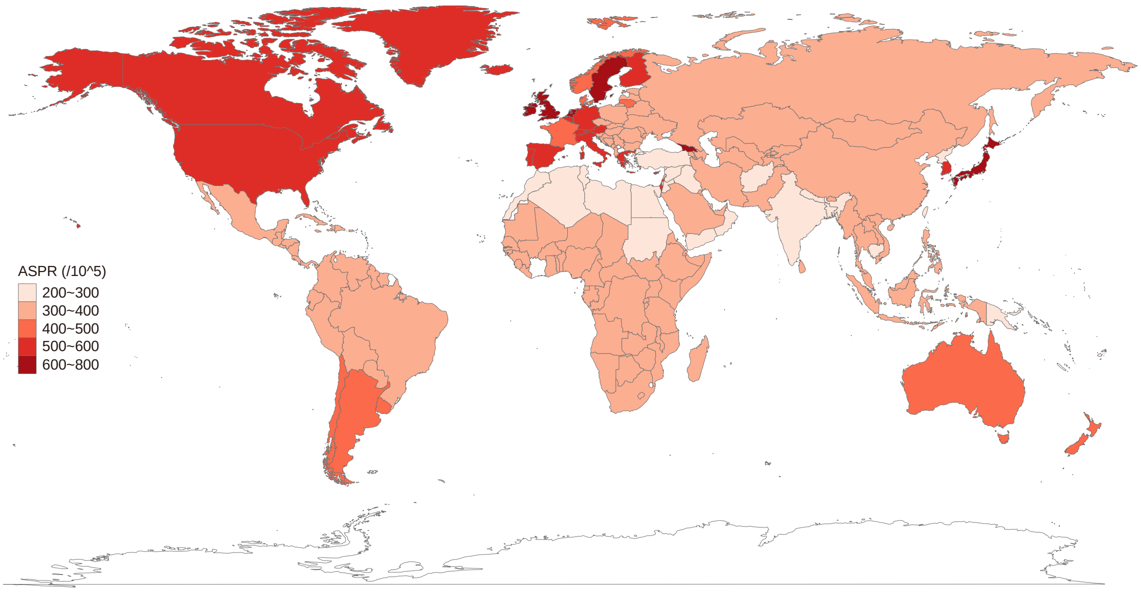

eFigure 3. Global incidence of autism spectrum disorders (ASD) in 1990.

(A) Incident cases of ASD by location for both sexes in 1990.

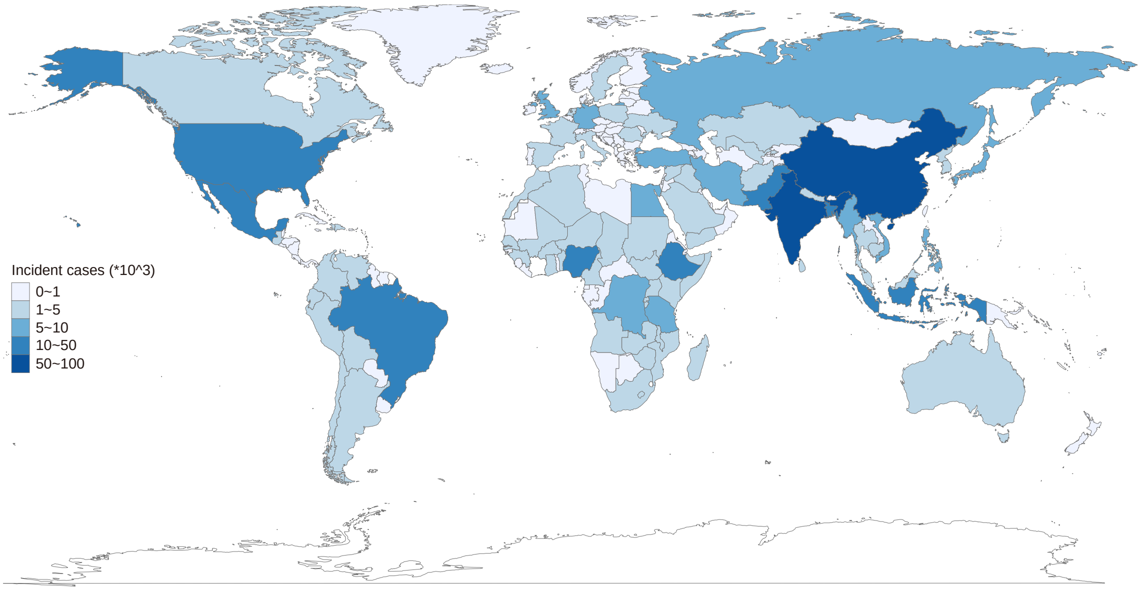

(B) Age-standardized incidence rate (ASIR) of ASD by location for both sexes in 1990.

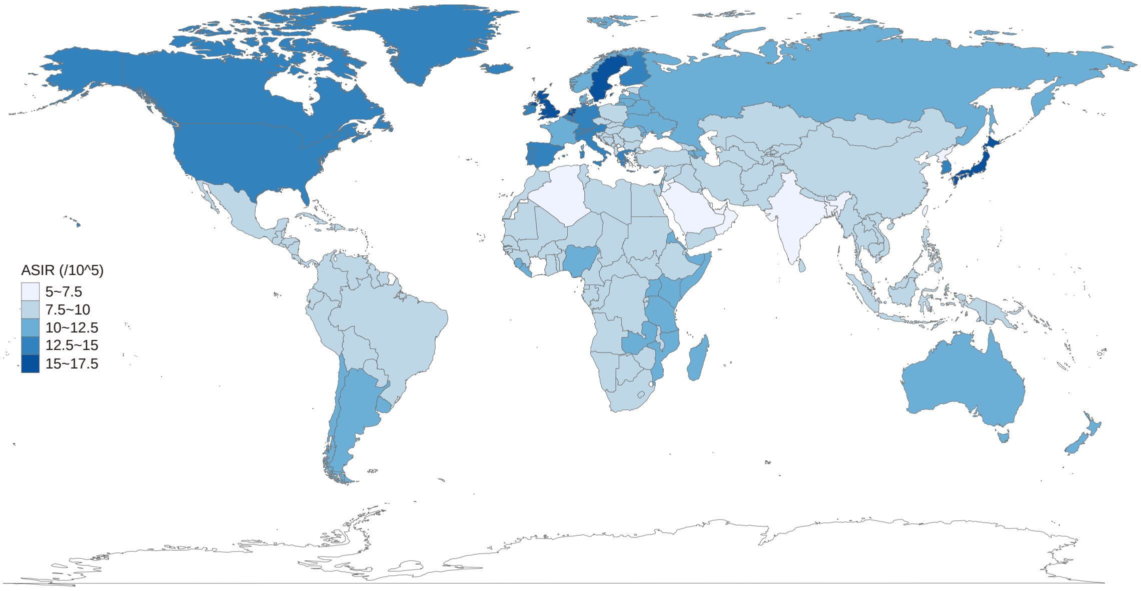

eFigure 4. Global disability-adjusted life-year (DALY) of autism spectrum disorders (ASD) in 1990.

(A) DALYs of ASD by location for both sexes in 1990.

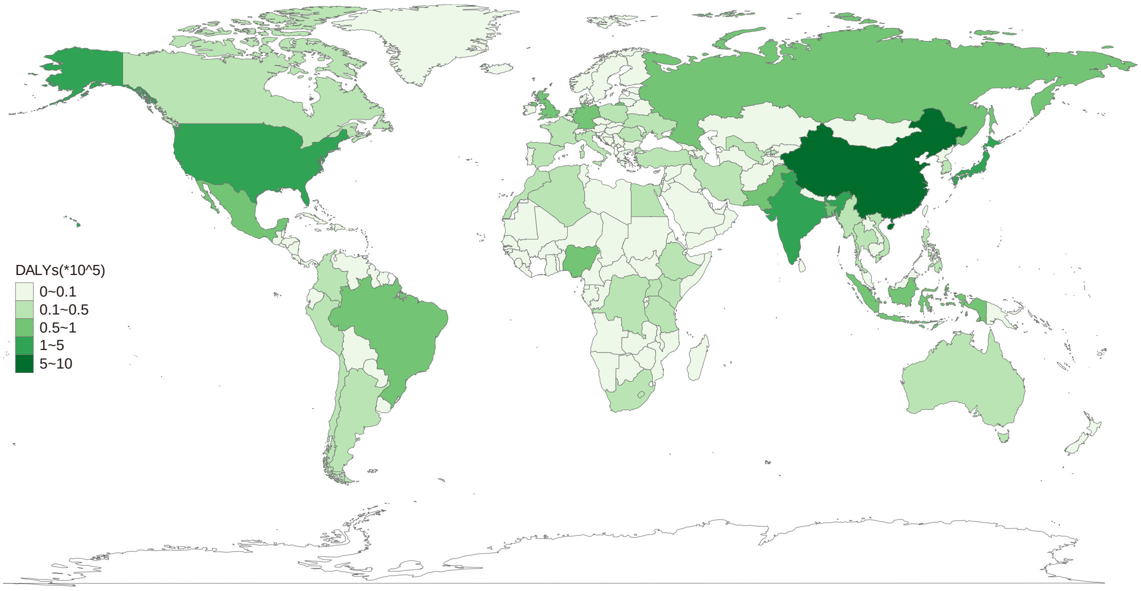

(B) Age-standardized DALY rate of ASD by location for both sexes in 1990.

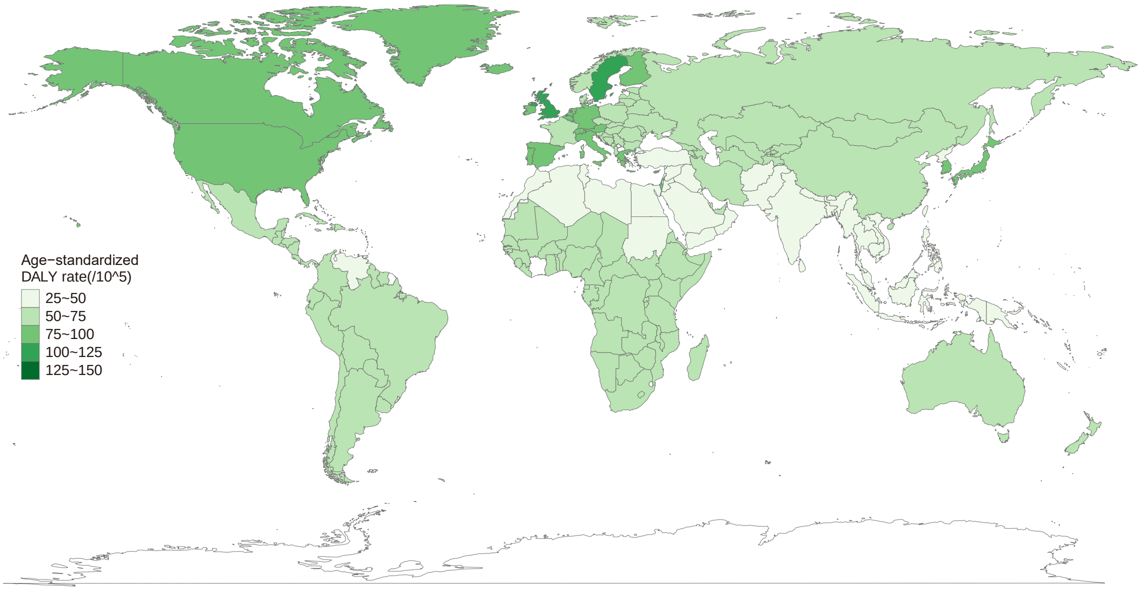

eFigure 5. Global disability-adjusted life-year (DALY) of autism spectrum disorders (ASD) by sex and sociodemographic index (SDI) quintiles from 1990 to 2019.

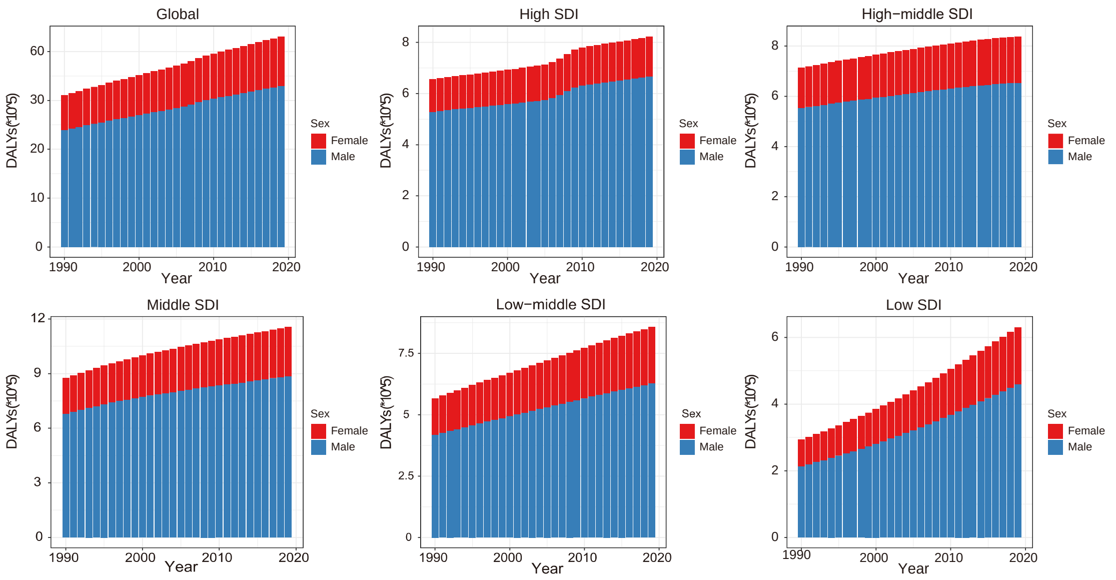

eFigure 6. The correlation analyses of EAPCs-ASR (1990). (A) The correlation between EAPC of ASPR and ASPR of 1990 by location for both sexes. (B) The correlation between EAPC of ASIR and ASIR of 1990 by location for both sexes. (C) The correlation between EAPC of age-standardized DALY rate and ASDR of 1990 by location for both sexes. Note: ASD, autism spectrum disorders; ASPR, age-standardized prevalence rate; ASIR, age-standardized incidence rate; DALY, disability-adjusted life year; EAPC, estimated annual percentage change; SDI, sociodemographic index.

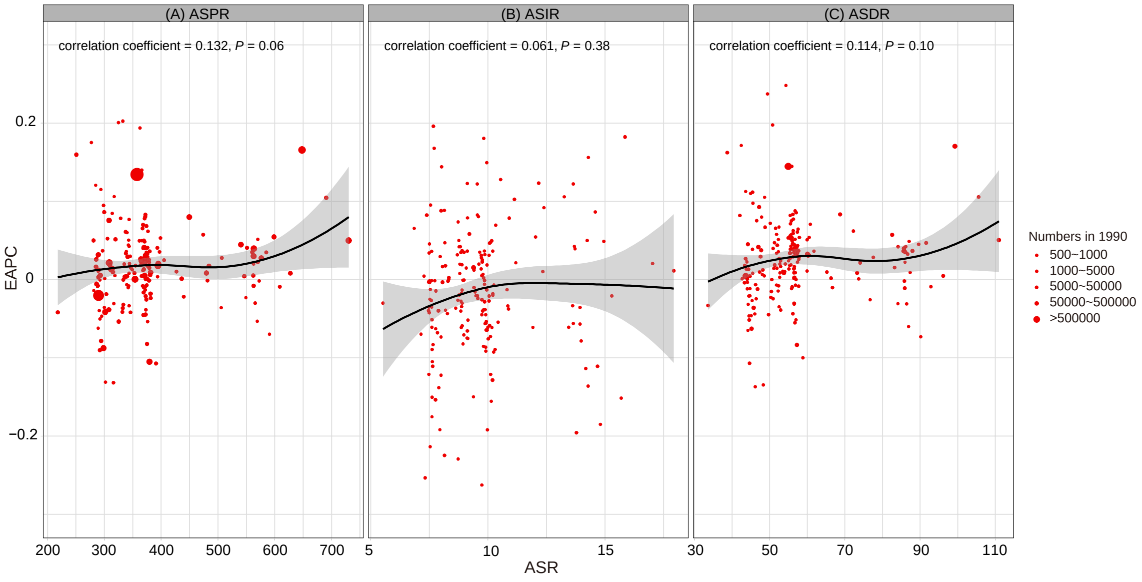

eFigure 7. The correlation analyses of EAPCs-SDI (2019). (A) The correlation between EAPC of ASPR and SDI of 2019 by location for both sexes. (B) The correlation between EAPC of ASIR and SDI of 2019 by location for both sexes. (C) The correlation between EAPC of age-standardized DALY rate and SDI of 2017 by location for both sexes. The size of circle represents the quantity of ASD patients in one country or territory. Note: ASD, autism spectrum disorders; ASPR, age-standardized prevalence rate; ASIR, age-standardized incidence rate; DALY, disability-adjusted life year; EAPC, estimated annual percentage change; SDI, sociodemographic index.

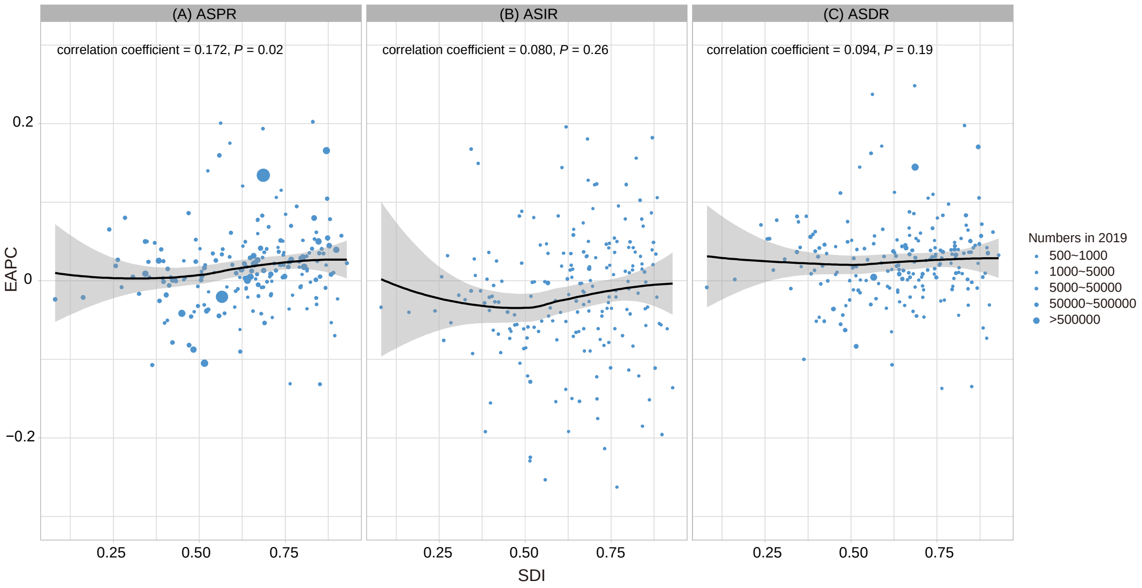

eFigure 8. The change trends and correlation analyses of age-standardized prevalence rate (ASPR) and sociodemographic index (SDI) in 21 regions from 1990 to 2019.

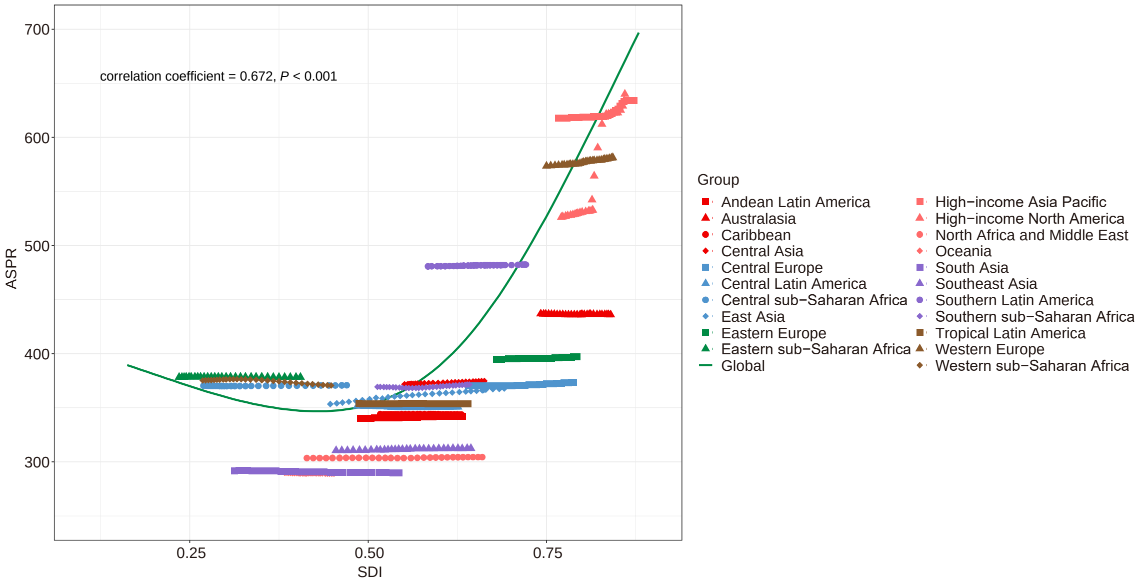

eFigure 9. The change trends and correlation analyses of age-standardized incidence rate (ASIR) and sociodemographic index (SDI) in 21 regions from 1990 to 2019.

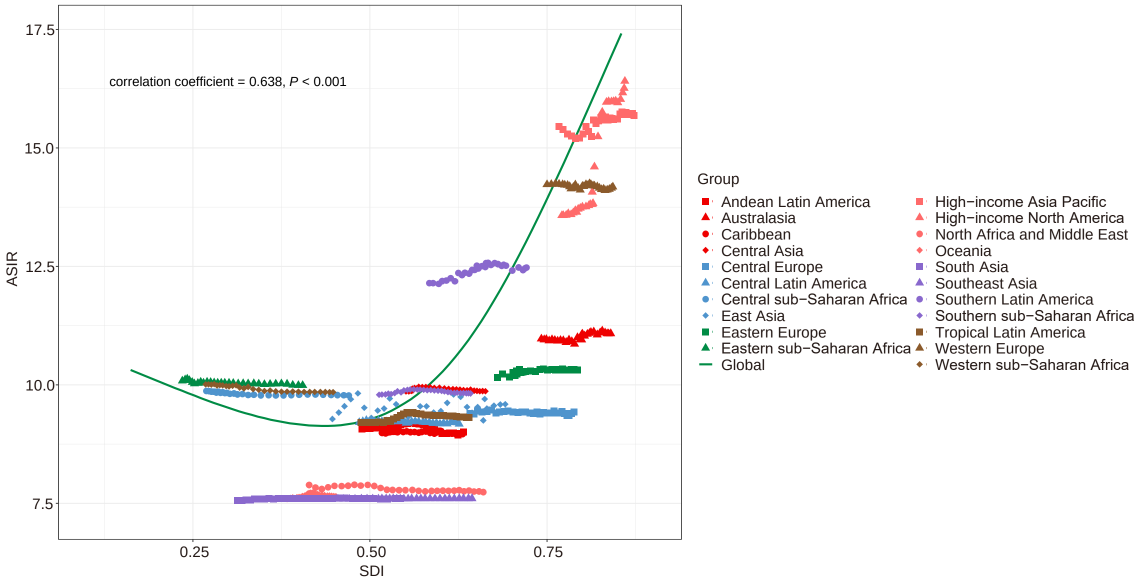

eFigure 10. The change trends and correlation of age-standardized disability-adjusted life year (DALY) rate and sociodemographic index (SDI) in 21 regions from 1990 to 2019.

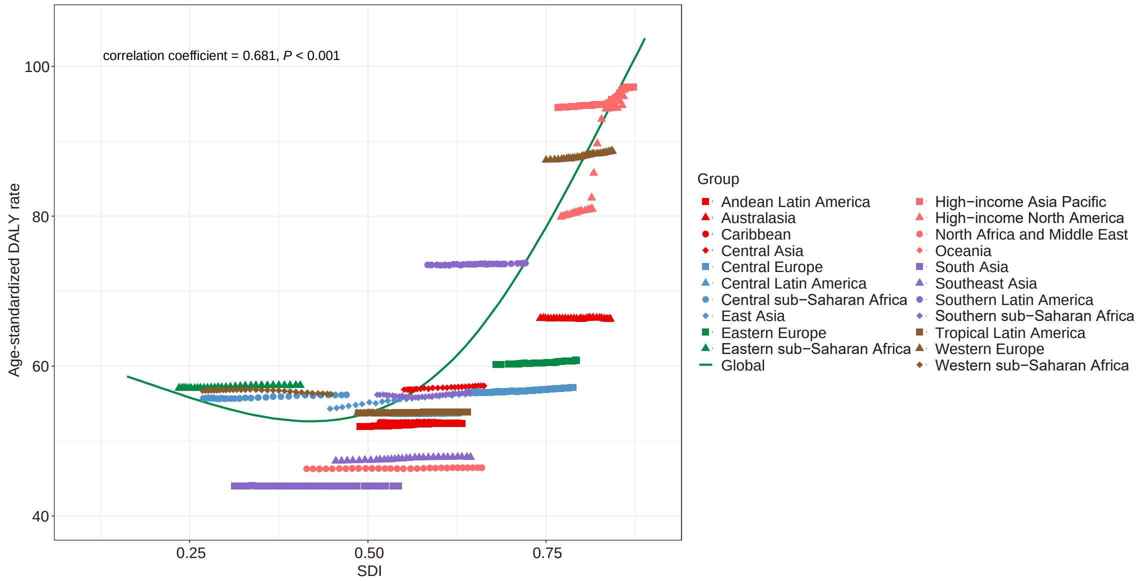

eTable 1. Number and age-standardized prevalence rate for ASD globally and by SDI group, GBD region and super-region, country, and territory in 1990 and 2019.

	Prevalent cases No. (95% UI)			Age-standardized Prevalent rate/100,000 No. (95% UI)			
	**1990**	**2019**	**Change, %**	**1990**	**2019**	**Change, %**	**EAPC No. (95% CI)**
**Global**	20336256 (16857367-24222582)	28324939 (23500644-33811271)	39.3	372.8 (309.1-444.9)	369.4 (305.9-441.2)	-0.9	-0.02 (-0.03 to -0.01)
High SDI	4319311 (3613076-5102134)	5484192 (4587041-6520432)	27.0	539.6 (451.7-638.4)	579.3 (485.3-684.5)	7.4	0.3 (0.25 to 0.35)
High-middle SDI	4665416 (3863289-5575606)	5520271 (4566936-6619212)	18.3	404.6 (334.9-483.3)	405.4 (335.9-485)	0.2	0.01 (0.01 to 0.01)
Middle SDI	5698916 (4667746-6901112)	7566752 (6220224-9144158)	32.8	319.7 (262.7-387.2)	321.4 (264.5-388)	0.5	0.01 (0 to 0.02)
Low-middle SDI	3710774 (3057599-4450285)	5612896 (4621359-6739732)	51.3	311.5 (257.2-373.9)	312.8 (258.2-375.7)	0.4	0.01 (0.01 to 0.01)
Low SDI	1931430 (1592707-2329440)	4125396 (3401890-4965501)	113.6	340.5 (280.2-408.3)	342.2 (281.4-410.2)	0.5	0.01 (0.01 to 0.02)
**High-income North America**	1435440 (1210088-1685566)	2185918 (1833599-2587355)	52.3	526.2 (443.4-617.9)	640 (537.7-756.4)	21.6	0.83 (0.69 to 0.96)
Canada	159035 (137865-183541)	206648 (178807-238417)	29.9	598.7 (519.3-689.9)	608.1 (526.8-696.6)	1.6	0.02 (0.02 to 0.03)
Greenland	287 (236-343)	271 (223-324)	-5.6	505.9 (415.4-604.3)	499.1 (409.9-596.5)	-1.3	-0.04 (-0.04 to -0.03)
United States of	1276085	1978964	55.1	518.5	643.4	24.1	0.91 (0.76 to

America	(1064276-1508544)	(1648438-2352589)		(432-612.8)	(535.1-763.6)		1.07)
**Australasia**	86986 (72543-103962)	119973 (100247-143664)	37.9	436.8 (364.3-521.8)	436.1 (363.8-521.2)	-0.2	0 (0 to 0)
Australia	72180 (60086-86864)	101374 (84293-121654)	40.4	436.2 (363.2-524.1)	435.9 (362.9-523.7)	-0.1	0 (0 to 0)
New Zealand	14806 (12205-17728)	18600 (15367-22296)	25.6	439.9 (362.4-527.4)	437.3 (360.3-524.2)	-0.6	-0.02 (-0.03 to -0.02)
**High-income Asia Pacific**	1047349 (872107-1244347)	1078242 (895833-1288544)	2.9	617.8 (515-734.7)	634.3 (528.8-756.7)	2.7	0.1 (0.08 to 0.12)
Brunei Darussalam	1558 (1285-1877)	2500 (2060-3017)	60.5	572.6 (471.7-689.7)	569.6 (469.2-686.2)	-0.5	0 (-0.03 to 0.02)
Japan	783447 (652062-930475)	772757 (641073-924194)	-1.4	648.1 (540.3-769.3)	676.5 (562.8-805)	4.4	0.17 (0.14 to 0.19)
Singapore	17923 (14779-21666)	31851 (26214-38413)	77.7	585.1 (483.1-704.6)	588.1 (485.4-707.6)	0.5	0.03 (0.01 to 0.06)
Republic of Korea	244421 (201800-291106)	271135 (224314-324212)	10.9	540.4 (445.7-643.7)	548.9 (452.7-653.7)	1.6	0.04 (0.04 to 0.05)
**Western Europe**	2113126 (1773298-2507729)	2354684 (1967645-2791090)	11.4	573.7 (482.7-679.9)	581.3 (488.2-686.4)	1.3	0.05 (0.04 to 0.05)
Andorra	316 (258-381)	454 (371-550)	44.0	590.6 (483.8-711.7)	580.4 (476.1-699.5)	-1.7	-0.07 (-0.08 to -0.06)
Austria	41437 (34168-49456)	46901 (38621-55879)	13.2	560.3 (462.3-667.4)	567.5 (468.3-675.3)	1.3	0.04 (0.03 to 0.04)
Belgium	53520 (44050-64515)	60079 (49394-72457)	12.3	562.1 (463.8-675.7)	563.3 (464.7-677.1)	0.2	0.01 (0 to 0.01)
Cyprus	4365 (3617-5221)	7032 (5821-8458)	61.1	565.6 (468.6-675.9)	562.3 (465.8-672)	-0.6	-0.03 (-0.03 to -0.03)

Denmark	21030 (20455-21866)	23434 (22774-24341)	11.4	427.2 (415.7-444.2)	429 (417.9-444.9)	0.4	0.01 (0.01 to 0.01)
Finland	26433 (21828-31601)	28483 (23521-34106)	7.8	551.2 (455.4-658.4)	557.7 (460.9-666.1)	1.2	0.04 (0.04 to 0.04)
France	250128 (212134-301146)	281641 (240099-328886)	12.6	449.5 (381.8-540.3)	457.1 (390-533.8)	1.7	0.08 (0.07 to 0.09)
Germany	425508 (347300-511045)	446061 (364617-535795)	4.8	563.1 (460.3-676.6)	572.3 (467.4-687.2)	1.6	0.04 (0.03 to 0.05)
Greece	56206 (46638-68031)	53309 (44143-64550)	-5.2	562.6 (467.1-679.9)	562.3 (466.9-679.7)	-0.1	-0.01 (-0.02 to 0)
Iceland	1417 (1179-1685)	1874 (1557-2233)	32.2	562.8 (468.2-669.2)	566.1 (471.1-673.2)	0.6	0.02 (0.01 to 0.03)
Ireland	21794 (17951-26109)	28661 (23570-34412)	31.5	608.9 (501.8-729.1)	607.3 (500.5-727.2)	-0.3	-0.01 (-0.02 to 0)
Israel	25344 (21066-30277)	46966 (39011-56164)	85.3	506.7 (420.8-605.6)	510.4 (423.9-610)	0.7	0.03 (0.03 to 0.03)
Italy	303941 (252029-361920)	311271 (257191-371643)	2.4	562.8 (467.4-668.6)	567.9 (471.2-675.1)	0.9	0.03 (0.03 to 0.03)
Luxembourg	2062 (1698-2484)	3350 (2755-4039)	62.5	565.2 (465.3-679.9)	571.4 (470.4-687.5)	1.1	0.04 (0.03 to 0.04)
Malta	2076 (1701-2485)	2363 (1926-2842)	13.8	570 (467.2-682.2)	578.4 (473.9-692.3)	1.5	0.05 (0.05 to 0.05)
Netherlands	90797 (74254-108939)	101490 (82817-121694)	11.8	627.5 (513.6-751.9)	629.6 (515.2-754.7)	0.3	0.01 (0.01 to 0.01)
Norway	19245 (15890-23055)	24492 (20161-29291)	27.3	473.8 (392.2-567.2)	484.4 (399.4-577.4)	2.2	0.06 (0.05 to 0.07)
Portugal	53485 (43883-64100)	52895 (43368-63382)	-1.1	546.3	547.8	0.3	0 (0 to 0.01)

				(448.2-653.2)	(449.4-654.9)		
Spain	216156 (178352-261299)	246279 (203164-298473)	13.9	576.4 (476.6-698.1)	579.5 (479.2-701.5)	0.5	0.03 (0.02 to 0.04)
Sweden	56224 (46912-66743)	67658 (56338-80572)	20.3	690.7 (576.1-818.3)	706.8 (589.1-838.5)	2.3	0.1 (0.1 to 0.11)
Switzerland	37401 (30743-45100)	46985 (38566-56702)	25.6	570 (468-684.3)	573.4 (470.7-688.4)	0.6	0.02 (0.01 to 0.03)
United Kingdom	402206 (334662-476122)	470592 (391239-557167)	17.0	730.3 (609.3-865.4)	739.6 (617.2-876.3)	1.3	0.05 (0.05 to 0.05)
**Southern Latin America**	240321 (199640-288082)	313298 (260284-376359)	30.4	480.8 (399.3-576.6)	482.5 (400.8-579)	0.4	0.01 (0.01 to 0.01)
Argentina	159816 (131719-192285)	212154 (174262-255669)	32.7	479.5 (394.9-577.2)	480.5 (395.8-578.4)	0.2	0.01 (0.01 to 0.01)
Chile	65647 (54771-78632)	85390 (70985-102813)	30.1	484.1 (403.9-581.1)	487.6 (406.8-585.1)	0.7	0.07 (0.06 to 0.07)
Uruguay	14848 (12244-17879)	15738 (12987-18965)	6.0	481.5 (396.9-580)	481.4 (396.8-579.9)	0.0	0 (0 to 0)
**Eastern Europe**	866526 (716655-1038602)	773320 (638888-927709)	-10.8	394.8 (326.1-472.8)	397.3 (328.3-476)	0.6	0.02 (0.02 to 0.02)
Belarus	40317 (33163-48418)	35148 (28877-42260)	-12.8	396.7 (326.7-476)	399.6 (329.1-479.7)	0.7	0.02 (0.02 to 0.02)
Estonia	6031 (4952-7269)	4849 (3992-5856)	-19.6	398.7 (327.6-479.3)	404.1 (331.8-485.6)	1.4	0.05 (0.05 to 0.06)
Latvia	10121 (8346-12162)	6943 (5734-8367)	-31.4	397 (328-475.9)	401 (331.4-480.9)	1.0	0.04 (0.03 to 0.04)
Lithuania	14465 (11939-17371)	10320 (8542-12425)	-28.7	405.1 (334.5-486.4)	407.8 (336.7-489.8)	0.7	0.02 (0.02 to 0.03)

Republic of Moldova	17444 (14371-21047)	13678 (11262-16517)	-21.6	393.2 (324-474.5)	397.8 (327.8-480.1)	1.2	0.04 (0.04 to 0.04)
Russian Federation	578995 (477809-692806)	541938 (445878-649451)	-6.4	394.2 (325.1-471.1)	396.8 (327-474.3)	0.7	0.02 (0.02 to 0.02)
Ukraine	199152 (164228-240709)	160444 (132414-194047)	-19.4	395 (325.8-478.2)	397.6 (327.9-481.2)	0.7	0.02 (0.02 to 0.02)
**Central Europe**	445113 (367189-532928)	393541 (325032-472445)	-11.6	370.5 (305.8-443.3)	373.6 (308.4-446.9)	0.8	0.03 (0.03 to 0.03)
Albania	12735 (10409-15355)	9569 (7816-11567)	-24.9	372.8 (305.4-450.4)	370.7 (303.6-447.7)	-0.6	-0.01 (-0.03 to 0.01)
Bosnia and Herzegovina	17190 (14030-20507)	11605 (9486-13871)	-32.5	378.7 (308.7-451.3)	378.6 (308.6-451.2)	0.0	0.02 (0.01 to 0.03)
Bulgaria	30584 (25052-36891)	23271 (19071-28093)	-23.9	368.3 (301.8-444.8)	370.3 (303.4-447.2)	0.5	0.02 (0.02 to 0.03)
Croatia	17750 (14472-21407)	14731 (12002-17801)	-17.0	375.9 (306.8-453.3)	378.6 (309.1-456.5)	0.7	0.03 (0.03 to 0.03)
Czechia	37349 (30385-45341)	37343 (30422-45457)	0.0	375.6 (305-455.6)	379.7 (308.4-460.6)	1.1	0.04 (0.04 to 0.04)
Hungary	36905 (30101-44569)	32923 (26742-39867)	-10.8	371.4 (303.9-448.7)	373.7 (305.8-451.4)	0.6	0.02 (0.02 to 0.03)
North Macedonia	7579 (6209-9125)	7756 (6357-9350)	2.3	376 (308.1-452.4)	378.9 (310.4-456)	0.8	0.03 (0.03 to 0.04)
Montenegro	2341 (1913-2844)	2211 (1804-2691)	-5.5	375.6 (306.7-456.2)	377.4 (308.3-458.4)	0.5	0.02 (0.01 to 0.02)
Poland	139026 (113838-166355)	132648 (108511-158839)	-4.6	371.1 (304.4-443.9)	373.9 (306.6-447.3)	0.8	0.03 (0.03 to 0.03)
Romania	83328 (68094-99597)	64616 (52833-77194)	-22.5	364.2	366.4	0.6	0.02 (0.02 to

				(297.8-435.2)	(299.7-437.8)		0.03)
Serbia	33778 (27755-40676)	30593 (25051-36829)	-9.4	368.2 (301.8-443)	375.4 (307.5-451.8)	2.0	0.07 (0.06 to 0.08)
Slovakia	19345 (15894-23326)	18986 (15576-22972)	-1.9	371.6 (304.8-447.8)	374.6 (307.4-451.2)	0.8	0.03 (0.03 to 0.03)
Slovenia	7203 (5861-8689)	7288 (5927-8847)	1.2	376.7 (306.5-454.6)	382.7 (311.5-461.9)	1.6	0.06 (0.06 to 0.06)
**Central Asia**	268361 (220526-321320)	354787 (291432-426875)	32.2	371.7 (305.9-446.9)	374.8 (308-450.9)	0.8	-0.01 (-0.02 to 0)
Armenia	13214 (10782-15982)	11051 (9025-13411)	-16.4	379.9 (310-459.1)	383.1 (312.5-462.8)	0.8	0.04 (0.03 to 0.04)
Azerbaijan	28268 (23283-33950)	38492 (31577-46355)	36.2	372.2 (306.3-447.8)	379.8 (312.2-456.6)	2.0	0.08 (0.08 to 0.09)
Georgia	20312 (16764-24315)	13085 (10782-15660)	-35.6	374.3 (309.3-447.9)	380.8 (314.6-455.9)	1.7	0.23 (-0.6 to 1.07)
Kazakhstan	62025 (50987-74754)	68096 (55993-82158)	9.8	369.4 (303.8-445.8)	371 (305.1-447.5)	0.4	0.01 (0.01 to 0.02)
Kyrgyzstan	17298 (14149-20762)	25055 (20541-30100)	44.8	370.8 (304.2-446)	373.4 (306.5-449)	0.7	0.02 (0.02 to 0.02)
Mongolia	8537 (6964-10288)	12773 (10410-15404)	49.6	372 (303.3-448.5)	370.4 (301.9-446.4)	-0.4	-0.01 (-0.02 to -0.01)
Tajikistan	21408 (17670-25832)	37244 (30699-45012)	74.0	374 (307.3-451.3)	377.3 (309.9-455.1)	0.9	0.03 (0.03 to 0.04)
Turkmenistan	14627 (11979-17596)	19570 (16061-23615)	33.8	370.8 (304.3-447.7)	378.8 (310.7-457.4)	2.2	0.08 (0.08 to 0.08)
Uzbekistan	82672 (67302-100061)	129421 (105487-156624)	56.5	371.8 (302.7-450)	374.2 (304.7-453)	0.6	0.02 (0.02 to 0.02)

**Central Latin America**	608677 (502158-726427)	875348 (720122-1047034)	43.8	351.7 (289.6-420.6)	350.9 (288.8-419.5)	-0.2	0.03 (0.03 to 0.04)
Colombia	114322 (92774-138003)	157698 (127974-191011)	37.9	336.1 (272.8-407)	336.2 (272.8-407)	0.0	0.13 (0.13 to 0.14)
Costa Rica	10954 (8909-13210)	15855 (12920-19155)	44.7	346 (282.2-417.7)	342.5 (279.2-413.5)	-1.0	0.04 (0.04 to 0.04)
El Salvador	18519 (15065-22377)	20617 (16730-24970)	11.3	331.8 (269.3-401.6)	327.9 (266-396.9)	-1.2	-0.04 (-0.05 to -0.04)
Guatemala	28425 (23307-33974)	60922 (49883-72922)	114.3	332.4 (271.6-397.7)	329 (268.8-393.5)	-1.0	-0.04 (-0.04 to -0.03)
Honduras	16928 (13765-20452)	33751 (27538-40717)	99.4	333.6 (272.7-402.1)	330.9 (270.6-398.8)	-0.8	-0.03 (-0.04 to -0.03)
Mexico	334563 (275855-398323)	462735 (381484-554085)	38.3	371 (306-443.6)	371.6 (306.3-444.9)	0.2	0.01 (0 to 0.01)
Nicaragua	14049 (11455-16835)	22505 (18391-27014)	60.2	335 (273.5-401.9)	336.9 (275.2-404.2)	0.6	0.02 (0.02 to 0.02)
Panama	8476 (6863-10164)	14208 (11515-17068)	67.6	342.7 (277.8-411.6)	342.2 (277.4-411)	-0.1	-0.01 (-0.01 to -0.01)
Venezuela (Bolivarian Republic of)	62442 (51293-75524)	87057 (71536-105438)	39.4	315.2 (259.3-381.3)	314.3 (258.5-380.3)	-0.3	0.01 (0 to 0.02)
**Andean Latin America**	136708 (112528-164539)	219156 (180811-263070)	60.3	340.4 (280.9-408.5)	342.1 (282.4-410.5)	0.5	0.02 (0.02 to 0.02)
Bolivia (Plurinational State of)	23071 (18860-27930)	41997 (34422-50775)	82.0	337.7 (277-408.4)	340.8 (279.6-412.1)	0.9	0.03 (0.03 to 0.04)
Ecuador	35854 (29385-43179)	60491 (49658-72945)	68.7	340 (279-410.4)	340.5 (279.4-411.1)	0.1	0 (0 to 0.01)
Peru	77783 (63705-93582)	116668 (95667-140645)	50.0	341.2	343.4	0.6	0.03 (0.03 to

				(279.7-411.1)	(281.5-413.7)		0.03)
**Caribbean**	124630 (102831-149922)	159960 (132108-192303)	28.3	344.2 (284-414.1)	343.8 (283.7-413.6)	-0.1	0.05 (0.04 to 0.07)
Antigua and Barbuda	212 (173-256)	297 (243-359)	40.1	342.7 (280.3-414.1)	346.1 (283.1-418.2)	1.0	0.05 (0.03 to 0.07)
Bahamas	902 (740-1094)	1250 (1029-1516)	38.5	339.3 (279.6-411.7)	339.1 (279.5-411.5)	-0.1	-0.01 (-0.01 to 0)
Barbados	866 (716-1040)	966 (797-1168)	11.5	344.3 (284.4-413.3)	346.8 (286.5-416.1)	0.7	0.02 (0.02 to 0.02)
Belize	678 (556-816)	1427 (1168-1715)	110.4	343.6 (281.2-413.2)	340.6 (278.7-409.7)	-0.9	-0.03 (-0.04 to -0.03)
Bermuda	204 (165-246)	206 (167-248)	1.0	349.1 (283.5-421.3)	350.4 (284.5-422.9)	0.4	0.01 (0 to 0.02)
Cuba	38184 (30996-46289)	37585 (30583-45647)	-1.6	352.9 (286.5-428.1)	353.7 (287.2-429.1)	0.2	0.01 (0.01 to 0.01)
Dominica	260 (212-313)	231 (189-278)	-11.0	343.4 (279.8-413.3)	347.3 (283.3-417.8)	1.1	0.04 (0.04 to 0.04)
Dominican Republic	25579 (20782-30823)	37535 (30457-45299)	46.7	337.5 (274-407.5)	342 (277.6-412.7)	1.3	0.06 (0.05 to 0.07)
Grenada	305 (248-368)	356 (289-430)	16.7	343.5 (279.7-415.2)	350.6 (285.5-424)	2.1	0.08 (0.07 to 0.09)
Guyana	2742 (2252-3306)	2637 (2170-3181)	-3.8	338 (278.4-408.1)	338.6 (278.9-408.7)	0.2	0 (0 to 0.01)
Haiti	22542 (18454-27022)	43111 (35312-51694)	91.3	333.6 (273-400.1)	333.8 (273.1-400.3)	0.1	0 (0 to 0)
Jamaica	8436 (6918-10181)	9649 (7926-11614)	14.4	345.5 (283.5-415.7)	348.3 (285.8-419.2)	0.8	0.03 (0.03 to 0.03)

Puerto Rico	12526 (10242-15081)	11247 (9190-13608)	-10.2	346.7 (283.6-417.4)	347.8 (284.4-418.7)	0.3	0.01 (0.01 to 0.02)
Saint Lucia	490 (400-589)	581 (474-700)	18.6	339.9 (276.9-408.8)	345.4 (281.7-415.5)	1.6	0.06 (0.06 to 0.06)
Saint Vincent and the Grenadines	395 (321-475)	383 (311-461)	-3.0	342.4 (277.9-412.2)	347.1 (281.4-417.7)	1.4	0.04 (0.04 to 0.05)
Suriname	1370 (1128-1659)	1939 (1596-2348)	41.5	342 (281.3-414.1)	340.7 (280.2-412.6)	-0.4	-0.01 (-0.01 to -0.01)
Trinidad and Tobago	4276 (3486-5177)	4611 (3760-5611)	7.8	345 (281.8-418.1)	346.7 (283.2-420.2)	0.5	0.02 (0.02 to 0.02)
United States Virgin Islands	368 (303-444)	334 (274-403)	-9.3	343.5 (282.6-414.1)	343.9 (282.9-414.6)	0.1	0.01 (0 to 0.02)
**Tropical Latin America**	563007 (465390-674324)	774446 (638700-930842)	37.6	353.9 (292-425.4)	353.9 (292-425.3)	0.0	0 (0 to 0)
Brazil	547893 (452381-656204)	749431 (618422-900974)	36.8	353.9 (292-425.4)	353.8 (291.9-425.2)	0.0	0 (0 to 0)
Paraguay	15114 (12353-18237)	25014 (20465-30198)	65.5	354.9 (290.3-428.7)	356.5 (291.7-430.6)	0.5	0.02 (0.02 to 0.02)
**East Asia**	4412839 (3624038-5309093)	5098806 (4206229-6150048)	15.5	353.5 (290.3-425.4)	367.8 (304.4-441.9)	4.0	0.13 (0.12 to 0.14)
China	4313669 (3540479-5189318)	4985784 (4111846-6016905)	15.6	357.7 (293.6-430.7)	372.3 (307.5-447.6)	4.1	0.02 (0.01 to 0.02)
Democratic People's Republic of Korea	54195 (44014-65478)	66019 (53571-79704)	21.8	250.8 (203.4-302.9)	261.4 (212.2-315.9)	4.2	0.16 (0.15 to 0.16)
Taiwan (province of China)	44975 (38172-53467)	47004 (39746-55779)	4.5	218.2 (185.2-259.1)	215.8 (183.2-256.4)	-1.1	-0.04 (-0.04 to -0.04)
**Southeast Asia**	1519341	2097143	38.0	310.5	312.5	0.6	0.02 (0.02 to

	(1253044-1824857)	(1729612-2515054)		(256.1-373.2)	(257.7-374.4)		0.03)
Cambodia	33866 (27652-41230)	52300 (42620-63668)	54.4	299.7 (244.8-366)	307.1 (250.9-375.2)	2.5	0.14 (0.14 to 0.14)
Indonesia	598898 (493295-717466)	804573 (662785-963425)	34.3	308.7 (254.7-370.1)	310.3 (255.9-371.4)	0.5	0.02 (0.02 to 0.02)
Lao People's Democratic Republic	13577 (11165-16389)	22914 (18842-27688)	68.8	305.4 (250.8-369.2)	310.2 (254.6-375.1)	1.6	0.05 (0.05 to 0.06)
Malaysia	58963 (48156-70729)	101613 (83064-122070)	72.3	319.6 (261-383.9)	324 (264.7-389.3)	1.4	0.05 (0.05 to 0.05)
Maldives	761 (618-919)	1761 (1431-2130)	131.5	324.9 (264.3-393.1)	343.9 (279.4-415.9)	5.8	0.2 (0.14 to 0.26)
Mauritius	3600 (2956-4333)	3886 (3186-4688)	8.0	318.7 (261.7-383.1)	319.1 (262-383.6)	0.1	0.01 (0 to 0.01)
Myanmar	133365 (108348-160377)	167622 (136792-201441)	25.7	307.9 (251.2-369.7)	304.7 (248.6-365.9)	-1.0	-0.04 (-0.04 to -0.04)
Philippines	210504 (173169-252631)	363296 (299490-436981)	72.6	313.9 (258.7-377.4)	315.4 (259.9-379.3)	0.5	0.01 (0.01 to 0.02)
Sri Lanka	57641 (47138-69538)	68402 (56011-82745)	18.7	325.4 (266.3-393.4)	320.6 (262.3-387.2)	-1.5	-0.05 (-0.06 to -0.05)
Seychelles	238 (194-285)	325 (265-390)	36.8	317.2 (258.6-380.5)	325 (265.2-389.8)	2.5	0.11 (0.09 to 0.12)
Thailand	182157 (148520-219506)	204477 (166247-246494)	12.3	311.2 (253.3-375.6)	312.1 (254-376.7)	0.3	0.01 (0.01 to 0.02)
Timor-Leste	2647 (2163-3192)	4381 (3587-5284)	65.5	316.8 (260-381.7)	313.5 (257.3-377.9)	-1.0	-0.03 (-0.04 to -0.02)
Viet Nam	221106 (180172-267112)	298844 (244316-362011)	35.2	308.3 (252.3-372.7)	314.9 (257.8-380.6)	2.1	0.08 (0.07 to 0.08)

**Oceania**	19903 (16274-23989)	40114 (32728-48523)	101.5	290 (236.8-350.4)	289 (235.5-349)	-0.3	-0.01 (-0.01 to 0)
American Samoa	150 (123-183)	164 (134-199)	9.1	295.2 (241.5-359)	291.8 (238.6-354.9)	-1.2	-0.05 (-0.05 to -0.04)
Micronesia (Federated States of)	323 (261-390)	302 (243-364)	-6.6	289.4 (234.1-349)	288 (232.8-347.4)	-0.5	-0.01 (-0.02 to 0)
Fiji	2305 (1903-2762)	2665 (2204-3199)	15.6	289.3 (239.4-347.4)	289.2 (239.3-347.3)	0.0	0 (0 to 0.01)
Guam	424 (345-516)	500 (407-609)	17.9	300.1 (244-365.2)	296.3 (240.7-360.6)	-1.3	-0.04 (-0.06 to -0.01)
Kiribati	222 (181-267)	348 (283-419)	56.7	281.5 (230-338.9)	279.9 (228.7-337)	-0.6	-0.01 (-0.02 to -0.01)
Marshall Islands	143 (117-173)	169 (138-204)	18.3	288.9 (236.4-350.4)	289.4 (236.8-351.1)	0.2	0.01 (0.01 to 0.01)
Northern Mariana Islands	141 (114-170)	123 (99-148)	-12.8	303.1 (245.7-366.1)	300.1 (243.3-362.3)	-1.0	-0.02 (-0.07 to 0.03)
Papua New Guinea	12574 (10206-15269)	29944 (24310-36372)	138.1	289.2 (235.3-351.9)	289 (235.1-351.6)	-0.1	0 (0 to 0.01)
Samoa	513 (419-620)	641 (523-773)	25.0	296 (241-357)	294 (239.4-354.8)	-0.7	-0.02 (-0.02 to -0.02)
Solomon Islands	1065 (874-1290)	1996 (1639-2418)	87.4	292.2 (240-353.2)	288.4 (236.9-348.6)	-1.3	-0.05 (-0.05 to -0.05)
Tonga	295 (240-357)	303 (247-366)	2.7	287.8 (234-348.5)	287.2 (233.4-347.7)	-0.2	-0.01 (-0.02 to 0)
Vanuatu	469 (380-566)	884 (718-1068)	88.7	290.9 (235.8-350.2)	287.8 (233.2-346.4)	-1.1	-0.04 (-0.04 to -0.04)
**North Africa and**	1105582	1879528	70.0	303.5	304.4	0.3	0.01 (0.01 to

**Middle East**	(911505-1326857)	(1550850-2261649)		(250.4-365.5)	(251.2-366.1)		0.01)
Afghanistan	34339 (27895-41484)	119198 (96727-143892)	247.1	280.9 (228.3-340.4)	287.1 (233.2-347.9)	2.2	0.05 (0.04 to 0.06)
Algeria	77704 (63511-93280)	122382 (100311-147314)	57.5	290 (237.6-349)	290.9 (238.3-350)	0.3	0.01 (0.01 to 0.01)
Bahrain	1668 (1351-2021)	4631 (3740-5630)	177.6	313.8 (253.7-379.9)	316.6 (255.9-382.9)	0.9	0.08 (0.06 to 0.11)
Egypt	169967 (138456-206156)	296910 (242086-359990)	74.7	289.5 (235.8-350.8)	292.2 (238-353.9)	0.9	0.03 (0.03 to 0.04)
Iran (Islamic Republic of)	224822 (185065-268769)	310410 (256980-369841)	38.1	364.3 (299.8-437.5)	370.3 (306.8-441.4)	1.6	0.03 (0.01 to 0.04)
Iraq	54051 (43955-65198)	125447 (101636-151344)	132.1	286.3 (231.5-345.3)	286.8 (231.9-345.9)	0.2	-0.01 (-0.01 to 0)
Jordan	11973 (9798-14566)	35886 (29427-43705)	199.7	296.9 (243.5-360.9)	299.9 (246-364.2)	1.0	0.04 (0.02 to 0.05)
Kuwait	5741 (4670-6974)	13397 (10887-16293)	133.4	315.9 (256.7-383.9)	305 (248-370.1)	-3.5	-0.13 (-0.17 to -0.09)
Lebanon	10333 (8401-12523)	15555 (12634-18884)	50.5	303.7 (246.8-368.6)	302.3 (245.6-367)	-0.5	-0.02 (-0.03 to -0.01)
Libya	13001 (10677-15797)	19233 (15779-23362)	47.9	290.8 (238.9-353.2)	286.5 (235.2-347.9)	-1.5	-0.02 (-0.03 to 0)
Morocco	75984 (62182-91713)	103372 (84720-124834)	36.0	285.6 (234-344.9)	287.5 (235.6-347.2)	0.7	0.03 (0.02 to 0.03)
Oman	6125 (4990-7406)	14594 (11942-17659)	138.3	298.4 (244.2-360.6)	303 (248.2-366)	1.5	0.09 (0.03 to 0.16)
Palestine	6473 (5273-7816)	15181 (12372-18351)	134.5	288.6 (235.1-349.3)	292.1 (237.8-353.6)	1.2	0.03 (0.02 to 0.04)

Qatar	1552 (1275-1865)	10343 (8473-12465)	566.6	332.5 (273.6-400.6)	344.7 (283.2-415.7)	3.7	0.2 (0.16 to 0.25)
Saudi Arabia	52063 (42293-62728)	112394 (91444-136141)	115.9	310 (252.4-374.5)	310.9 (253.2-375.6)	0.3	0.02 (0 to 0.04)
Sudan	61796 (50219-74357)	123282 (100061-148317)	99.5	285.4 (232.8-343)	286.7 (233.9-344.7)	0.5	0.02 (0.01 to 0.02)
Syrian Arab Republic	40283 (32933-48754)	41510 (33817-50467)	3.0	292 (237.9-355.1)	283.9 (231.3-345.4)	-2.8	-0.09 (-0.13 to -0.05)
Tunisia	25671 (21007-30985)	32915 (26833-39903)	28.2	291.8 (238.2-353.8)	290.5 (237.2-352.2)	-0.4	-0.02 (-0.02 to -0.02)
Turkey	181816 (148231-221243)	233196 (190172-283292)	28.3	292.6 (238.9-355)	293.3 (239.4-355.8)	0.2	0.01 (0 to 0.01)
United Arab Emirates	6464 (5300-7796)	31944 (26250-38509)	394.2	328.5 (269.7-397.5)	331.2 (271.9-400.8)	0.8	0.08 (0.04 to 0.11)
Yemen	43014 (35298-51808)	95839 (78745-115683)	122.8	285.9 (234.4-346.8)	286 (234.6-347)	0.0	-0.01 (-0.01 to 0)
**South Asia**	3370030 (2767916-4062245)	5310541 (4355785-6406808)	57.6	291.9 (240.1-351.9)	290 (238.4-349.2)	-0.7	-0.03 (-0.03 to -0.03)
Bangladesh	345633 (282283-413375)	468932 (382537-561058)	35.7	298.4 (243.6-357.2)	292.3 (238.4-349.6)	-2.0	-0.09 (-0.1 to -0.08)
Bhutan	1966 (1606-2376)	2286 (1869-2764)	16.3	300.7 (245.7-363.3)	299.5 (244.7-362)	-0.4	0 (-0.01 to 0.01)
India	2599711 (2124879-3139560)	4046199 (3320489-4881900)	55.6	289.8 (238.3-349.3)	288.5 (237.1-347.9)	-0.4	-0.02 (-0.02 to -0.02)
Nepal	61112 (49728-73633)	89803 (73155-108122)	46.9	294.4 (239.8-354.3)	288.6 (235-347.6)	-2.0	-0.08 (-0.09 to -0.07)
Pakistan	361607 (294461-434003)	703321 (572848-844803)	94.5	301.5	298.1	-1.1	-0.04 (-0.04 to

				(246.8-364)	(244.1-359.3)		-0.04)
**Southern sub-Saharan Africa**	204885 (167918-245329)	298595 (244999-358553)	45.7	369.4 (302.9-444.8)	371.6 (304.9-447.7)	0.6	0.02 (0.02 to 0.03)
Botswana	5115 (4193-6205)	8965 (7321-10894)	75.3	366.2 (298.4-444.2)	372 (303.6-451.4)	1.6	0.05 (0.05 to 0.05)
Lesotho	7075 (5763-8518)	8031 (6523-9687)	13.5	367.9 (299.6-443.7)	369.5 (300.8-445.7)	0.4	0.01 (0 to 0.02)
Namibia	5547 (4533-6708)	9228 (7544-11175)	66.4	369.4 (301.9-447.1)	367.9 (300.7-445.1)	-0.4	-0.01 (-0.01 to -0.01)
South Africa	142806 (117633-171815)	209688 (172748-251435)	46.8	369.9 (305.2-443.4)	373.6 (307.8-448.3)	1.0	0.04 (0.03 to 0.05)
Eswatini	3182 (2627-3840)	4428 (3652-5345)	39.1	361.6 (297.9-436.5)	367 (302.5-442.8)	1.5	0.05 (0.04 to 0.06)
Zimbabwe	41161 (33414-49518)	58254 (47289-70070)	41.5	368.9 (299.6-443.8)	364.4 (295.9-438.7)	-1.2	-0.05 (-0.05 to -0.04)
**Western sub-Saharan Africa**	769073 (634591-917695)	1807743 (1490123-2160089)	135.1	375.3 (309.6-449)	370.6 (305.5-443.3)	-1.3	-0.05 (-0.06 to -0.04)
Benin	19417 (15867-23477)	50508 (41289-61100)	160.1	367.3 (301.8-442.6)	370.3 (304.2-446.2)	0.8	0.02 (0.02 to 0.03)
Burkina Faso	37889 (31176-45491)	89977 (74066-107956)	137.5	364.5 (299.6-438.4)	366.7 (301.3-441.1)	0.6	0.02 (0.02 to 0.02)
Cameroon	41455 (34051-50085)	115568 (94893-139756)	178.8	370.6 (305-447.7)	373 (307-450.6)	0.6	0.09 (0.08 to 0.09)
Cabo Verde	1377 (1122-1666)	2179 (1779-2638)	58.2	366 (299.1-443.1)	380.4 (311-460.6)	3.9	0.02 (0.01 to 0.02)
Chad	23940 (19399-28790)	66431 (53795-79878)	177.5	367.1 (299.5-443.5)	373.5 (304.8-450.9)	1.7	0 (0 to 0.01)

Côte d'Ivoire	49757 (40807-59815)	105148 (86276-126365)	111.3	379 (309.3-455.4)	379.7 (309.9-456.1)	0.2	-0.04 (-0.05 to -0.04)
Gambia	3999 (3260-4806)	8870 (7224-10669)	121.8	376.3 (305.7-453.3)	371.9 (302.2-447.6)	-1.2	-0.05 (-0.06 to -0.04)
Ghana	59991 (49415-72498)	122115 (100698-147528)	103.6	372.5 (306.2-448.2)	368.7 (303.1-444)	-1.0	-0.04 (-0.05 to -0.04)
Guinea	24422 (19896-29283)	49909 (40602-59849)	104.4	369.2 (300.9-443.8)	368.1 (300-442.7)	-0.3	-0.02 (-0.02 to -0.01)
Guinea-Bissau	3992 (3240-4807)	7475 (6068-9001)	87.2	365.3 (295.6-441.1)	366.2 (296.3-442.2)	0.2	0 (0 to 0.01)
Liberia	7724 (6293-9299)	18928 (15416-22793)	145.1	370.8 (302.4-448)	375.6 (306.1-453.7)	1.3	0.05 (0.04 to 0.06)
Mali	34524 (28196-41494)	88081 (71928-105777)	155.1	370.3 (303.3-445.4)	373.3 (305.6-449)	0.8	0.03 (0.02 to 0.03)
Mauritania	8266 (6767-9997)	15800 (12936-19106)	91.2	374.6 (306.6-453)	374.4 (306.5-452.8)	-0.1	-0.01 (-0.01 to -0.01)
Niger	32367 (26675-38995)	94529 (77961-113835)	192.1	372.7 (306.5-449.2)	371.2 (305.3-447.4)	-0.4	-0.02 (-0.03 to -0.02)
Nigeria	360020 (297636-430110)	847527 (701421-1015824)	135.4	379.5 (313.3-454.5)	369.1 (304.5-441.9)	-2.7	-0.1 (-0.13 to -0.08)
Sao Tome and Principe	485 (393-582)	805 (651-968)	66.2	372.4 (300.9-447.5)	376.1 (304-452)	1.0	0.04 (0.03 to 0.05)
Senegal	30415 (24664-36640)	60185 (48847-72575)	97.9	371.6 (301.6-447.5)	375.6 (304.9-452.4)	1.1	0.04 (0.04 to 0.04)
Sierra Leone	14324 (11715-17177)	32663 (26697-39178)	128.0	369.6 (302.1-444.6)	372.4 (304.3-447.7)	0.8	0.05 (0.04 to 0.06)
Togo	14682 (11941-17707)	31018 (25237-37444)	111.3	367.6	368.3	0.2	0 (0 to 0.01)

				(299.7-443.6)	(300.3-444.6)		
**Eastern sub-Saharan Africa**	776444 (638587-926809)	1667223 (1372086-1991501)	114.7	378.2 (311.7-454.2)	378.4 (311.7-454.4)	0.1	0 (0 to 0)
Burundi	22445 (18456-27038)	48252 (39678-58109)	115.0	372 (305.8-448.1)	378.7 (311.7-456.5)	1.8	0.08 (0.07 to 0.09)
Comoros	1889 (1551-2277)	2803 (2306-3380)	48.4	379.1 (311.5-457.5)	381.4 (313.4-460.4)	0.6	0 (0 to 0.01)
Djibouti	2065 (1694-2479)	4897 (4020-5877)	137.1	393.8 (324.1-473.3)	393.1 (323.6-472.5)	-0.2	0 (0 to 0.01)
Eritrea	12296 (10052-14724)	27030 (22122-32421)	119.8	374.1 (305.9-449.6)	376.8 (307.9-452.7)	0.7	0.02 (0.02 to 0.03)
Ethiopia	211512 (173745-253595)	439965 (361753-527851)	108.0	381.5 (314.2-457.9)	383.2 (315.6-459.4)	0.4	0.01 (0 to 0.02)
Kenya	95473 (78434-114447)	202203 (166312-242819)	111.8	380.7 (313.4-457.8)	381.1 (313.8-457.9)	0.1	0.01 (0 to 0.01)
Madagascar	48507 (39611-58300)	107351 (87574-128998)	121.3	378.6 (308.5-456.2)	378.4 (308.4-455.9)	-0.1	0 (0 to 0)
Malawi	38569 (31362-46392)	73683 (59926-88672)	91.0	373.9 (305-449.8)	372.1 (303.6-447.9)	-0.5	-0.03 (-0.03 to -0.02)
Mozambique	51926 (42374-62763)	118485 (96737-143154)	128.2	369 (301-445.4)	369.6 (301.5-446.2)	0.2	0 (0 to 0.01)
Rwanda	28972 (23900-34810)	50089 (41292-60336)	72.9	372.5 (306.7-448.3)	372.4 (306.6-448.2)	0.0	0 (-0.02 to 0.02)
Somalia	29671 (24188-35948)	84110 (68570-101928)	183.5	381.7 (311.3-462.6)	379 (308.8-459.5)	-0.7	-0.02 (-0.03 to -0.02)
South Sudan	24403 (19993-29165)	37683 (30864-44999)	54.4	391.1 (321.5-469.5)	378.8 (311.3-454.6)	-3.1	-0.11 (-0.12 to -0.09)

United Republic of Tanzania	104284 (85332-125555)	226325 (185245-272497)	117.0	373.2 (305.4-450.5)	373.5 (305.5-450.8)	0.1	0 (0 to 0)
Uganda	71661 (58509-86022)	169727 (138581-203749)	136.8	381.3 (311.5-457.4)	378.8 (309.3-454.5)	-0.7	-0.02 (-0.02 to -0.01)
Zambia	32202 (26483-38748)	73287 (60265-88257)	127.6	375.5 (308.2-452)	375.8 (308.5-452.4)	0.1	0 (0 to 0)
**Central sub-Saharan Africa**	221912 (180990-266895)	522574 (426207-628311)	135.5	370.4 (303-446.3)	370.8 (303.3-446.9)	0.1	-0.01 (-0.01 to 0)
Angola	41586 (33794-50166)	119408 (96965-144153)	187.1	375.3 (305.9-452.8)	366.7 (298.9-442.6)	-2.3	-0.08 (-0.09 to -0.07)
Central African Republic	10875 (8886-13126)	20847 (17026-25167)	91.7	367.7 (299.2-443.9)	367 (298.6-442.9)	-0.2	0 (0 to 0)
Congo	9697 (7926-11664)	20503 (16770-24710)	111.4	368 (301.5-444.2)	371.9 (304.7-448.8)	1.1	0.03 (0.02 to 0.03)
Democratic Republic of the Congo	154139 (125512-185794)	349249 (284437-420369)	126.6	369.6 (301.1-446.4)	372.2 (303.2-449.6)	0.7	0.03 (0.02 to 0.03)
Equatorial Guinea	1699 (1403-2043)	5883 (4861-7067)	246.2	362.6 (299.2-437)	380.6 (314.7-458.7)	5.0	0.19 (0.18 to 0.21)
Gabon	3917 (3183-4741)	6682 (5420-8098)	70.6	373.1 (303.3-451.9)	369.1 (300.1-446.9)	-1.1	-0.04 (-0.04 to -0.04)

Note: ASD, autism spectrum disorders; ASPR, age-standardized prevalence rate; SDI, sociodemographic index.

eTable 2. Number and age-standardized rates of incidence for ASD globally and by SDI group, GBD region and super-region, country, and territory in 1990 and 2019.

	Incident cases No. (95% UI)			ASIR/100,000 No. (95% UI)			
	**1990**	**2019**	**Change, %**	**1990**	**2019**	**Change, %**	**EAPC No. (95% CI)**
**Global**	602887.2 (501382-718288.3)	603789.9 (501680.1-720096.5)	0.1	9.2 (7.6-10.9)	9.3 (7.7-11.1)	1.1	0.06 (0.04 to 0.07)
High SDI	75696 (63921-89058)	72189 (60836-84979)	-4.6	13.3 (11.3-15.7)	14.5 (12.3-17.1)	9.0	0.36 (0.31 to 0.41)
High-middle SDI	100983 (83515-120117)	77479 (64215-	1.7	10.2 (8.4-12.1)	10.3 (8.6-12.3)	1.0	0.03 (0 to 0.06)
Middle SDI	173883 (143431-208737)	92332)	0.1	8.4 (6.9-10.1)	8.4 (6.9-10.1)	0.0	-0.04 (-0.06 to -0.02)
Low-middle SDI	148418 (122088-177306)	139286 (114750-167032)	1.1	8.2 (6.7-9.8)	8.4 (6.9-10)	2.4	0.07 (0.06 to 0.08)
Low SDI	103577 (85506-123644)	137109 (1126789-163455)	0.1	9.2 (7.6-10.9)	9.2 (7.6-11)	0.0	0.04 (0.03 to 0.04)
**High-income North America**	29857.6 (25080.8-35105.6)	33093.1 (27799.6-38830.2)	10.8	13.6 (11.4-16)	16.4 (13.8-19.3)	20.6	0.8 (0.67 to 0.93)
Canada	2918.4 (2537.1-3357)	2745.4 (2368.6-3134)	-5.9	15 (13-17.2)	15.2 (13.1-17.3)	1.3	0.02 (0.02 to 0.03)
Greenland	7.8 (6.4-9.3)	4.9 (4-5.9)	-36.6	13.5 (11-16)	12.8 (10.5-15.3)	-5.2	-0.06 (-0.17 to 0.05)
United States of America	26930.7 (22448.4-31826.5)	30342.3 (25390.9-35736.7)	12.7	13.4 (11.2-15.9)	16.5 (13.8-19.5)	23.1	0.87 (0.74 to 1.01)
**Australasia**	1669.3 (1396.2-1988)	1957 (1636-2333.3)	17.2	11 (9.2-13.1)	11.1	0.9	0.07 (0.05 to

					(9.3-13.2)		0.08)
Australia	1347.1 (1123.8-1608.7)	1637 (1365.7-1954.9)	21.5	10.9 (9.1-13)	11.1 (9.2-13.2)	1.8	0.08 (0.06 to 0.1)
New Zealand	322.2 (267.6-382.9)	320 (265.6-380.3)	-0.7	11.2 (9.3-13.3)	11.2 (9.3-13.3)	0.0	0.02 (0 to 0.04)
**High-income Asia Pacific**	14603 (12197.8-17336.6)	10411.5 (8699.3-12344.7)	-28.7	15.4 (12.9-18.3)	15.7 (13.1-18.6)	1.9	0.11 (0.09 to 0.14)
Brunei Darussalam	48.2 (39.6-57.9)	45.4 (37.4-54.6)	-5.7	14.3 (11.8-17.2)	14.8 (12.2-17.8)	3.5	0.16 (0.13 to 0.18)
Japan	9409.5 (7879.4-11144.3)	7234.4 (6059.8-8548.2)	-23.1	15.9 (13.3-18.8)	16.7 (13.9-19.7)	5.0	0.18 (0.16 to 0.2)
Singapore	384.8 (318.1-460.7)	397.8 (328.9-476.3)	3.4	15.7 (13-18.8)	14.5 (12-17.3)	-7.6	-0.15 (-0.26 to -0.04)
Republic of Korea	4760.5 (3935.4-5664.2)	2733.9 (2260.3-3250.8)	-42.6	14.7 (12.1-17.5)	13.9 (11.5-16.5)	-5.4	-0.11 (-0.19 to -0.04)
**Western Europe**	31690.9 (26878.7-37290.6)	29330.7 (24870.9-34482.4)	-7.4	14.2 (12.1-16.7)	14.2 (12-16.7)	0.0	-0.02 (-0.03 to -0.01)
Andorra	4.2 (3.4-5)	4.2 (3.4-5)	0.5	16.5 (13.6-19.8)	18.6 (15.3-22.3)	12.7	0.51 (0.36 to 0.67)
Austria	623.2 (513.1-738)	588.1 (484.1-696.5)	-5.6	13.9 (11.5-16.5)	13.8 (11.4-16.4)	-0.7	-0.04 (-0.05 to -0.02)
Belgium	839.3 (698-1003.7)	811.4 (674.7-970.3)	-3.3	13.9 (11.6-16.7)	13.7 (11.4-16.4)	-1.4	-0.06 (-0.07 to -0.05)
Cyprus	97.9 (81.3-116.4)	101.8 (84.5-121)	3.9	14.8 (12.3-17.6)	13.9 (11.6-16.6)	-6.1	-0.19 (-0.23 to -0.14)
Denmark	319.4 (309.4-332.4)	313.5 (303.9-326)	-1.8	10.4 (10.1-10.9)	10.3 (10-10.7)	-1.0	-0.05 (-0.07 to -0.04)

Finland	432.3 (358.7-515.7)	327.3 (271.6-390.5)	-24.3	13.6 (11.3-16.3)	13.5 (11.2-16.2)	-0.7	-0.03 (-0.04 to -0.03)
France	4099.1 (3489.8-4924.6)	3922.1 (3343.5-4569.8)	-4.3	11.1 (9.5-13.4)	11.4 (9.7-13.3)	2.7	0.1 (0.08 to 0.12)
Germany	5905.7 (4883.6-7091.7)	4945.7 (4089.9-5938.9)	-16.3	13.8 (11.4-16.5)	13.4 (11.1-16.1)	-2.9	-0.2 (-0.24 to -0.15)
Greece	682.4 (568.2-819.4)	574.8 (478.7-690.3)	-15.8	13.6 (11.4-16.4)	13.9 (11.6-16.7)	2.2	0.12 (0.1 to 0.15)
Iceland	30.6 (25.4-36.2)	28 (23.3-33.1)	-8.4	13.7 (11.4-16.2)	13.7 (11.3-16.1)	0.0	0.04 (0.01 to 0.07)
Ireland	376.4 (310.9-448.9)	437 (360.9-521.2)	16.1	14.6 (12-17.4)	15 (12.4-17.9)	2.7	0.09 (0.06 to 0.11)
Israel	617.4 (515.7-739.6)	1160.9 (969.7-1390.8)	88.0	12.3 (10.3-14.8)	12.4 (10.4-14.9)	0.8	0.01 (0 to 0.02)
Italy	3804.3 (3170.9-4507.2)	2960.3 (2467.6-3520.9)	-22.2	14.2 (11.8-16.8)	13.9 (11.5-16.5)	-2.1	-0.11 (-0.13 to -0.1)
Luxembourg	32.6 (27-38.9)	43.2 (35.8-51.5)	32.5	13.6 (11.3-16.2)	13.6 (11.3-16.2)	0.0	-0.06 (-0.08 to -0.03)
Malta	37 (30.4-44)	28.6 (23.6-34.1)	-22.5	13.7 (11.3-16.3)	13.9 (11.4-16.5)	1.5	0.04 (0.02 to 0.07)
Netherlands	1456.1 (1193-1748.1)	1304.6 (1068.9-1566)	-10.4	15.3 (12.5-18.4)	15.2 (12.4-18.2)	-0.7	-0.02 (-0.03 to -0.01)
Norway	345.7 (288.8-412.9)	326.7 (272.1-387.9)	-5.5	11.9 (10-14.2)	11.8 (9.8-14)	-0.8	-0.06 (-0.08 to -0.04)
Portugal	772.5 (636.5-918.3)	528.3 (435.2-628)	-31.6	14 (11.5-16.6)	13.7 (11.3-16.3)	-2.1	-0.08 (-0.09 to -0.06)
Spain	2714.6 (2246.8-3238.6)	2529.6 (2093.8-3017.9)	-6.8	14.2	14.3	0.7	0.05 (0.03 to

				(11.8-17)	(11.9-17.1)		0.06)
Sweden	1020.2 (850.5-1203.9)	977.5 (820.4-1154.8)	-4.2	17 (14.2-20.1)	17.1 (14.4-20.2)	0.6	0.02 (-0.01 to 0.05)
Switzerland	571.6 (473.4-684.2)	592.6 (490.8-709.4)	3.7	14.3 (11.8-17.1)	13.9 (11.5-16.6)	-2.8	-0.14 (-0.16 to -0.12)
United Kingdom	6878.2 (5745.7-8114.6)	6794.9 (5675.9-8023.7)	-1.2	17.9 (15-21.2)	18 (15-21.2)	0.6	0.01 (0 to 0.02)
**Southern Latin America**	6093.9 (5094.4-7271)	5784.1 (4838.4-6900.2)	-5.1	12.1 (10.2-14.5)	12.5 (10.4-14.9)	3.3	0.1 (0.08 to 0.13)
Argentina	4045.9 (3359.4-4824.8)	4156.2 (3451.1-4956.3)	2.7	12.2 (10.1-14.5)	12.5 (10.4-14.9)	2.5	0.12 (0.1 to 0.15)
Chile	1719.7 (1436.6-2047.6)	1351 (1128.6-1608.6)	-21.4	12 (10.1-14.3)	12.4 (10.4-14.8)	3.3	-0.04 (-0.05 to -0.03)
Uruguay	328.1 (270.7-390.7)	276.6 (228.3-329.4)	-15.7	12.4 (10.2-14.8)	12.6 (10.4-15)	1.6	0.09 (0.07 to 0.11)
**Eastern Europe**	14258.9 (11833.5-17032.7)	11166.3 (9243.9-13324.2)	-21.7	10.2 (8.4-12.1)	10.3 (8.5-12.3)	1.0	0.05 (0.03 to 0.06)
Belarus	684.5 (565.2-823.9)	506.8 (418.5-610)	-26.0	10.1 (8.4-12.2)	10.2 (8.5-12.3)	1.0	0.05 (0.04 to 0.06)
Estonia	103.7 (85.1-123.8)	65.5 (53.7-78.2)	-36.8	10 (8.2-12)	10.2 (8.4-12.2)	2.0	0.03 (-0.03 to 0.09)
Latvia	179.5 (147.4-213.9)	95.1 (78.1-113.4)	-47.0	10.2 (8.4-12.2)	10.3 (8.5-12.3)	1.0	0.04 (-0.02 to 0.09)
Lithuania	278.9 (230.8-335.1)	135.6 (112.2-162.9)	-51.4	10.3 (8.6-12.4)	10.4 (8.6-12.5)	1.0	0.07 (0.03 to 0.11)
Republic of Moldova	379.9 (311.1-457.2)	158.9 (130.1-191.2)	-58.2	10.3 (8.4-12.3)	10.3 (8.4-12.4)	0.0	-0.01 (-0.02 to 0)

Russian Federation	9439.6 (7796.3-11235.6)	8269.3 (6817.2-9836.4)	-12.4	10.2 (8.4-12.1)	10.3 (8.5-12.3)	1.0	0.05 (0.04 to 0.06)
Ukraine	3192.9 (2621.6-3845.1)	1935.2 (1588.8-2329.8)	-39.4	10.1 (8.3-12.1)	10.3 (8.5-12.5)	2.0	0.05 (0.01 to 0.08)
**Central Europe**	7549.8 (6233.9-8947.8)	4859.6 (4014.1-5763.8)	-35.6	9.4 (7.8-11.1)	9.4 (7.8-11.2)	0.0	0 (-0.02 to 0.01)
Albania	404.3 (329.6-486.1)	171 (139.4-205.6)	-57.7	9.8 (8-11.8)	10.2 (8.3-12.2)	4.1	0.18 (0.08 to 0.28)
Bosnia and Herzegovina	297.9 (242.7-355.4)	118.7 (96.7-141.6)	-60.1	9.1 (7.4-10.9)	9.2 (7.5-11)	1.1	-0.07 (-0.17 to 0.02)
Bulgaria	448.9 (368.5-536)	271.6 (223-324.3)	-39.5	9.5 (7.8-11.3)	9.4 (7.7-11.2)	-1.1	-0.02 (-0.06 to 0.02)
Croatia	244.6 (199.5-293)	160 (130.5-191.7)	-34.6	9.1 (7.4-10.9)	9.3 (7.6-11.2)	2.2	0.12 (0.09 to 0.15)
Czechia	582.1 (472.2-704.8)	493.9 (400.7-598.1)	-15.1	9.5 (7.7-11.5)	9.4 (7.6-11.4)	-1.1	-0.02 (-0.03 to 0)
Hungary	560.1 (459.1-673.1)	373.1 (305.8-448.3)	-33.4	9.4 (7.7-11.3)	9.4 (7.7-11.3)	0.0	0.01 (0 to 0.03)
North Macedonia	161 (131.5-193.4)	101.3 (82.7-121.6)	-37.1	9.6 (7.8-11.5)	9.5 (7.7-11.4)	-1.0	-0.02 (-0.11 to 0.07)
Montenegro	43.9 (35.9-53.1)	30.2 (24.7-36.5)	-31.3	9.1 (7.5-11)	9.4 (7.7-11.3)	3.3	0.08 (0.06 to 0.1)
Poland	2426.4 (1997-2878.3)	1659 (1365.5-1970.4)	-31.6	9.4 (7.7-11.2)	9.5 (7.8-11.3)	1.1	-0.01 (-0.04 to 0.02)
Romania	1302 (1069.6-1555.7)	781.9 (642.2-934.3)	-39.9	9.1 (7.5-10.9)	9.4 (7.7-11.2)	3.3	0.04 (0.01 to 0.06)
Serbia	623.1 (509.5-747.8)	362.3 (296.3-434.9)	-41.8	9.7 (8-11.7)	9.1 (7.5-11)	-6.2	-0.26 (-0.34 to -0.18)

Slovakia	355.6 (291.7-427.3)	251.2 (206.1-301.9)	-29.4	9.4 (7.7-11.3)	9.4 (7.7-11.3)	0.0	0.01 (-0.01 to 0.03)
Slovenia	99.8 (80.9-119.9)	85.5 (69.2-102.6)	-14.4	9.4 (7.6-11.2)	9.4 (7.6-11.3)	0.0	0.02 (0 to 0.03)
**Central Asia**	9227 (7599.3-11021)	8923.4 (7349.2-10654.3)	-3.3	9.9 (8.1-11.8)	9.9 (8.1-11.8)	0.0	0.03 (0 to 0.06)
Armenia	374.9 (304.6-453.9)	182.7 (148.5-221.2)	-51.3	10.3 (8.4-12.5)	10.1 (8.2-12.3)	-1.9	-0.09 (-0.14 to -0.04)
Azerbaijan	960 (788.5-1149.2)	734.6 (604-878.9)	-23.5	10.5 (8.7-12.6)	10.7 (8.8-12.8)	1.9	0.13 (0.04 to 0.21)
Georgia	382.5 (314.8-4566.1)	215.8 (177.6-257.4)	-43.6	9.5 (7.9-11.4)	9.8 (8.1-11.8)	3.2	0.3 (-0.53 to 1.13)
Kazakhstan	1658.9 (1356.8-1987.5)	1645.4 (1345.8-1971.2)	-0.8	9.7 (8-11.7)	9.7 (7.9-11.6)	0.0	-0.04 (-0.06 to -0.03)
Kyrgyzstan	577.8 (475.7-689.6)	675.3 (555.9-805.7)	16.9	9.6 (7.9-11.4)	9.7 (8-11.5)	1.0	0.08 (0.06 to 0.11)
Mongolia	348.7 (282.8-417.9)	388.8 (315.3-466)	11.5	10 (8.1-11.9)	10.1 (8.2-12.1)	1.0	0.02 (-0.04 to 0.08)
Tajikistan	973.8 (798.7-1166.5)	1189.4 (975.6-1424.8)	22.1	9.9 (8.1-11.9)	9.8 (8-11.7)	-1.0	-0.06 (-0.08 to -0.05)
Turkmenistan	628.2 (512.9-753.8)	531.3 (433.8-637.5)	-15.4	10 (8.2-12)	9.9 (8.1-11.9)	-1.0	-0.06 (-0.08 to -0.04)
Uzbekistan	3322.2 (2715.5-4009)	3360 (2746.7-4056)	1.1	9.7 (7.9-11.7)	9.8 (8-11.8)	1.0	0.01 (0.01 to 0.02)
**Central Latin America**	21860.2 (18042.8-26050.3)	19339.8 (15980.3-23070.3)	-11.5	9.2 (7.6-11)	9.2 (7.6-10.9)	0.0	-0.01 (-0.02 to 0)
Colombia	3961.5 (3226.1-4765.8)	3391.7 (2762.2-4080.2)	-14.4	8.9 (7.2-10.7)	8.9 (7.3-10.7)	0.0	0.02 (-0.05 to 0.08)
Costa Rica	338.4 (275.5-407.8)	278 (226.3-335.1)	-17.8	8.7 (7.1-10.5)	8.7 (7.1-10.5)	0.0	-0.04 (-0.05 to

							-0.04)
El Salvador	697.7 (566.4-837)	473.9 (384.7-568.6)	-32.1	8.9 (7.2-10.7)	8.8 (7.1-10.5)	-1.1	-0.03 (-0.04 to -0.03)
Guatemala	1438.2 (1182.3-1719.1)	1721.7 (1415.3-2057.9)	19.7	8.9 (7.3-10.6)	8.7 (7.2-10.4)	-2.2	-0.06 (-0.09 to -0.03)
Honduras	762.7 (616.2-916)	984.5 (795.4-1182.3)	29.1	8.9 (7.2-10.6)	8.7 (7.1-10.5)	-2.2	-0.06 (-0.07 to -0.05)
Mexico	11606.5 (9552.8-13773.6)	9738 (8036.1-11527.5)	-16.1	9.7 (8-11.6)	9.7 (8-11.5)	0.0	-0.03 (-0.03 to -0.02)
Nicaragua	605.2 (495.2-724)	548.8 (449-656.5)	-9.3	8.8 (7.2-10.5)	8.7 (7.1-10.4)	-1.1	-0.02 (-0.03 to -0.01)
Panama	247.5 (200.7-296.8)	319.5 (259.1-383.1)	29.1	8.7 (7.1-10.5)	8.7 (7-10.4)	0.0	-0.03 (-0.04 to -0.01)
Venezuela (Bolivarian Republic of)	2202.3 (1802-2661.6)	1883.6 (1541.2-2276.4)	-14.5	8 (6.6-9.7)	8.3 (6.8-10)	3.8	0.14 (0.06 to 0.23)
**Andean Latin America**	5235.2 (4322-6298.6)	5658.7 (4672-6810.1)	8.1	9.1 (7.5-10.9)	9 (7.4-10.8)	-1.1	-0.05 (-0.08 to -0.02)
Bolivia (Plurinational State of)	1046.8 (857.1-1266.9)	1393.8 (1141.2-1686.9)	33.2	9 (7.4-10.9)	9 (7.4-10.9)	0.0	0.03 (0.01 to 0.06)
Ecuador	1289.2 (1051.7-1553.3)	1486.2 (1212.4-1790.7)	15.3	9 (7.3-10.8)	8.9 (7.2-10.7)	-1.1	-0.07 (-0.08 to -0.06)
Peru	2899.2 (2374-3496.1)	2778.7 (2275.4-3350.6)	-4.2	9.1 (7.5-11)	9.1 (7.4-10.9)	0.0	-0.07 (-0.12 to -0.02)
**Caribbean**	3895.6 (3214.8-4673.5)	3499.1 (2882.4-4185.3)	-10.2	9 (7.4-10.8)	9 (7.4-10.7)	0.0	0.05 (0.03 to 0.07)
Antigua and Barbuda	5.7 (4.7-6.9)	4.2 (3.4-5.1)	-26.4	8.8 (7.1-10.5)	8.8 (7.2-10.6)	0.0	0.04 (0.03 to 0.05)

Bahamas	22.4 (18.3-27.1)	17.3 (14.1-20.9)	-22.8	8.6 (7.1-10.5)	8.7 (7.1-10.5)	1.2	-0.04 (-0.08 to 0.01)
Barbados	17.8 (14.7-21.4)	12.1 (10-14.5)	-32.1	8.9 (7.3-10.6)	8.8 (7.2-10.6)	-1.1	-0.03 (-0.04 to -0.01)
Belize	25 (20.4-29.9)	32.2 (26.2-38.6)	28.9	8.8 (7.2-10.6)	8.8 (7.1-10.5)	0.0	-0.04 (-0.06 to -0.02)
Bermuda	3.8 (3.1-4.6)	2.2 (1.8-2.6)	-42.8	8.9 (7.3-10.7)	8.9 (7.2-10.7)	0.0	0.02 (0.01 to 0.03)
Cuba	773.9 (627.9-932.7)	447.3 (362.9-539.1)	-42.2	8.9 (7.2-10.7)	8.9 (7.2-10.7)	0.0	-0.01 (-0.04 to 0.01)
Dominica	7.3 (6-8.8)	3.5 (2.9-4.2)	-52.0	8.9 (7.3-10.8)	8.8 (7.2-10.6)	-1.1	-0.08 (-0.11 to -0.05)
Dominican Republic	1047.4 (849.8-1253.3)	980.8 (795.8-1173.6)	-6.4	9.1 (7.4-10.9)	9 (7.3-10.7)	-1.1	-0.08 (-0.08 to -0.07)
Grenada	8.3 (6.8-10)	6 (4.8-7.2)	-28.4	8.7 (7.1-10.5)	8.9 (7.3-10.7)	2.3	0.07 (0.06 to 0.09)
Guyana	97.2 (79.1-116.6)	61.2 (49.8-73.5)	-37.0	9.1 (7.4-11)	8.9 (7.3-10.7)	-2.2	-0.05 (-0.09 to -0.01)
Haiti	1061.8 (868.2-1277.4)	1426.7 (1166.6-1716.4)	34.4	9 (7.3-10.8)	9 (7.4-10.8)	0.0	0 (-0.02 to 0.02)
Jamaica	235.8 (193.4-283.1)	152.5 (125.1-183.1)	-35.3	8.8 (7.2-10.5)	8.8 (7.2-10.6)	0.0	0.05 (0.04 to 0.06)
Puerto Rico	278.4 (227.4-335.1)	108.1 (88.3-130.1)	-61.2	8.8 (7.2-10.6)	8.9 (7.3-10.7)	1.1	0.04 (0.01 to 0.07)
Saint Lucia	16.2 (13.2-19.4)	7.5 (6.2-9)	-53.4	8.9 (7.3-10.7)	8.8 (7.2-10.6)	-1.1	-0.03 (-0.04 to -0.02)
Saint Vincent and the	11.3 (9.2-13.7)	6.4 (5.2-7.8)	-43.1	8.9 (7.2-10.7)	8.9 (7.2-10.7)	0.0	-0.02 (-0.03 to

Grenadines							-0.01)
Suriname	40.9 (33.9-49.3)	38.9 (32.2-46.9)	-4.9	9.4 (7.8-11.3)	9 (7.5-10.9)	-4.3	-0.15 (-0.17 to -0.13)
Trinidad and Tobago	99.1 (80.7-119.9)	65.4 (53.2-79.1)	-34.0	8.8 (7.2-10.7)	8.8 (7.2-10.7)	0.0	0.01 (0 to 0.01)
United States Virgin Islands	9.7 (7.9-11.6)	5.4 (4.4-6.5)	-44.1	8.9 (7.3-10.8)	8.9 (7.3-10.8)	0.0	0 (-0.03 to 0.02)
**Tropical Latin America**	15756.3 (13009.1-18759.5)	14381.9 (11893.9-17113.6)	-8.7	9.2 (7.6-11)	9.3 (7.7-11.1)	1.1	0.06 (0.03 to 0.08)
Brazil	15199.2 (12525-18107.9)	13830.8 (11415-16469.2)	-9.0	9.2 (7.6-11)	9.3 (7.7-11.1)	1.1	0.06 (0.03 to 0.08)
Paraguay	557.1 (456.4-673.8)	551 (451.4-666.4)	-1.1	9.1 (7.4-11)	9.1 (7.5-11)	0.0	0.01 (0.01 to 0.02)
**East Asia**	111604.1 (91661.2-133842.6)	71074.2 (58298.2-84933.9)	-36.3	9.3 (7.6-11.1)	9.6 (7.9-11.5)	3.2	0.03 (-0.04 to 0.09)
China	108205.9 (88914.7-129774.2)	69469 (56972.9-83020.4)	-35.8	9.4 (7.7-11.3)	9.7 (8-11.6)	3.2	0.05 (0 to 0.11)
Democratic People's Republic of Korea	2522.8 (2042.5-3048.4)	1142.9 (925.3-1381)	-54.7	7.3 (5.9-8.8)	6.9 (5.6-8.4)	-5.5	-0.25 (-0.3 to -0.2)
Taiwan (province of China)	875.3 (741-1031.4)	462.2 (391.2-544.6)	-47.2	5.5 (4.7-6.5)	5.4 (4.6-6.4)	-1.8	-0.03 (-0.08 to 0.02)
**Southeast Asia**	49221 (40292.4-58766.9)	42760.9 (34987-51275.6)	-13.1	8.2 (6.7-9.7)	8.2 (6.7-9.8)	0.0	0.02 (0.01 to 0.02)
Cambodia	1735.1 (1403.5-2100.6)	1444.4 (1168.4-1748.7)	-16.8	8.3 (6.7-10)	8.2 (6.7-10)	-1.2	0.08 (0.04 to 0.12)
Indonesia	18431.1 (15038.7-21962.1)	14827.7 (12099.1-17703)	-19.6	8.1 (6.6-9.6)	8 (6.6-9.6)	-1.2	0 (-0.01 to 0)
Lao People's Democratic	648.6 (531.6-783)	680.5 (557.8-821.5)	4.9	8.1 (6.7-9.8)	8.3 (6.8-10)	2.5	0.09 (0.06 to

Republic							0.12)
Malaysia	1927.3 (1555.9-2314.2)	2106 (1700.3-2528.9)	9.3	8.1 (6.5-9.7)	8.1 (6.5-9.7)	0.0	0.02 (0 to 0.03)
Maldives	34.4 (28-41.5)	33.2 (27-40.1)	-3.4	8.2 (6.6-9.9)	8.2 (6.7-9.9)	0.0	0.05 (0.02 to 0.07)
Mauritius	85.9 (70.5-102.8)	49.6 (40.7-59.3)	-42.3	8 (6.5-9.5)	8 (6.6-9.6)	0.0	0.03 (0.01 to 0.05)
Myanmar	5274.3 (4260.1-6344.3)	4140.8 (3344.6-4981.2)	-21.5	8.3 (6.7-10)	8.3 (6.7-10)	0.0	0 (-0.01 to 0.01)
Philippines	8258.4 (6756.5-9849.7)	10544.3 (8629.8-12593.7)	27.7	8.3 (6.8-9.9)	8.3 (6.8-9.9)	0.0	0 (-0.01 to 0.02)
Sri Lanka	1427.4 (1164.3-1717.7)	1179.3 (962-1419.3)	-17.4	8.3 (6.8-10)	8.3 (6.7-10)	0.0	0 (-0.01 to 0.01)
Seychelles	6.6 (5.4-7.9)	5.8 (4.7-6.9)	-12.7	8.2 (6.7-9.8)	8.1 (6.7-9.7)	-1.2	-0.04 (-0.04 to -0.04)
Thailand	4163.5 (3426.7-4987.8)	2289.6 (1884.4-2742.9)	-45.0	8.2 (6.7-9.8)	8.2 (6.7-9.8)	0.0	0.02 (-0.03 to 0.06)
Timor-Leste	149.3 (121.9-179.2)	152 (124.1-182.5)	1.8	8.7 (7.1-10.5)	8.3 (6.7-9.9)	-4.6	-0.23 (-0.25 to -0.21)
Viet Nam	7013.7 (5706.1-8442.3)	5251.6 (4274.4-6321.5)	-25.1	8 (6.5-9.6)	8.2 (6.7-9.9)	2.5	0.09 (0.06 to 0.12)
**Oceania**	807.9 (660.9-977.1)	1505.5 (1228.3-1822.1)	86.3	7.6 (6.2-9.2)	7.6 (6.2-9.3)	0.0	0.03 (0.01 to 0.04)
American Samoa	6.2 (5.1-7.6)	3.8 (3.1-4.6)	-39.2	7.6 (6.2-9.3)	7.5 (6.1-9.2)	-1.3	-0.06 (-0.06 to -0.06)
Micronesia (Federated States of)	12.7 (10.3-15.3)	7 (5.7-8.5)	-44.7	7.6 (6.2-9.2)	7.5 (6.1-9.1)	-1.3	-0.05 (-0.05 to -0.05)
Fiji	68 (55.7-81.9)	63 (51.6-75.9)	-7.3	7.5 (6.1-9)	7.5 (6.1-9)	0.0	0 (-0.02 to

							0.02)
Guam	13.8 (11.2-16.7)	11.8 (9.5-14.2)	-14.9	7.7 (6.2-9.2)	7.5 (6.1-9.1)	-2.6	-0.08 (-0.1 to -0.07)
Kiribati	9.4 (7.7-11.3)	11 (9-13.2)	17.0	7.6 (6.2-9.1)	7.6 (6.2-9.1)	0.0	0 (-0.01 to 0.01)
Marshall Islands	5.5 (4.5-6.6)	4.5 (3.7-5.4)	-17.7	7.5 (6.1-9.1)	7.6 (6.2-9.2)	1.3	0.05 (0.02 to 0.07)
Northern Mariana Islands	4.6 (3.7-5.6)	1.8 (1.5-2.2)	-60.6	7.6 (6.1-9.1)	7.8 (6.3-9.4)	2.6	0.1 (0.01 to 0.18)
Papua New Guinea	542.4 (441.8-656.5)	1202.4 (979.5-1455.4)	121.7	7.6 (6.2-9.2)	7.7 (6.2-9.3)	1.3	0.04 (0.02 to 0.06)
Samoa	11.3 (9.2-13.6)	12.8 (10.4-15.4)	13.1	6.9 (5.6-8.3)	6.9 (5.6-8.4)	0.0	0.07 (0.05 to 0.08)
Solomon Islands	53.2 (43.3-64.5)	76.8 (62.6-93.2)	44.4	7.8 (6.3-9.4)	7.7 (6.3-9.3)	-1.3	-0.06 (-0.08 to -0.04)
Tonga	9.1 (7.4-11)	8.2 (6.7-9.9)	-10.1	7.3 (5.9-8.8)	7.3 (5.9-8.8)	0.0	0 (-0.01 to 0.02)
Vanuatu	20.8 (16.8-25.3)	27.5 (22.2-33.4)	32.2	7.6 (6.2-9.2)	7.5 (6-9)	-1.3	-0.1 (-0.12 to -0.09)
**North Africa and Middle East**	44220.8 (36224.9-53111.2)	45002.1 (36856.6-53911.9)	1.8	7.9 (6.5-9.5)	7.7 (6.3-9.3)	-2.5	-0.06 (-0.07 to -0.05)
Afghanistan	1914.7 (1545.2-2312)	5431.2 (4383.1-6558.7)	183.7	7.7 (6.2-9.3)	7.6 (6.2-9.2)	-1.3	0.17 (0.04 to 0.3)
Algeria	2758.3 (2242.5-3313.8)	3175.9 (2582-3815.7)	15.1	7.5 (6.1-9)	7.6 (6.2-9.1)	1.3	-0.04 (-0.09 to 0.01)
Bahrain	49.1 (39.8-59.3)	46.2 (37.5-55.8)	-5.8	7.5 (6.1-9)	7.4 (6-9)	-1.3	-0.03 (-0.1 to 0.03)

Egypt	7028.3 (5671.1-8533.9)	7579.9 (6116.1-9203.7)	7.8	7.8 (6.3-9.4)	7.5 (6-9.1)	-3.8	-0.15 (-0.17 to -0.14)
Iran (Islamic Republic of)	7625.9 (6312.5-9118.7)	6041.2 (4992.1-7192.6)	-20.8	8.9 (7.4-10.7)	9.3 (7.7-11.1)	4.5	0.04 (-0.01 to 0.08)
Iraq	2615.1 (2122.1-3150.5)	3409.9 (2767-4108.2)	30.4	7.8 (6.3-9.4)	7.4 (6-8.9)	-5.1	-0.01 (-0.08 to 0.05)
Jordan	493.5 (401.7-595.4)	860.1 (700.1-1037.7)	74.3	7.5 (6.1-9.1)	7.3 (5.9-8.8)	-2.7	-0.21 (-0.29 to -0.14)
Kuwait	141.6 (115.5-171.3)	217.1 (177.1-262.6)	53.3	7.7 (6.3-9.4)	7.4 (6-8.9)	-3.9	0 (-0.07 to 0.07)
Lebanon	421.3 (342.9-510.5)	397.4 (323.5-481.6)	-5.7	8 (6.5-9.7)	7.9 (6.4-9.5)	-1.3	-0.12 (-0.22 to -0.03)
Libya	546.9 (452.7-661.4)	285.7 (236.5-345.5)	-47.8	7.6 (6.3-9.2)	7.3 (6-8.8)	-3.9	-0.15 (-0.21 to -0.09)
Morocco	2890.8 (2352.7-3471.8)	2180.9 (1774.9-2619.1)	-24.6	7.6 (6.2-9.2)	7.6 (6.2-9.1)	0.0	-0.05 (-0.07 to -0.03)
Oman	226.8 (184.9-272.4)	265.8 (216.7-319.1)	17.2	7.1 (5.8-8.6)	7.1 (5.8-8.5)	0.0	-0.07 (-0.13 to -0.01)
Palestine	343.4 (277.9-413.9)	448.1 (362.7-540.2)	30.5	7.5 (6.1-9.1)	7.5 (6.1-9)	0.0	-0.03 (-0.04 to -0.01)
Qatar	37.7 (30.6-45.4)	95.2 (77.4-114.6)	152.5	7.5 (6.1-9)	7.3 (5.9-8.7)	-2.7	-0.12 (-0.15 to -0.09)
Saudi Arabia	1927.6 (1563.4-2326.5)	1629.5 (1321.7-1966.8)	-15.5	7.5 (6.1-9.1)	7.4 (6-9)	-1.3	-0.04 (-0.05 to -0.03)
Sudan	3797.8 (3088.8-4590.8)	4330.6 (3522.1-5234.8)	14.0	8.1 (6.6-9.8)	7.7 (6.3-9.3)	-4.9	-0.22 (-0.24 to -0.21)
Syrian Arab Republic	1852.4 (1514.1-2227.5)	831.3 (679.5-999.6)	-55.1	7.7 (6.3-9.2)	7.4 (6.1-8.9)	-3.9	0.2 (0 to 0.39)

Tunisia	865.3 (703.2-1043.7)	601.2 (488.6-725.2)	-30.5	7.6 (6.2-9.2)	7.5 (6.1-9)	-1.3	-0.09 (-0.11 to -0.07)
Turkey	6033.9 (4930.1-7317)	3514.1 (2871.2-4261.3)	-41.8	7.6 (6.2-9.3)	7.5 (6.1-9.1)	-1.3	-0.11 (-0.15 to -0.07)
United Arab Emirates	166.6 (134.8-201.3)	201.3 (162.9-243.4)	20.9	7.5 (6.1-9.1)	7.5 (6-9)	0.0	0.03 (-0.04 to 0.11)
Yemen	2454.2 (2015.6-2956.2)	3413.5 (2803.5-4111.6)	39.1	7.6 (6.2-9.1)	7.6 (6.3-9.2)	0.0	-0.03 (-0.05 to 0)
**South Asia**	129805.7 (106720-155432)	121885.7 (100112.7-145614.1)	-6.1	7.6 (6.2-9)	7.6 (6.2-9.1)	0.0	0.01 (0 to 0.01)
Bangladesh	13928.2 (11349.8-16705.3)	9788.3 (7976.7-11740.1)	-29.7	7.4 (6-8.9)	7.6 (6.2-9.1)	2.7	0.08 (0.07 to 0.09)
Bhutan	84.6 (68.9-102.6)	50.9 (41.5-61.7)	-39.8	8 (6.5-9.7)	7.9 (6.5-9.6)	-1.3	-0.07 (-0.13 to -0.02)
India	94539.1 (77557.5-113868.7)	84788.7 (69569.9-102182.7)	-10.3	7.5 (6.2-9)	7.5 (6.2-9.1)	0.0	0 (-0.01 to 0)
Nepal	3063.9 (2484.6-3692.7)	2282.8 (1851.2-2751.3)	-25.5	7.9 (6.4-9.5)	7.7 (6.3-9.3)	-2.5	-0.07 (-0.07 to -0.06)
Pakistan	18189.9 (14835.4-21772.9)	24975 (20410.5-29777.4)	37.3	7.9 (6.4-9.4)	7.8 (6.4-9.3)	-1.3	-0.04 (-0.05 to -0.03)
**Southern sub-Saharan Africa**	7211 (5956.4-8602.9)	7814.8 (6467.7-9333.7)	8.4	9.8 (8.1-11.7)	9.8 (8.1-11.7)	0.0	0 (-0.01 to 0.02)
Botswana	188.1 (153.2-226.2)	224.4 (182.7-269.8)	19.3	9.6 (7.8-11.6)	9.7 (7.9-11.7)	1.0	0.02 (0.01 to 0.03)
Lesotho	293 (238.5-354.3)	219.3 (178.5-265.1)	-25.2	10.1 (8.2-12.2)	9.9 (8.1-12)	-2.0	-0.12 (-0.14 to -0.1)
Namibia	223.9 (181.6-271.2)	291.6 (236.4-353.1)	30.2	9.6 (7.8-11.7)	9.7 (7.9-11.8)	1.0	0.04 (0.02 to

							0.05)
South Africa	4537.8 (3755.2-5400.1)	4851.6 (4011.2-5790.8)	6.9	9.7 (8-11.6)	9.8 (8.1-11.7)	1.0	0.03 (0.01 to 0.05)
Eswatini	150.4 (123.9-180)	140.1 (115.4-167.6)	-6.9	9.9 (8.1-11.8)	10 (8.2-11.9)	1.0	0.04 (0.01 to 0.07)
Zimbabwe	1817.8 (1477.1-2177.3)	2088 (1696.7-2500.9)	14.9	9.9 (8.1-11.9)	9.8 (8-11.8)	-1.0	-0.06 (-0.07 to -0.05)
**Western sub-Saharan Africa**	42800.3 (35511.1-51287.4)	77149.4 (64001.2-92453.7)	80.3	10 (8.3-12)	9.8 (8.2-11.8)	-2.0	-0.07 (-0.08 to -0.06)
Benin	1109.6 (906.5-1338.6)	2365.8 (1932.8-2854.1)	113.2	9.7 (7.9-11.7)	9.8 (8-11.8)	1.0	0.03 (0.03 to 0.03)
Burkina Faso	2142.3 (1760.2-2574.4)	4392.7 (3609.3-5278.9)	105.1	9.7 (8-11.7)	9.8 (8-11.7)	1.0	0.01 (0 to 0.01)
Cameroon	2176.5 (1785.8-2618.8)	4230.2 (3470.7-5090)	94.4	9.8 (8-11.8)	9.8 (8-11.8)	0.0	-0.04 (-0.06 to -0.03)
Cabo Verde	54.1 (44-65.1)	50.2 (40.8-60.4)	-7.2	9.6 (7.8-11.5)	9.8 (7.9-11.7)	2.1	-0.01 (-0.02 to 0.01)
Chad	1503.6 (1223.9-1796)	3695 (3007.3-4413.4)	145.7	9.9 (8-11.8)	9.8 (8-11.7)	-1.0	-0.03 (-0.04 to -0.03)
Côte d'Ivoire	2692.9 (2196.5-3228.8)	4178.2 (3407.9-5009.6)	55.2	9.9 (8-11.8)	9.9 (8.1-11.9)	0.0	0.03 (0.02 to 0.05)
Gambia	233.7 (192.3-280.7)	335.7 (276.2-403.2)	43.6	10.2 (8.4-12.2)	9.9 (8.1-11.8)	-2.9	-0.16 (-0.18 to -0.13)
Ghana	2845.8 (2320.9-3418.3)	4002.7 (3264.6-4808.1)	40.7	9.9 (8-11.8)	9.8 (8-11.8)	-1.0	0 (-0.01 to 0.01)
Guinea	1446.8 (1174.1-1726.3)	2235.5 (1814.2-2667.4)	54.5	9.6 (7.8-11.4)	9.8 (7.9-11.6)	2.1	-0.02 (-0.06 to 0.01)
Guinea-Bissau	218.4 (178.1-261.8)	293.7 (239.6-352.1)	34.5	9.8 (8-11.7)	9.8 (8-11.8)	0.0	0.01 (0 to 0.02)

Liberia	416.1 (342.8-500.5)	635.3 (523.4-764.1)	52.7	10.8 (8.9-13)	9.9 (8.1-11.9)	-8.3	-0.01 (-0.14 to 0.11)
Mali	2125.3 (1745.4-2546.1)	4509.3 (3702.7-5401.9)	112.2	10 (8.2-12)	9.8 (8-11.7)	-2.0	-0.08 (-0.1 to -0.05)
Mauritania	423.1 (345.9-509)	508.9 (416-612.2)	20.3	9.9 (8.1-11.9)	9.7 (7.9-11.7)	-2.0	-0.09 (-0.1 to -0.07)
Niger	2083 (1721.3-2489.3)	5347.8 (4419.1-6390.9)	156.7	9.8 (8.1-11.7)	9.7 (8-11.6)	-1.0	-0.04 (-0.05 to -0.03)
Nigeria	20160.4 (16794-24209.1)	35741.7 (29659-42898.6)	77.3	10.2 (8.5-12.2)	9.9 (8.2-11.9)	-2.9	-0.13 (-0.14 to -0.11)
Sao Tome and Principe	21.9 (17.8-26.3)	21.7 (17.7-26.1)	-0.6	9.8 (8-11.8)	9.7 (7.9-11.6)	-1.0	-0.09 (-0.1 to -0.07)
Senegal	1609.6 (1302.7-1925.6)	2180.5 (1764.7-2608.5)	35.5	9.8 (7.9-11.7)	9.8 (7.9-11.7)	0.0	0 (0 to 0.01)
Sierra Leone	772.1 (634.5-926.6)	1311.9 (1078-1574.2)	69.9	10.3 (8.4-12.3)	9.8 (8.1-11.8)	-4.9	-0.09 (-0.17 to -0.02)
Togo	763.8 (623.6-919.1)	1111.6 (907.6-1337.7)	45.5	9.8 (8-11.8)	9.8 (8-11.8)	0.0	0.03 (0.02 to 0.03)
**Eastern sub-Saharan Africa**	42923.2 (35394-51306.9)	67379.8 (55601.5-80553.8)	57.0	10.1 (8.3-12.1)	10 (8.2-11.9)	-1.0	-0.03 (-0.04 to -0.02)
Burundi	1257 (1034.5-1506.7)	2208.6 (1817.4-2647.3)	75.7	9.9 (8.2-11.9)	9.9 (8.1-11.9)	0.0	-0.05 (-0.12 to 0.02)
Comoros	96.1 (78.8-115.6)	79.4 (65.1-95.6)	-17.3	10.2 (8.3-12.2)	10 (8.2-12.1)	-2.0	0.03 (0.01 to 0.06)
Djibouti	105.2 (86.2-125.5)	168.2 (137.7-200.6)	59.9	9.9 (8.1-11.9)	10.2 (8.3-12.1)	3.0	-0.06 (-0.13 to 0)
Eritrea	646.2 (525.9-770.9)	945.4 (769.3-1127.8)	46.3	10.9 (8.9-13)	9.9 (8.1-11.8)	-9.2	-0.04 (-0.12 to 0.05)

Ethiopia	11798.5 (9669-14132.8)	17558.5 (14374-21119.8)	48.8	10 (8.2-11.9)	10 (8.2-12.1)	0.0	-0.01 (-0.03 to 0)
Kenya	4825.5 (3985.6-5740.4)	6399.9 (5288.3-7597.2)	32.6	10.1 (8.3-12)	9.9 (8.2-11.8)	-2.0	-0.06 (-0.07 to -0.05)
Madagascar	2563.5 (2099.6-3073.2)	4088.7 (3348.9-4901.6)	59.5	10.1 (8.2-12.1)	10 (8.2-12)	-1.0	-0.02 (-0.03 to -0.02)
Malawi	2225.3 (1815-2662.8)	2578 (2102.6-3084.7)	15.8	10 (8.1-11.9)	9.8 (8-11.7)	-2.0	-0.19 (-0.25 to -0.13)
Mozambique	2862.3 (2341.8-3411.9)	5334.2 (4364.2-6358.4)	86.4	10.2 (8.4-12.2)	9.9 (8.1-11.8)	-2.9	-0.02 (-0.08 to 0.04)
Rwanda	1509.1 (1250.3-1809.6)	1667.8 (1381.7-1999.8)	10.5	9.9 (8.2-11.9)	9.9 (8.2-11.8)	0.0	-0.09 (-0.33 to 0.15)
Somalia	1598.3 (1303.4-1930.6)	4147.3 (3382-5009.6)	159.5	10.9 (8.9-13.1)	10 (8.1-12)	-8.3	-0.03 (-0.11 to 0.04)
South Sudan	1196 (979.2-1424.6)	1726.1 (1413.3-2056.1)	44.3	10 (8.2-11.9)	10 (8.2-11.9)	0.0	0.15 (0.06 to 0.24)
United Republic of Tanzania	5842.2 (4779.6-7015.8)	9853.1 (8061.3-11832.5)	68.7	10.1 (8.2-12.1)	10 (8.2-12)	-1.0	-0.03 (-0.03 to -0.02)
Uganda	4513.8 (3681.6-5399.1)	7661.8 (6249.3-9164.6)	69.7	10.2 (8.3-12.2)	10.2 (8.3-12.1)	0.0	-0.02 (-0.03 to -0.01)
Zambia	1852.7 (1529.7-2215.5)	2908.8 (2401.7-3478.4)	57.0	10.1 (8.3-12.1)	9.9 (8.2-11.8)	-2.0	-0.07 (-0.08 to -0.06)
**Central sub-Saharan Africa**	12595.6 (10322.3-15048)	20812.4 (17058.3-24864.1)	65.2	9.9 (8.1-11.8)	9.8 (8-11.7)	-1.0	-0.02 (-0.02 to -0.01)
Angola	2405 (1956.3-2897)	5168.2 (4203.9-6225.2)	114.9	9.9 (8-11.9)	9.8 (7.9-11.8)	-1.0	-0.06 (-0.07 to -0.04)
Central African Republic	596.4 (490.6-716.7)	922.9 (759.3-1109.2)	54.8	9.9 (8.1-11.9)	9.8 (8.1-11.8)	-1.0	-0.01 (-0.02 to

							-0.01)
Congo	473.7 (388.2-567.6)	675.2 (553.4-809)	42.5	9.9 (8.1-11.9)	9.8 (8-11.7)	-1.0	-0.06 (-0.07 to -0.05)
Democratic Republic of the Congo	8825.2 (7211.4-10508.6)	13666.7 (11166.5-16274.9)	54.9	9.9 (8.1-11.7)	9.8 (8-11.6)	-1.0	-0.03 (-0.03 to -0.02)
Equatorial Guinea	107.7 (88.6-129.3)	179.5 (147.6-215.5)	66.7	9.8 (8.1-11.8)	9.7 (8-11.6)	-1.0	-0.04 (-0.05 to -0.03)
Gabon	187.6 (152.3-226.5)	199.9 (162.3-241.3)	6.6	10 (8.1-12)	9.8 (8-11.8)	-2.0	-0.06 (-0.07 to -0.06)

Note: ASD, autism spectrum disorders; ASIR, age-standardized incidence rate; GBD, Global Burden of Disease; SDI, sociodemographic index.

eTable 3. Number and age-standardized rates of DALYs for ASD globally and by SDI group, GBD region and super-region, country, and territory in 1990 and 2019.

	DALYs No. (95% UI)			Age-standardized DALY rate/100,000 No. (95% UI)			
	**1990**	**2019**	**Change, %**	**1990**	**2019**	**Change, %**	**EAPC No. (95% CI)**
**Global**	3105909.1 (2025303-4514467.3)	4306615.4 (2821511.9-6232360.5)	38.7	56.7 (37-82.2)	56.3 (36.8-81.5)	-0.7	-0.02 (-0.03 to -0.01)
High SDI	654788.5 (429488.9-932431.3)	822966.8 (541875.3-1175034)	25.7	82.2 (53.8-117.4)	88.2 (57.9-126.3)	7.3	0.3 (0.24 to 0.35)
High-middle SDI	713058.6 (465214-1032596.5)	838198.3 (546345-1211677.1)	17.5	61.8 (40.3-89.5)	62.1 (40.4-89.8)	0.5	0.02 (0.02 to 0.02)
Middle SDI	875926.9 (569680.5-1284765.4)	1155444.4 (754950.5-1679972.4)	31.9	48.8 (31.8-71.5)	49.2 (32.1-71.6)	0.8	0.02 (0.01 to 0.03)
Low-middle SDI	566660.6 (372962.5-827870.7)	856771 (561754.3-1245320.1)	51.2	47.2 (31-68.5)	47.6 (31.2-69.1)	0.8	0.03 (0.02 to 0.03)
Low SDI	293882.7 (191832.2-427362.2)	630884.7 (414559-917939.8)	114.7	51.3 (33.6-74.5)	51.9 (34.1-75.2)	1.2	0.04 (0.04 to 0.04)

**High-income North America**	216780.9 (142091.7-307645.7)	326234.1 (215046.6-462932.4)	50.5	79.9 (52.3-113.3)	96.9 (63.7-137.7)	21.3	0.81 (0.68 to 0.95)
Canada	24223.8 (15957.6-33905.8)	31128.1 (21013.8-44021.5)	28.5	91.6 (60.3-129)	92.9 (62.4-131.9)	1.4	0.05 (0.03 to 0.06)
Greenland	43.7 (28.2-63.1)	41 (26.7-58.5)	-6.1	76.7 (49.6-110.4)	75.9 (49.4-108.6)	-1.0	-0.03 (-0.03 to -0.02)
United States of America	192508.5 (125434.9-274538.7)	295059.8 (194672-419941.6)	53.3	78.6 (51.3-111.8)	97.3 (64-138.8)	23.8	0.9 (0.75 to 1.05)
**Australasia**	13165.8 (8637.1-18709.4)	18037.7 (11831.4-26143.5)	37.0	66.4 (43.6-94.3)	66.3 (43.5-96.1)	-0.2	0 (0 to 0)
Australia	10927.8 (7191-15451.2)	15241.7 (9971.4-22130.1)	39.5	66.3 (43.5-93.9)	66.3 (43.3-96.3)	0.0	0 (0 to 0.01)
New Zealand	2237.9 (1465.5-3253.2)	2796 (1831.5-4001.8)	24.9	66.7 (43.6-97)	66.4 (43.2-95.3)	-0.4	-0.01 (-0.01 to -0.01)
**High-income Asia Pacific**	159572.1 (104215.6-229854.6)	162304.3 (106518.2-233834.5)	1.7	94.5 (61.6-136.4)	97.3 (63.6-141)	3.0	0.11 (0.09 to 0.13)
Brunei Darussala	240 (156.2-343.3)	382.5 (251.9-548.4)	59.4	87.4 (57.1-12	87.1 (57.1-12	-0.3	0.01 (-0.

m				4.3)	5.4)		02 to 0.04)
Japan	119160.2 (77831.7-171430.3)	116063.8 (76362.6-168477.3)	-2.6	99.3 (64.8-142.8)	103.8 (67.7-149.7)	4.5	0.17 (0.14 to 0.2)
Singapore	2747.5 (1785.1-4024.6)	4840.7 (3172.6-7059)	76.2	89.6 (58.5-130.5)	90.3 (58.8-131.7)	0.8	0.05 (0.02 to 0.07)
Republic of Korea	37424.4 (24234.6-54071.9)	41017.3 (26331.9-59285.8)	9.6	82.5 (53.6-119.6)	84.1 (53.8-121.9)	1.9	0.06 (0.05 to 0.06)
**Western Europe**	319675.5 (208840-454860.1)	353844.8 (233430.4-504526.4)	10.7	87.5 (57.1-125)	88.7 (58.1-127.1)	1.4	0.05 (0.05 to 0.05)
Andorra	48 (31.5-69.1)	68.4 (44.6-98.7)	42.4	90.2 (59.2-129.5)	88.6 (57.7-127.5)	-1.8	-0.07 (-0.09 to -0.06)
Austria	6265.1 (4072.5-9095.1)	7046.9 (4603.9-10178)	12.5	85.5 (55.3-124.7)	86.6 (56.4-125.5)	1.3	0.04 (0.03 to 0.04)
Belgium	8096.6 (5323.8-11707.6)	9034.3 (5949.1-12951.3)	11.6	85.8 (56.2-123.8)	85.9 (56.1-123.5)	0.1	0 (-0.01 to 0)
Cyprus	666.1 (436.2-957.8)	1063.8 (696.3-1540.1)	59.7	86.4 (56.7-124.2)	85.9 (56.1-123.9)	-0.6	-0.03 (-0.

							04 to -0.03)
Denmark	3182 (2191.8-4404)	3529.9 (2467.2-4815.9)	10.9	65.2 (44.7-90.5)	65.5 (45.5-89.8)	0.5	0.01 (0.01 to 0.01)
Finland	4002 (2618.8-5718.5)	4276.7 (2819.2-6098.9)	6.9	84.1 (55.2-120.3)	85.1 (56.2-121.6)	1.2	0.04 (0.04 to 0.05)
France	38000.9 (25004.2-55112.2)	42603.5 (28560.8-60447.2)	12.1	68.7 (45.1-100.3)	70 (46.7-99.6)	1.9	0.08 (0.07 to 0.1)
Germany	64381.2 (41931.7-91828.7)	66860.3 (43604.6-96715)	3.9	86 (56-124.3)	87.3 (56.7-127.1)	1.5	0.04 (0.03 to 0.04)
Greece	8521.2 (5514-12320.8)	8002.3 (5219.6-11574.1)	-6.1	85.9 (55.8-124.3)	85.9 (56-125)	0.0	-0.01 (-0.02 to 0)
Iceland	216.1 (141.4-313)	283.7 (186.3-406.8)	31.3	86 (56.3-124.3)	86.5 (56.6-124.1)	0.6	0.02 (0.01 to 0.03)
Ireland	3318.6 (2157.2-4829.6)	4330.1 (2817.2-6281.9)	30.5	92.9 (60.5-135)	92.5 (60.4-134.5)	-0.4	-0.01 (-0.02 to 0)
Israel	3885.3 (2565.3-5575.8)	7170.7 (4729.3-10303.4)	84.6	77.6 (51.1-111.5)	78.1 (51.5-112.1)	0.6	0.03 (0.03 to

							0.03)
Italy	45815.4 (30043.2-65768.5)	46564.6 (30491.8-67279.9)	1.6	85.7 (56.3-122.5)	86.6 (56.7-124.4)	1.1	0.04 (0.04 to 0.04)
Luxembourg	312 (201.2-451.4)	505.3 (326.5-727.7)	62.0	86.2 (55.4-125.2)	87.2 (56.1-125.6)	1.2	0.04 (0.04 to 0.05)
Malta	315.9 (204.7-456.6)	354.8 (231.3-509.8)	12.3	87.1 (56.9-126)	88.3 (57-127.4)	1.4	0.05 (0.05 to 0.05)
Netherlands	13825.3 (8942.9-19832.1)	15302.3 (9937.9-21705)	10.7	96.2 (62.2-138.1)	96.3 (62.2-137.9)	0.1	0 (0 to 0.01)
Norway	2909.2 (1898.2-4200.3)	3691.2 (2416.5-5319.3)	26.9	72.3 (47-104.2)	73.9 (48.4-107.2)	2.2	0.06 (0.05 to 0.07)
Portugal	8099.1 (5292.6-11762.1)	7932.1 (5122.1-11540.5)	-2.1	83.2 (54.4-121)	83.6 (54-123.2)	0.5	0.02 (0.01 to 0.02)
Spain	32782.3 (21491.6-46882.6)	37095.6 (24305.4-53409.1)	13.2	88 (57.6-125.7)	88.6 (58.1-128.1)	0.7	0.04 (0.03 to 0.05)
Sweden	8502 (5566.2-12160.4)	10198.1 (6717.5-14571.5)	19.9	105.5 (69-151.8)	108 (71.2-155.3)	2.4	0.11 (0.1 to 0.11)
Switzerland	5634.9 (3711.3-8110.9)	7072.1 (4610.9-10148.8)	25.5	86.7 (57.1-12	87.6 (57.1-12	1.0	0.03 (0.0

				5.6)	6.3)		2 to 0.04)
United Kingdom	60588.6 (40078.6-86441.4)	70495.6 (46384.6-100354.6)	16.4	111 (72.9-158.5)	112.3 (73.7-160.5)	1.2	0.05 (0.05 to 0.05)
**Southern Latin America**	36786.2 (23775-52798.3)	47677.8 (30967-68854.5)	29.6	73.5 (47.5-105.4)	73.7 (47.9-106.2)	0.3	0.01 (0.01 to 0.01)
Argentina	24445.3 (15771.9-35421.9)	32298.5 (20836.3-46477.1)	32.1	73.3 (47.3-106.1)	73.4 (47.3-105.6)	0.1	0.01 (0.01 to 0.01)
Chile	10074.1 (6517.6-14823.6)	12987.4 (8526.3-18919.6)	28.9	74 (48-109.3)	74.6 (48.9-108.6)	0.8	0.02 (0.02 to 0.02)
Uruguay	2265.4 (1472.8-3275.7)	2389.6 (1548-3423.3)	5.5	73.7 (47.8-106.6)	73.7 (47.7-106)	0.0	0 (0 to 0)
**Eastern Europe**	131358.8 (85364.5-190702.9)	116804.4 (76388.7-169066.5)	-11.1	60.2 (39.2-87.3)	60.8 (39.6-87.8)	1.0	0.03 (0.03 to 0.03)
Belarus	6123.7 (3946.4-8820.9)	5322.3 (3458.1-7705.2)	-13.1	60.5 (39.1-87.1)	61.3 (39.4-88.8)	1.3	0.03 (0.03 to 0.03)
Estonia	914.5 (592.2-1329.3)	731.6 (479.9-1073.3)	-20.0	60.8 (39.4-88.8)	61.9 (40.7-91.2)	1.8	0.07 (0.06 to 0.08)
Latvia	1531.5 (1006.2-2224.8)	1046.2 (678.8-1535.5)	-31.7	60.5 (39.8-87.	61.4 (39.7-89.	1.5	0.05 (0.0

				9)	8)		5 to 0.06)
Lithuania	2195.3 (1421-3185)	1554.4 (1013.4-2259.7)	-29.2	61.8 (40.1-89.9)	62.5 (40.5-90.9)	1.1	0.04 (0.03 to 0.04)
Republic of Moldova	2660 (1713.6-3895)	2067.5 (1338.6-3043.5)	-22.3	60 (38.6-87.8)	60.8 (39.6-89.9)	1.3	0.05 (0.05 to 0.05)
Russian Federation	87778.1 (57090.9-127096)	81887.6 (53362-118119.5)	-6.7	60.1 (39-86.9)	60.7 (39.4-87.9)	1.0	0.03 (0.03 to 0.04)
Ukraine	30155.7 (19620.2-43747.1)	24194.7 (15806.2-34845.6)	-19.8	60.2 (39-87.4)	60.8 (39.7-87.7)	1.0	0.03 (0.03 to 0.03)
**Central Europe**	67584.8 (43770.1-97177)	59275.5 (38737.4-85387.3)	-12.3	56.5 (36.6-81.2)	57.1 (37.2-82)	1.1	0.04 (0.04 to 0.05)
Albania	1953 (1259.5-2850.8)	1451.5 (941.1-2115.3)	-25.7	56.9 (36.6-82.7)	56.8 (36.7-82.3)	-0.2	0.01 (-0.01 to 0.03)
Bosnia and Herzegovina	2621.9 (1700.2-3815.1)	1743.1 (1144.7-2501)	-33.5	57.8 (37.5-83.9)	57.7 (37.4-82.9)	-0.2	0.03 (0.01 to 0.04)
Bulgaria	4630.7 (3039.9-6742.7)	3496.5 (2287-5040.9)	-24.5	56.2 (36.8-81.6)	56.6 (37-81.8)	0.7	0.03 (0.02 to 0.03

							)
Croatia	2690.8 (1732.8-3938.2)	2212.3 (1454.3-3232.3)	-17.8	57.4 (36.8-84.3)	57.9 (37.9-84.8)	0.9	0.03 (0.03 to 0.04)
Czechia	5665.1 (3656.5-8147.8)	5595.1 (3640.2-8081.9)	-1.2	57.3 (36.8-82.4)	57.8 (37.6-83.6)	0.9	0.04 (0.03 to 0.04)
Hungary	5583.9 (3627.1-7995.3)	4950.2 (3192.3-7131.3)	-11.3	56.7 (36.6-81.6)	57.1 (36.8-82.7)	0.7	0.04 (0.04 to 0.04)
North Macedonia	1155.8 (757.2-1666.1)	1174.2 (764.9-1668)	1.6	57.3 (37.5-82.6)	58 (37.7-83.2)	1.2	0.04 (0.04 to 0.05)
Montenegro	357.5 (230-520.9)	334.9 (215-490.5)	-6.3	57.4 (36.9-83.6)	57.8 (37.2-84.7)	0.7	0.02 (0.02 to 0.03)
Poland	21098.3 (13833.3-30406.2)	20003.2 (13096.8-28831.8)	-5.2	56.5 (37-81.3)	57.2 (37.3-82.2)	1.2	0.04 (0.04 to 0.05)
Romania	12659.6 (8229.5-18331.9)	9738.6 (6430.1-14091.6)	-23.1	55.6 (36.2-80.8)	56 (36.9-81.1)	0.7	0.04 (0.03 to 0.04)
Serbia	5128.5 (3350.9-7398.1)	4618 (3031.5-6711.2)	-10.0	56.2 (36.8-81.4)	57.4 (37.6-83.4)	2.1	0.08 (0.08 to 0.09)
Slovakia	2945.1 (1907.4-4283.4)	2863.8 (1853.9-4129.5)	-2.8	56.7 (36.6-82.	57.2 (37.1-82.	0.9	0.04 (0.0

				2)	7)		4 to 0.04)
Slovenia	1094.6 (706.1-1623.8)	1094.1 (723-1607.7)	0.0	57.6 (37.1-85. 2)	58.5 (38-84.6)	1.6	0.06 (0.0 6 to 0.07)
**Central Asia**	41257 (26947.9-60087. 5)	54402.4 (35733.3-78753.8)	31.9	56.9 (37.3-82. 7)	57.4 (37.7-83. 2)	0.9	0.04 (0.0 3 to 0.04)
Armenia	2018.6 (1317.3-2910.4)	1682.2 (1098.7-2431.6)	-16.7	57.9 (37.8-83. 5)	58.7 (38.2-85)	1.4	0.05 (0.0 5 to 0.06)
Azerbaijan	4355 (2866.1-6267.9)	5886.2 (3815.5-8464.7)	35.2	57.1 (37.6-82. 4)	58.2 (37.7-83. 3)	1.9	0.09 (0.0 8 to 0.09)
Georgia	3106.7 (2023.1-4544.1)	1979 (1284.4-2875.7)	-36.3	57.4 (37.3-83. 8)	58.2 (37.5-84. 6)	1.4	0.22 (-0. 6 to 1.05)
Kazakhsta n	9509.2 (6199.8-13962)	10402.4 (6764.5-15198.7)	9.4	56.5 (36.9-82. 6)	56.7 (36.9-83. 1)	0.4	0.01 (0.0 1 to 0.02)
Kyrgyzsta n	2660.1 (1755.4-3832.7)	3855.5 (2528-5603.9)	44.9	56.7 (37.3-82)	57.3 (37.7-83. 4)	1.1	0.03 (0.0 3 to 0.03)
Mongolia	1316.5 (860.5-1906.9)	1958.1 (1267.7-2856.2)	48.7	56.9 (36.9-82. 4)	56.6 (36.9-82. 2)	-0.5	-0.0 1 (-0. 01 to

							0)
Tajikistan	3302.2 (2147-4796.1)	5741.4 (3805.1-8274.7)	73.9	57.2 (37.4-83.5)	57.8 (38.3-83.4)	1.0	0.04 (0.04 to 0.05)
Turkmenistan	2256.8 (1474.4-3282.3)	3009.6 (1971.2-4379.9)	33.4	56.8 (37.1-82.9)	58.1 (38-84.5)	2.3	0.09 (0.08 to 0.09)
Uzbekistan	12731.9 (8300.7-18654.5)	19888 (12905.4-29188.1)	56.2	56.8 (36.9-83.1)	57.3 (37.2-84)	0.9	0.03 (0.03 to 0.04)
**Central Latin America**	93734.6 (61359.4-134649.9)	133792.5 (87312.6-194854.3)	42.7	53.7 (35.1-77.3)	53.6 (35-78.2)	-0.2	-0.01 (-0.01 to 0)
Colombia	17598.8 (11588.4-25583.5)	24093.4 (15696.9-34922.1)	36.9	51.3 (33.8-74.4)	51.5 (33.5-75.1)	0.4	0.02 (0.01 to 0.02)
Costa Rica	1687.5 (1096.5-2442.3)	2419.4 (1560.6-3529.7)	43.4	52.9 (34.4-76.2)	52.4 (34-76.3)	-0.9	-0.04 (-0.04 to -0.03)
El Salvador	2833.9 (1852.1-4113.3)	3147.8 (2035.2-4574)	11.1	50.4 (33-73.6)	50 (32.3-72.3)	-0.8	-0.02 (-0.03 to -0.02)
Guatemala	4358.6 (2797.1-6413.4)	9351.7 (6080-13730.4)	114.6	50.4 (32.7-74)	50.2 (32.5-73.	-0.4	-0.02

					4)		(-0.02 to -0.01)
Honduras	2612 (1694.9-3814.5)	5193.7 (3362.8-7633.4)	98.8	50.9 (33.1-74.1)	50.6 (32.7-74.4)	-0.6	-0.02 (-0.03 to -0.02)
Mexico	51559 (33878.5-74218.9)	70670.3 (46195.2-102006.5)	37.1	56.6 (37.1-81.6)	56.8 (37.1-82)	0.4	0 (0 to 0.01)
Nicaragua	2161.7 (1414.1-3166.8)	3457.9 (2257.2-5099.8)	60.0	50.9 (33.6-74.7)	51.5 (33.6-75.8)	1.2	0.04 (0.04 to 0.04)
Panama	1303.3 (854.3-1900.6)	2173 (1403.2-3161)	66.7	52.4 (34.3-76.2)	52.3 (33.8-76.1)	-0.2	-0.01 (-0.01 to 0)
Venezuela (Bolivarian Republic of)	9619.7 (6251.2-13997.6)	13285.3 (8665.1-19332.7)	38.1	48.2 (31.3-70.1)	48 (31.4-70.1)	-0.4	0.01 (0 to 0.02)
**Andean Latin America**	20988.7 (13725.6-30730.5)	33571.2 (22050.5-48663.1)	59.9	51.9 (33.9-75.6)	52.3 (34.4-75.9)	0.8	0.03 (0.03 to 0.04)
Bolivia (Plurinational State of)	3534.5 (2327.2-5189.9)	6437.6 (4226.4-9440.1)	82.1	51.3 (33.8-75.4)	52 (34.2-76.1)	1.4	0.05 (0.04 to 0.05)
Ecuador	5514.2	9274.9	68.2	51.9	52.1	0.4	0.01

	(3596.7-7997.2)	(6087-13366.1)		(33.7-75.7)	(34.2-75.4)		(0.01 to 0.02)
Peru	11940 (7826.1-17486.6)	17858.8 (11747.8-26236.1)	49.6	52 (34.2-76.4)	52.6 (34.6-77.3)	1.2	0.04 (0.04 to 0.05)
**Caribbean**	19089.4 (12528.4-27489.8)	24307.5 (15890.4-35203.9)	27.3	52.5 (34.5-75.8)	52.4 (34.2-75.9)	-0.2	0 (-0.01 to 0)
Antigua and Barbuda	32.4 (20.8-47.1)	45.1 (29.9-65.8)	39.2	52.3 (33.6-75.9)	52.8 (35-77.1)	1.0	0.05 (0.03 to 0.07)
Bahamas	138.5 (90.4-202.5)	190.5 (125.1-278.1)	37.6	51.8 (33.8-75.9)	51.9 (34.2-75.7)	0.2	-0.01 (-0.01 to 0)
Barbados	132.4 (84.9-193.7)	146 (95.3-211.5)	10.2	52.7 (33.9-77.4)	52.9 (34.7-76.4)	0.4	0.01 (0 to 0.01)
Belize	104.4 (68.7-152.6)	218.6 (143.4-323.1)	109.3	52.5 (34.7-76.7)	52 (34.2-76.7)	-1.0	-0.03 (-0.04 to -0.03)
Bermuda	31.1 (20.3-45.3)	31 (20.3-45.3)	-0.3	53.5 (34.8-77.6)	53.6 (34.7-77.8)	0.2	0.01 (0 to 0.02)
Cuba	5833.8	5670.2	-2.8	53.9	54	0.2	0.01

	(3750.9-8568)	(3711.9-8312.4)		(34.7-79.2)	(35.2-79.2)		(0.01 to 0.01)
Dominica	39.8 (26.3-58.3)	35 (23.2-51.1)	-12.1	52.5 (34.6-76.7)	53 (35-77.4)	1.0	0.03 (0.02 to 0.03)
Dominican Republic	3939.1 (2547.8-5734.4)	5747.7 (3726.8-8340.1)	45.9	51.6 (33.7-74.7)	52.3 (33.9-75.8)	1.4	0.07 (0.06 to 0.07)
Grenada	46.8 (30.8-67.8)	54.1 (35-78.4)	15.6	52.5 (34.6-76.2)	53.4 (34.5-77.4)	1.7	0.07 (0.06 to 0.08)
Guyana	418 (273.2-610.2)	400.7 (260.3-589.3)	-4.1	51.1 (33.7-74.6)	51.4 (33.3-75.5)	0.6	0.01 (0 to 0.03)
Haiti	3445.5 (2254.9-4957.1)	6563.1 (4292.3-9545)	90.5	50.6 (33-72.6)	50.5 (33.2-73.3)	-0.2	0 (-0.01 to 0.01)
Jamaica	1297.8 (857.5-1897.2)	1470.9 (948.1-2119.1)	13.3	52.9 (35.1-77.1)	53.2 (34.1-76.8)	0.6	0.02 (0.02 to 0.03)
Puerto Rico	1912.4 (1257.1-2775.7)	1691.3 (1110.2-2471.9)	-11.6	52.9 (34.8-77)	53.1 (34.7-77.6)	0.4	0.02 (0.01 to 0.02)
Saint Lucia	75.2 (49-109.9)	88.1 (57.5-129.9)	17.1	51.8 (33.9-75.5)	52.7 (34.3-78.1)	1.7	0.06 (0.05 to

							0.06)
Saint Vincent and the Grenadines	60.7 (39.8-89.8)	58.1 (38.2-84.2)	-4.3	52.3 (34.1-77.1)	52.9 (34.7-76.8)	1.1	0.04 (0.03 to 0.04)
Suriname	210.3 (137.3-304.8)	294.8 (193.1-433.3)	40.2	52.2 (34.2-76.2)	51.9 (34.1-76.4)	-0.6	-0.01 (-0.02 to -0.01)
Trinidad and Tobago	656.5 (427.9-954.1)	698.6 (454.7-1005.8)	6.4	52.7 (34.3-76.5)	52.9 (34.4-76.3)	0.4	0.02 (0.02 to 0.02)
United States Virgin Islands	56.6 (37.2-82.1)	50.5 (33.4-73.1)	-10.8	52.7 (34.7-76.3)	52.5 (34.8-76.1)	-0.4	0 (-0.01 to 0.01)
**Tropical Latin America**	86131.8 (56209-124848.2)	117552 (76818.8-170312.3)	36.5	53.8 (35.2-78.2)	53.9 (35.2-78)	0.2	0.01 (0.01 to 0.01)
Brazil	83806.7 (54763.7-121393.8)	113727 (74291.2-164759)	35.7	53.8 (35.2-78.1)	53.9 (35.2-78)	0.2	0.01 (0.01 to 0.01)
Paraguay	2325.1 (1495.8-3408.2)	3825 (2479.7-5613.1)	64.5	54.1 (35.1-79)	54.4 (35.5-79.5)	0.6	0.02 (0.02 to 0.02)
**East Asia**	680072.4 (444396-996213.7)	778339.3 (509696.8-1129871)	14.4	54.3 (35.4-79.6)	56.6 (37-82.7)	4.2	0.14 (0.13 to

							0.15)
China	664748.2 (433995.7-974658.5)	761041.6 (498324.6-1104699.5)	14.5	54.9 (35.8-80.6)	57.3 (37.4-83.8)	4.4	0.14 (0.13 to 0.15)
Democratic People's Republic of Korea	8384.4 (5440.5-12260.2)	10120.7 (6496.9-14820.6)	20.7	38.7 (25-56.2)	40.3 (25.9-58.9)	4.1	0.16 (0.15 to 0.17)
Taiwan (province of China)	6939.9 (4535.6-9910.2)	7177 (4776.7-10250.6)	3.4	33.6 (22-48.1)	33.3 (22.1-47.9)	-0.9	-0.03 (-0.04 to -0.03)
**Southeast Asia**	233298.1 (151876.5-343401.2)	321010.4 (208179.1-470065.7)	37.6	47.3 (30.9-69.5)	47.8 (31-70.1)	1.1	0.04 (0.04 to 0.05)
Cambodia	5185.4 (3381.1-7563.8)	8021.6 (5134.3-11612.6)	54.7	45.5 (30-66.1)	46.9 (30.1-67.9)	3.1	0.11 (0.11 to 0.12)
Indonesia	91886.1 (59995.7-135108.7)	123166.7 (80103.6-179318.3)	34.0	47 (30.7-69)	47.5 (30.9-69.1)	1.1	0.04 (0.04 to 0.05)
Lao People's Democratic Republic	2087.4 (1343.5-3037)	3526.1 (2297.7-5111.9)	68.9	46.5 (30.3-67.3)	47.5 (31-68.7)	2.2	0.08 (0.07 to 0.08)
Malaysia	9065 (5917.3-13081.9)	15573.9 (10150.5-22892.3)	71.8	48.8 (31.6-69.8)	49.6 (32.4-72.9)	1.6	0.07 (0.06 to 0.07

							)
Maldives	116.8 (76.1-170.5)	270.6 (175.6-397.7)	131.6	49.4 (32.3-72.3)	52.7 (34.4-77.1)	6.7	0.24 (0.18 to 0.3)
Mauritius	550.7 (358.4-802.2)	587.6 (383.6-849.6)	6.7	48.6 (31.8-70.8)	48.7 (31.8-70.6)	0.2	0 (0 to 0.01)
Myanmar	20414.2 (13463.7-30215.7)	25672.7 (16843.4-38050.3)	25.8	46.8 (30.9-69.3)	46.6 (30.6-69.1)	-0.4	-0.01 (-0.02 to -0.01)
Philippines	32315.5 (21049.4-47081.3)	55789.3 (36451.7-81071.4)	72.6	47.8 (31.2-69.3)	48.2 (31.6-70.1)	0.8	0.04 (0.03 to 0.04)
Sri Lanka	8857.3 (5741.4-12856.3)	10403.8 (6834.5-15214.8)	17.5	49.7 (32.2-72.1)	49 (32.2-71.7)	-1.4	-0.04 (-0.05 to -0.04)
Seychelles	36.6 (23.7-53.3)	49.6 (32.4-71.9)	35.5	48.6 (31.5-70.7)	49.8 (32.3-72.4)	2.5	0.11 (0.09 to 0.12)
Thailand	27986.4 (18197.4-40688.8)	31043.2 (20123.1-45087.6)	10.9	47.6 (31.1-69.5)	47.8 (31-69.5)	0.4	0.03 (0.02 to 0.04)
Timor-Leste	405.2 (263.6-592.6)	671.9 (438.9-968.7)	65.8	48 (31.2-70.3)	47.7 (31.2-69)	-0.6	0.01 (-0.01 to 0.03

							)
Viet Nam	34081.4 (22040.9-49905.2)	45812.9 (29973.9-67121.4)	34.4	47.2 (30.6-69)	48.4 (31.6-71.3)	2.5	0.09 (0.08 to 0.1)
**Oceania**	3049.5 (1985.2-4417.8)	6135.6 (3979.6-8860.3)	101.2	44 (28.7-63.9)	43.9 (28.6-63.2)	-0.2	0 (-0.01 to 0)
American Samoa	23.2 (15-34.4)	25.1 (16.3-36.5)	8.0	45.1 (29.2-66.6)	44.5 (28.9-65)	-1.3	-0.05 (-0.05 to -0.04)
Micronesia (Federated States of)	49.8 (32.4-72.9)	46.3 (29.9-67.8)	-7.0	44.1 (28.7-64.3)	44 (28.4-64)	-0.2	0 (-0.01 to 0.01)
Fiji	354.1 (230.1-516.4)	406.8 (264-596.4)	14.9	44.1 (28.7-63.9)	44.1 (28.6-64.4)	0.0	0 (0 to 0.01)
Guam	65.4 (42.1-95.8)	76.5 (49.8-112.6)	16.9	46 (29.4-67.7)	45.4 (29.6-66.9)	-1.3	-0.04 (-0.07 to -0.02)
Kiribati	33.9 (22.3-50.1)	53.3 (34.6-77.6)	57.0	42.7 (28.1-62.8)	42.6 (27.6-61.8)	-0.2	0 (-0.01 to 0.01)
Marshall Islands	22.1 (14.1-32.2)	25.9 (17.2-37.4)	17.3	44.1 (28.6-64.2)	44 (29-63.5)	-0.2	0 (0 to 0)

Northern Mariana Islands	21.7 (14.1-31.8)	18.6 (12.2-27.6)	-14.0	46.4 (30.3-67.8)	45.9 (30.3-67.7)	-1.1	-0.02 (-0.07 to 0.02)
Papua New Guinea	1922.6 (1246.2-2810.4)	4578.1 (2978.1-6636.5)	138.1	43.8 (28.6-63.9)	43.8 (28.6-63.3)	0.0	0.01 (0.01 to 0.02)
Samoa	79.2 (51.5-117.1)	98.4 (64.1-146.1)	24.2	45.3 (29.6-67)	44.9 (29.1-66.5)	-0.9	-0.03 (-0.03 to -0.02)
Solomon Islands	163.9 (105.9-234.7)	307.1 (200.1-443.6)	87.3	44.5 (29.1-63.6)	44 (28.6-63.2)	-1.1	-0.05 (-0.05 to -0.04)
Tonga	45.5 (29.7-65.5)	46.6 (30-68)	2.4	44 (28.7-63.2)	44 (28.4-63.7)	0.0	-0.01 (-0.03 to 0)
Vanuatu	72.1 (46.7-107.6)	135.9 (87.9-199.6)	88.5	44.3 (28.7-65.3)	43.9 (28.5-64.6)	-0.9	-0.03 (-0.03 to -0.03)
**North Africa and Middle**	170028 (110895.4-247697.3)	287616.5 (188299.3-418661.6)	69.2	46.3 (30.2-67.6)	46.4 (30.4-67.5)	0.2	0.01 (0.01 to 0.01

**East**							)
Afghanistan	5198.8 (3394.5-7531.1)	18286.2 (11991-26467.5)	251.7	42.1 (27.5-60.8)	43.4 (28.6-62.8)	3.1	0.08 (0.07 to 0.09)
Algeria	11998 (7789.5-17737.3)	18750.3 (12174.5-27499.3)	56.3	44.4 (28.9-65)	44.5 (28.9-65.6)	0.2	0.01 (0.01 to 0.02)
Bahrain	256.4 (165.6-377.9)	704.8 (460.9-1029.6)	174.9	47.9 (31-70.3)	48.3 (31.7-70.8)	0.8	0.08 (0.06 to 0.1)
Egypt	26140.2 (16825.6-38395.4)	45661.7 (29476.6-66068.9)	74.7	44.2 (28.5-65)	44.7 (29.1-64.6)	1.1	0.05 (0.04 to 0.05)
Iran (Islamic Republic of)	34582.9 (22489.4-50672.3)	47262.3 (31078.1-68539.4)	36.7	55.4 (36.2-80.6)	56.4 (37-81.7)	1.8	0.03 (0.02 to 0.05)
Iraq	8302.9 (5442.2-12178.3)	19279.9 (12620.6-28034.1)	132.2	43.5 (28.4-63.3)	43.8 (28.7-64)	0.7	0 (0 to 0.01)
Jordan	1852.5 (1201.8-2715.7)	5531.8 (3631.3-8196.9)	198.6	45.4 (29.5-67)	46 (30.1-67.9)	1.3	0.04 (0.02 to 0.06)
Kuwait	884.2 (571.6-1314.2)	2046.4 (1337-3023.8)	131.4	48.3 (31.5-71.6)	46.6 (30.6-68.5)	-3.5	-0.13 (-0.18 to -0.09)
Lebanon	1583.3 (1022.6-2339)	2373.9 (1547.2-3483.9)	49.9	46.3 (29.8-68.	46.2 (30.2-67.	-0.2	-0.01

				2)	9)		(-0.01 to 0)
Libya	2008.4 (1296-2918.6)	2933.3 (1911.3-4302.7)	46.1	44.5 (28.6-64.6)	43.7 (28.6-64.4)	-1.8	-0.02 (-0.04 to 0)
Morocco	11691.5 (7617.2-17083.5)	15796.4 (10196.7-22977.5)	35.1	43.6 (28.4-63.7)	43.9 (28.3-64)	0.7	0.03 (0.02 to 0.04)
Oman	943.1 (612.6-1367.4)	2241.8 (1472.8-3301.5)	137.7	45.6 (29.7-65.8)	46.3 (30.6-67.9)	1.5	0.1 (0.04 to 0.16)
Palestine	997.9 (647.6-1469.9)	2328.3 (1512.5-3384.4)	133.3	44 (28.7-65)	44.4 (28.9-64.4)	0.9	0.02 (0.01 to 0.03)
Qatar	238.7 (154.4-349.9)	1586.1 (1040.8-2319.9)	564.6	50.8 (33-74.5)	52.6 (34-75.9)	3.5	0.2 (0.15 to 0.24)
Saudi Arabia	8040.4 (5230.3-11744)	17169.6 (11130.5-24919.9)	113.5	47.3 (30.9-69.1)	47.4 (30.8-68.7)	0.2	0.01 (-0.01 to 0.03)
Sudan	9477.7 (6190.6-13796.4)	18949.6 (12405.4-27631.2)	99.9	43.4 (28.3-63.1)	43.7 (28.6-64.3)	0.7	0.02 (0.01 to 0.02)
Syrian Arab	6222.5 (3983-9015.3)	6323 (4171.4-9321.3)	1.6	44.6 (28.8-64.	43.2 (28.6-63.	-3.1	-0.11

Republic				6)	4)		(-0.15 to -0.06)
Tunisia	3959.7 (2570.3-5828.1)	5015.8 (3309.8-7319.1)	26.7	44.7 (29.1-65.6)	44.4 (29.3-65)	-0.7	-0.02 (-0.02 to -0.02)
Turkey	27963 (18333.6-41349.4)	35525.8 (23267.4-52159.1)	27.0	44.7 (29.3-65.8)	44.9 (29.5-66.2)	0.4	0 (0 to 0.01)
United Arab Emirates	995.3 (642.4-1447.6)	4866.7 (3120.4-7069.9)	389.0	50.2 (32.2-73.4)	50.5 (33-73.5)	0.6	0.07 (0.03 to 0.11)
Yemen	6576.3 (4326.5-9542)	14690.6 (9547.3-21262.4)	123.4	43.3 (28.6-62.4)	43.5 (28.3-62.8)	0.5	0 (-0.01 to 0)
**South Asia**	512702.2 (337802.5-748333.4)	808171.2 (530435.4-1176149.1)	57.6	44 (29-64.4)	44 (28.9-63.9)	0.0	0 (-0.01 to 0)
Bangladesh	52878.5 (34825.4-77931.1)	71746.1 (46893.7-106532.3)	35.7	45.2 (29.6-66.1)	44.6 (29.2-66.5)	-1.3	-0.06 (-0.08 to -0.05)
Bhutan	299.9 (197.3-429.8)	347.7 (226.4-501.3)	15.9	45.5 (29.8-65.2)	45.4 (29.7-65.4)	-0.2	0.01 (0 to 0.02)

India	394785.7 (259379.6-577599.3)	614506.1 (402558-899130.6)	55.7	43.6 (28.6-64)	43.7 (28.6-63.9)	0.2	0 (0 to 0.01)
Nepal	9359.7 (6147.4-13732.1)	13719.6 (8975.2-20182.7)	46.6	44.6 (29-65.6)	43.9 (28.7-64.1)	-1.6	-0.05 (-0.06 to -0.04)
Pakistan	55378.4 (36603.5-81494.4)	107851.7 (70963.8-157820.9)	94.8	45.8 (30.4-67.2)	45.3 (29.8-66.4)	-1.1	-0.04 (-0.04 to -0.03)
**Southern sub-Saharan Africa**	31424.5 (20608.1-45812.5)	45504.8 (29824.8-65835.7)	44.8	56.2 (36.7-81.3)	56.4 (37-81.4)	0.4	0.02 (0.01 to 0.03)
Botswana	784.5 (507.2-1137.8)	1365.4 (884.5-1980.6)	74.1	55.6 (36-79.9)	56.4 (36.6-81.8)	1.4	0.05 (0.04 to 0.06)
Lesotho	1082.7 (710.7-1594.4)	1221.8 (787.4-1796.8)	12.8	55.8 (36.7-82)	55.9 (36.1-82.1)	0.2	-0.01 (-0.02 to 0)
Namibia	848.4 (554.3-1223.3)	1409.4 (911.5-2042.8)	66.1	56 (36.7-81)	55.8 (36.2-80.8)	-0.4	-0.01 (-0.02 to 0)
South Africa	21879.5 (14312.2-31701.8)	31885.8 (20921.9-45879.6)	45.7	56.2 (36.9-81.5)	56.8 (37.2-81.8)	1.1	0.04 (0.03 to

							0.05)
Eswatini	490 (318.2-719.2)	676.2 (441.8-989.9)	38.0	55 (35.7-80. 7)	55.5 (36.4-81. 2)	0.9	0.02 (0.0 1 to 0.04)
Zimbabwe	6339.4 (4133.9-9323.2)	8946.3 (5833.4-12973.3)	41.1	56.2 (36.5-81. 7)	55.4 (36.2-80. 3)	-1.4	-0.0 4 (-0. 06 to -0.0 3)
**Western sub-Sahar an Africa**	117110.5 (76700.5-16903 3.3)	276572.7 (181323-400817. 7)	136.2	56.7 (37.2-81. 6)	56.2 (36.9-81. 3)	-0.9	-0.0 3 (-0. 04 to -0.0 2)
Benin	2970.2 (1942.4-4357)	7761.5 (5064.6-11182.2)	161.3	55.6 (36.5-80. 6)	56.4 (37-81.7)	1.4	0.04 (0.0 4 to 0.05)
Burkina Faso	5759.6 (3767.2-8332.1)	13767.7 (9010.6-20270.1)	139.0	54.9 (35.8-79. 5)	55.7 (36.4-81. 5)	1.5	0.05 (0.0 5 to 0.06)
Cameroon	6328.3 (4171-9095.9)	17736 (11674.7-25919.5)	180.3	56 (37-80.7)	56.7 (37.6-82. 6)	1.3	0.05 (0.0 5 to 0.05)
Cabo Verde	211.8 (137-313.6)	333.7 (217.8-492.4)	57.5	55.9 (36.5-82. 5)	58.1 (37.9-86)	3.9	0.14 (0.1 4 to 0.15)
Chad	3652.7 (2389.3-5273.8)	10191.9 (6621-14926.7)	179.0	55.6 (36.3-80.	56.7 (37-82.6)	2.0	0.07 (0.0

				4)			7 to 0.07)
Côte d'Ivoire	7568.7 (4963.3-11025.3)	16076.4 (10614.2-23548.6)	112.4	57 (37.6-83. 2)	57.6 (38.2-84. 3)	1.1	0.04 (0.0 4 to 0.05)
Gambia	610.7 (401.9-886.2)	1356.1 (886-1968.6)	122.0	57 (37.6-82. 6)	56.4 (36.9-81. 6)	-1.1	-0.0 5 (-0. 06 to -0.0 4)
Ghana	9182.6 (5959-13440.3)	18710.4 (12379.3-27302.9)	103.8	56.5 (36.6-82. 8)	56.1 (37.3-81. 9)	-0.7	-0.0 3 (-0. 04 to -0.0 2)
Guinea	3729.5 (2390-5363.4)	7657.1 (5051.5-11044.3)	105.3	56 (36.1-80. 6)	56 (36.9-80. 9)	0.0	-0.0 1 (-0. 02 to 0)
Guinea-Bissau	610.3 (400.6-883.8)	1146.8 (751.3-1646.7)	87.9	55.3 (36.3-79. 5)	55.7 (36.6-79. 9)	0.7	0.02 (0.0 2 to 0.02)
Liberia	1166.6 (770.6-1678.2)	2884.1 (1879.7-4236.9)	147.2	55.5 (36.2-79. 9)	56.7 (36.9-82. 7)	2.2	0.08 (0.0 7 to 0.09)
Mali	5253.4 (3450.8-7597)	13448.9 (8721.3-19496.5)	156.0	55.9 (36.5-81. 2)	56.6 (36.7-81. 7)	1.3	0.05 (0.0 5 to 0.06)

Mauritania	1266.7 (821.8-1840.8)	2426.3 (1570.8-3496.6)	91.5	57 (37-82.9)	57.1 (37.1-82.4)	0.2	0 (0 to 0)
Niger	4945.8 (3253-7142.2)	14507.2 (9506.4-21243.5)	193.3	56.5 (37.2-81.6)	56.5 (37-82.7)	0.0	0 (0 to 0.01)
Nigeria	54715.4 (35933.3-78512.5)	129491.9 (84898.3-188176.9)	136.7	57.3 (37.7-82.4)	55.9 (36.8-81.2)	-2.4	-0.08 (-0.1 to -0.06)
Sao Tome and Principe	74.4 (48.7-107.4)	123.8 (80.4-180.2)	66.4	56.7 (37.1-81.7)	57.4 (37.3-83.5)	1.2	0.05 (0.04 to 0.06)
Senegal	4640.3 (3013.2-6728.2)	9215.5 (5955.4-13525.3)	98.6	56.2 (36.4-81.1)	57.1 (37.4-83)	1.6	0.06 (0.06 to 0.06)
Sierra Leone	2177.4 (1433.1-3173.8)	4987.8 (3302.8-7209.4)	129.1	55.8 (36.6-80.6)	56.4 (37.5-81.8)	1.1	0.07 (0.06 to 0.09)
Togo	2242.2 (1462.1-3262.1)	4745.7 (3097.6-6849.6)	111.7	55.6 (36.4-80.8)	56 (36.7-80.7)	0.7	0.02 (0.01 to 0.02)
**Eastern sub-Saharan Africa**	118378 (77519.2-171365.1)	255581.7 (168416.4-369497.7)	115.9	57.1 (37.3-82.6)	57.4 (37.7-83.1)	0.5	0.03 (0.03 to 0.03)
Burundi	3430.8 (2233.4-4960.5)	7380.3 (4800.1-10659.4)	115.1	56.3 (36.7-81)	57.3 (37.6-82.5)	1.8	0.08 (0.06 to 0.09)

Comoros	289 (188.5-420.6)	428.7 (281.7-623.7)	48.3	57.5 (37.6-83.7)	58.1 (38.2-84.6)	1.0	0.04 (0.04 to 0.05)
Djibouti	317.5 (208.3-465)	751 (491.1-1090.8)	136.5	59.9 (39-87.4)	59.9 (39.2-86.5)	0.0	0.01 (0.01 to 0.02)
Eritrea	1867 (1226.5-2657.1)	4141.2 (2677.9-5940.5)	121.8	56.2 (36.8-80.8)	57.2 (37.3-82.3)	1.8	0.06 (0.06 to 0.07)
Ethiopia	32280.8 (21166.2-47059.3)	67478.9 (44394.9-97295.5)	109.0	57.6 (38-83.7)	58.2 (38.4-84)	1.0	0.04 (0.03 to 0.04)
Kenya	14668.1 (9595.2-21231)	31070.4 (20265.9-44989.2)	111.8	57.8 (37.8-83.5)	58 (37.8-83.9)	0.3	0.03 (0.02 to 0.04)
Madagascar	7385 (4818.8-10675.6)	16477.3 (10715.1-23844.6)	123.1	57.1 (37.4-82.7)	57.6 (37.3-83.1)	0.9	0.04 (0.03 to 0.04)
Malawi	5879.4 (3868.4-8584.8)	11297.1 (7418-16554)	92.1	56.5 (37-81.8)	56.5 (37-82.3)	0.0	0.01 (0 to 0.01)
Mozambique	7875.5 (5206.1-11371)	18101.1 (11980.9-26150.1)	129.8	55.5 (36.7-79.9)	55.8 (36.7-80.6)	0.5	0.03 (0.02 to 0.03)
Rwanda	4432.3 (2911.5-6437.6)	7678.3 (4992.6-11044.1)	73.2	56.4 (37.3-81.9)	56.6 (36.9-81.9)	0.4	0.03 (0 to

							0.07)
Somalia	4531.6 (2958.9-6554.7)	12880.2 (8445.8-18652)	184.2	57.7 (37.9-82.9)	57.5 (38.1-82.7)	-0.3	-0.01 (-0.01 to 0)
South Sudan	3713.7 (2368.9-5387.7)	5743.4 (3755.3-8325.3)	54.7	58.8 (38.1-85.2)	57.1 (37.5-82.2)	-2.9	-0.1 (-0.11 to -0.09)
United Republic of Tanzania	15824.6 (10415.8-22880.7)	34656.9 (22505.9-50586.5)	119.0	56.1 (36.9-81)	56.7 (37-81.7)	1.1	0.05 (0.04 to 0.05)
Uganda	10893.2 (7050-15878.1)	26105.8 (16928.1-37702.7)	139.7	57.3 (37.5-83.1)	57.6 (37.6-83)	0.5	0.03 (0.02 to 0.04)
Zambia	4902.6 (3181.2-7041.7)	11186.8 (7283.5-16248.9)	128.2	56.6 (36.9-81.4)	56.8 (37-81.9)	0.4	0.02 (0.01 to 0.02)
**Central sub-Saharan Africa**	33720.1 (21810.6-48808.1)	79879.2 (52612.3-116383.7)	136.9	55.7 (36.1-80.3)	56.2 (37.1-81.4)	0.9	0.03 (0.03 to 0.04)
Angola	6355 (4099.8-9316.3)	18367.1 (11847.3-26983.7)	189.0	56.7 (37.2-82)	55.8 (36.2-81.5)	-1.6	-0.06 (-0.07 to -0.04)
Central African	1645.6 (1091.8-2398.8)	3172.9 (2084.3-4577.1)	92.8	55.1 (36.5-80.	55.3 (36.5-80)	0.4	0.01 (0.0

Republic				7)			1 to 0.02)
Congo	1480.9 (966.7-2139.9)	3137.7 (2034.1-4579.4)	111.9	55.7 (36.6-81.1)	56.5 (36.6-82.1)	1.4	0.06 (0.05 to 0.06)
Democratic Republic of the Congo	23384 (15138.9-34064.8)	53280.6 (35278.6-77632.9)	127.9	55.4 (36-80.8)	56.3 (37.3-81.8)	1.6	0.06 (0.05 to 0.06)
Equatorial Guinea	257.2 (169.3-367.1)	903.7 (591.6-1306.4)	251.4	54.3 (35.8-77.9)	57.9 (38.1-84)	6.6	0.25 (0.23 to 0.26)
Gabon	597.5 (386.4-881.2)	1017.2 (665.7-1466.9)	70.2	56.5 (36.6-83.5)	55.9 (36.8-80.6)	-1.1	-0.03 (-0.04 to -0.03)

Note: ASD, autism spectrum disorders; DALY, disability-adjusted life year; GBD, Global Burden of Disease; SDI, sociodemographic index.

eTable 4 . Top 20 countries with autism spectrum disorders in 2019.

Rank	Prevalent cases	ASPR	Incident cases	ASIR	DALYs	Age-standardized DALY rate
1	China	United Kingdom	India	Andorra	China	United Kingdom
2	India	Sweden	China	United Kingdom	India	Sweden
3	United States of America	Japan	Nigeria	Sweden	United States of America	Japan
4	Nigeria	United States of America	United States of America	Japan	Nigeria	United States of America
5	Indonesia	Netherlands	Pakistan	United States of America	Indonesia	Netherlands
6	Japan	Canada	Ethiopia	Netherlands	Japan	Canada
7	Brazil	Ireland	Indonesia	Canada	Brazil	Ireland
8	Pakistan	Singapore	Brazil	Ireland	Pakistan	Singapore
9	Russian Federation	Andorra	Democratic Republic of the Congo	Brunei Darussalam	Russian Federation	Andorra
10	United Kingdom	Spain	Philippines	Singapore	Bangladesh	Spain
11	Bangladesh	Malta	United Republic of Tanzania	Spain	Mexico	Malta
12	Mexico	Switzerland	Bangladesh	Cyprus	United Kingdom	Switzerland
13	Germany	Germany	Mexico	Republic of Korea	Ethiopia	Germany
14	Ethiopia	Luxembourg	Russian Federation	Switzerland	Germany	Luxembourg
15	Philippines	Brunei Darussalam	Uganda	Italy	Philippines	Brunei Darussalam
16	Democratic Republic of the Congo	Italy	Egypt	Malta	Democratic Republic of the Congo	Italy
17	Italy	Austria	Japan	Greece	Iran (Islamic Republic of)	Austria
18	Iran (Islamic Republic of)	Iceland	United Kingdom	Austria	Italy	Iceland

19	Viet Nam	Belgium	Kenya	Portugal	Viet Nam	Greece
20	Egypt	Greece	Iran (Islamic Republic of)	Belgium	Egypt	Cyprus

Note: ASPR, age-standardized prevalence rate; ASIR, age-standardized incidence rate; DALY, disability-adjusted life year; SDI, sociodemographic index.

eTable 5. Prevalence, incidence, and disability-adjusted life year (DALYs) rates by age group in 1990 and 2019.

Rates	Age group	1999			2019		
		Val	Upper	Lower	Val	Upper	Lower
Prevalence	1-4 years	430.6	510.7	356.7	438.0	520.2	363.4
Prevalence	5-9 years	420.0	499.5	348.8	425.0	505.3	352.8
Prevalence	10-14 years	408.6	485.9	338.9	410.7	489.8	340.7
Prevalence	15-19 years	398.6	475.8	329.6	396.0	473.1	327.9
Prevalence	20-24 years	388.3	464.6	321.2	383.0	457.9	317.5
Prevalence	25-29 years	381.1	456.3	315.4	372.3	445.4	308.3
Prevalence	30-34 years	373.6	447.7	309.0	362.3	434.4	299.6
Prevalence	35-39 years	362.3	433.9	299.0	352.1	422.1	290.5
Prevalence	40-44 years	358.5	430.4	296.1	341.7	411.3	281.7
Prevalence	45-49 years	346.0	417.4	285.9	334.0	403.0	275.6
Prevalence	50-54 years	335.9	405.1	277.8	325.0	392.1	269.0
Prevalence	55-59 years	324.9	392.5	267.9	318.0	383.6	262.6
Prevalence	60-64 years	313.1	378.6	257.8	307.6	371.4	253.5
Prevalence	65-69 years	299.3	360.8	245.8	298.2	358.3	245.0
Prevalence	70+ years	271.0	326.4	221.6	280.6	338.0	229.4
Prevalence	Age-standardized	372.8	444.9	309.1	369.4	441.2	305.9
Prevalence	<1 year	435.8	519.1	362.3	444.9	530.5	369.8
Incidence	1-4 years	NA	NA	NA	NA	NA	NA
Incidence	5-9 years	NA	NA	NA	NA	NA	NA
Incidence	10-14 years	NA	NA	NA	NA	NA	NA
Incidence	15-19 years	NA	NA	NA	NA	NA	NA
Incidence	20-24 years	NA	NA	NA	NA	NA	NA
Incidence	25-29 years	NA	NA	NA	NA	NA	NA
Incidence	30-34 years	NA	NA	NA	NA	NA	NA
Incidence	35-39 years	NA	NA	NA	NA	NA	NA
Incidence	40-44 years	NA	NA	NA	NA	NA	NA
Incidence	45-49 years	NA	NA	NA	NA	NA	NA
Incidence	50-54 years	NA	NA	NA	NA	NA	NA
Incidence	55-59 years	NA	NA	NA	NA	NA	NA
Incidence	60-64 years	NA	NA	NA	NA	NA	NA

Incidence	65-69 years	NA	NA	NA	NA	NA	NA
Incidence	70+ years	NA	NA	NA	NA	NA	NA
Incidence	Age-standardized	9.2	10.9	7.6	9.3	11.1	7.7
Incidence	<1 year	458.2	545.9	381.0	457.5	545.6	380.1
DALYs	1-4 years	67.1	98.0	43.9	68.5	98.5	45.0
DALYs	5-9 years	65.4	96.0	42.9	66.3	96.7	43.4
DALYs	10-14 years	63.5	92.8	41.6	63.9	93.5	41.8
DALYs	15-19 years	61.7	89.9	40.3	61.3	89.4	39.9
DALYs	20-24 years	59.9	86.8	39.1	59.1	85.7	38.6
DALYs	25-29 years	58.4	84.2	38.3	57.1	82.7	37.3
DALYs	30-34 years	56.9	82.2	37.0	55.3	80.4	36.1
DALYs	35-39 years	54.9	78.8	35.9	53.4	76.7	34.9
DALYs	40-44 years	54.0	77.4	35.3	51.5	74.0	33.7
DALYs	45-49 years	51.7	74.7	34.0	50.1	72.1	32.8
DALYs	50-54 years	49.8	71.7	33.0	48.3	69.4	31.9
DALYs	55-59 years	47.6	69.0	31.6	46.7	67.8	31.1
DALYs	60-64 years	45.3	64.9	30.2	44.6	64.1	29.6
DALYs	65-69 years	42.6	61.7	28.5	42.5	60.9	28.6
DALYs	70+ years	37.4	53.5	25.2	38.7	55.3	26.0
DALYs	Age-standardized	56.7	82.2	37.0	56.3	81.5	36.8
DALYs	<1 year	67.9	97.8	44.5	69.5	100.9	45.5
